# Observation Moderates the Moral Licensing Effect: A Meta-Analytic Test of Interpersonal and Intrapsychic Mechanisms

**DOI:** 10.1177/01461672251345512

**Published:** 2025-07-08

**Authors:** Amanda Rotella, Jisoo Jung, Christopher Chinn, Pat Barclay

**Affiliations:** 1 Northumbria University, Newcastle-Upon-Tyne, Northumberland, UK; 2 University of Guelph, Guelph, ON, Canada

**Keywords:** moral licensing, observation, interpersonal, moral balancing, meta-analysis

## Abstract

Moral licensing occurs when someone who initially behaves morally subsequently acts less morally. We apply reputation-based theories to predict when and why it occurs. As pre-registered, we predicted: (1) being observed would be associated with larger licensing effects and (2) unambiguous outcomes would have smaller licensing effects. In a multi-level meta-analysis of 115 experiments (*N* = 21,770), moral licensing was stronger when participants were observed (*g* = 0.65) than unobserved (*g* = 0.13). After accounting for publication bias with robust Bayesian meta-analysis, there was moderate evidence for licensing when participants were observed (*g* = 0.51; *BF_10_* = 9.14), but moderate evidence against licensing when unobserved (Hedge’s *g* = −0.01; *BF_10_* = 0.11). Ambiguity did not moderate moral licensing. These findings suggest that moral licensing is elicited through interpersonal motives, clarify when licensing (vs. consistency) occurs, and explain why many online studies failed to replicate. Evidence for intrapsychic motives is inconclusive.

## Introduction

Moral licensing occurs when someone who initially behaved morally subsequently behaves less morally, as if the initial moral behavior gave them “license” to act badly. For example, if someone spent the day volunteering but then chose to litter, they would be considered to have “licensed” their littering.

This effect has been found across several domains and cultures, where people who initially behaved morally are subsequently less cooperative (Conway & Peetz, 2012; Susewind & Hoelzl, 2014), more likely to cheat (Jordan et al., 2011; Mazar & Zhong, 2010), less charitable (Conway & Peetz, 2012; Sachdeva et al., 2009), more willing to hire White candidates over minorities (Effron et al., 2009; Monin & Miller, 2001), less pro-environmental (Geng et al., 2016; Lalot et al., 2018), and more likely to lie for personal advantage (Mazar & Zhong, 2010). Moral licensing has been found in experiments (Conway & Peetz, 2012; Effron et al., 2009; Monin & Miller, 2001; Sachdeva et al., 2009), field studies (Meijers et al., 2015; Tiefenbeck et al., 2013), and in everyday life (Hofmann et al., 2014). In fact, there are hundreds of published papers on moral licensing and related effects (i.e., spillover effects; moral balancing). However, many recent moral licensing replication attempts have failed (Bahník & Vranka, 2022; Boyd, 2014; Rotella & Barclay, 2020), including high-powered attempts (Blanken et al., 2014; Urban et al., 2019).

This discrepancy is puzzling—why do some studies find an effect while others do not? Some have theorized that this effect is part of the replication crisis, where once established effects fail to be replicated in more generalizable samples (Shrout & Rodgers, 2018). We advance an alternative explanation—that methodological changes in experimental psychology, namely moving lab-based experiments online, have masked the effect because the underlying motives are *interpersonal* rather than *intrapsychic*. These motives have often been conflated in social psychology (Leary et al., 2015). That is, if moral licensing is an interpersonal effect based on reputation, effects previously observed in lab-based experiments would be attenuated or altogether disappear in online experiments because there are fewer interpersonal interactions and social cues online.

Using the existing body of moral licensing studies, we investigate if the moral licensing effect occurs in the presence or absence of others. That is, is the moral licensing an interpersonal effect based on *reputation*, or an intrapsychic effect based on *self-image?*

The overarching aim of this meta-analysis is to test the moderating effect of experimental methods on the size of the moral licensing effect. By comparing conditions in which the effect is larger (vs. smaller), we aim to generate insights into (i) which proposed mechanism underlying the moral licensing has greater support and (ii) how experimental methods impact the moral licensing effect. These insights will be of broad value to the moral regulation literature, which addresses when people choose to be more (or less) moral over time.

### Past Moral Licensing Meta-Analyses

So far, there have been four moral licensing meta-analyses. The first, conducted by Blanken et al. (2015), found an overall moral licensing effect of Cohen’s *d* = 0.31. They only identified one moderator: published studies had larger effects than unpublished studies. Simbrunner and Schlegelmilch (2017) expanded on this effort and found that culture moderated the moral licensing effect. Subsequently, Kuper and Bott (2019) tested if the moral licensing effect was inflated by publication bias by applying bias-correction (PET-PEESE, 3-PSM]) methods to meta-analytic results. The licensing effect was smaller than reported in prior efforts (PET-PEESE: *d* = −0.05, 3-PSM: *d* = 0.18; Kuper & Bott, 2019). In a recent meta-analysis, Ferguson and colleagues (2024) included both studies on moral licensing and the foot-in-the-door effects (i.e., consecutive moral behaviors). The moral licensing effect (excluding foot-in-the-door studies; discussed in *SI-A.1.4*) was small (Hedge’s *g* = 0.11; see *SI-B.6* for comparisons among meta-analyses). Although these efforts have started to clarify when moral licensing is more likely to be found, there was substantial heterogeneity left unexplained, indicating that further research is needed.

### Moral Licensing Theory

**Figure 1.**
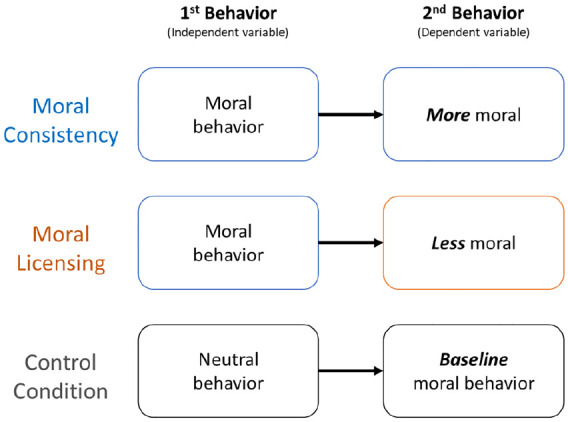
Overview of the moral licensing and moral consistency effects.

Why does moral licensing happen? In moral licensing studies, there are two sequential tasks. Participants who first completed a moral task (vs. neutral control) subsequently behave *less morally* on the second task, compared to the controls. This contrasts with well-established moral consistency theories, in which people who initially engaged in a moral act subsequently behave *more morally* on the second task, compared to controls (see Figure 1). The moral consistency literature argues that individuals display consistency in their behaviors and strive to have a consistent moral identity (e.g., self-perception theory, cognitive dissonance, balance theory).

Two causal mechanisms for moral licensing have been proposed. The first is self-image, an intrapsychic motive where a license is granted because someone proved *to themselves* that they behave morally (Conway & Peetz, 2012; Monin & Miller, 2001). This mechanism is commonly assumed in moral licensing studies. Secondly, recent papers have discussed it as a reputation-based mechanism, which is an interpersonal motive where people license because they’ve demonstrated *to others* that they are moral (Barclay, 2016; Lasarov & Hoffmann, 2020). These alternative mechanisms have been largely untested, with reputation-based stimuli having only been investigated in a single underpowered study (Monin & Miller, 2001).^1^ Further work is needed to distinguish if the moral licensing effect is elicited through interpersonal and/or intrapsychic motives. Answering this question can also lend insights into when and why we would predict a moral *licensing* instead of moral *consistency* (Figure 1; discussed in *S1-C.5*).

### How Is Reputation Related to Moral Licensing?

When people are observed, they can earn a good reputation. In turn, when people can earn a good reputation, they are more cooperative (Barclay & Willer, 2007; Milinski et al., 2002; Sylwester & Roberts, 2010). Because people have imperfect information about others, judgments are made based on what people do and say, which impacts impressions. In fact, reputation-based partner choice is important in maintaining human cooperation (Barclay, 2013, 2016; Sylwester & Roberts, 2010), where people with better reputations are more often chosen as partners (Barclay & Barker, 2020; Barclay & Willer, 2007; Sylwester & Roberts, 2010), and receive more help—even from people that they’ve not previously helped (Milinski et al., 2002; Seinen & Schram, 2006).

Given the social benefits (and consequences) of reputation, people manage perceptions by enhancing their reputation or avoiding a bad reputation, such as behaving more morally when being watched (Bradley et al., 2018; Leary & Kowalski, 1990). We posit that once a target has established a good reputation, they can behave slightly less morally and maintain a moral reputation. This is consistent with impression management research; once someone has established a good reputation, observers judge them less harshly (Effron & Monin, 2010; Polman et al., 2013), and people believe they will be judged less harshly after establishing a first impression (Effron, 2014). These findings suggest that after someone establishes a moral reputation, they can be *somewhat less moral* and still maintain a moral reputation—this saves them the cost of being fully moral all the time while keeping a good reputation (Barclay, 2016).

This interpretation is consistent with the moral licensing effect; prior meta-analyses on moral licensing have found small effects (*d*s = 0.31−0.32; Blanken et al., 2015; Simbrunner & Schlegelmilch, 2017; *d*s = −0.05- 0.18, Kuper & Bott, 2019; Ferguson et al., 2024; *g* = 0.11). We do not posit this is a large effect (e.g., people won’t steal a car after donating to charity); rather, we advance that the less moral behavior action be similarly (or less) informative for a reputation-based judgment compared to the initial moral behavior.

### Beyond Previous Meta-Analyses: New Objectives

Our primary aim is to use state-of-the-art meta-analytic strategies to test support for two mechanisms: whether moral licensing is based on self-image (an intrapsychic mechanism) and/or reputation (an interpersonal mechanism). As a secondary aim, we test if the moral licensing effect is larger with ambiguous dependent measures, based on prior theorizing (Effron & Monin, 2010; Lasarov & Hoffmann, 2020). Beyond this, we test the effect of additional methodological moderators (i.e., data collection location; monetary incentivization; licensing manipulation; participant culture) on moral licensing.

Notably, our goal is not to provide an overall estimate—previous meta-analyses (and our own) indicate that there is too much heterogeneity among studies to reliably estimate the overall effect^2^. Instead, this meta-analysis uses a novel approach: (i) we capitalize on existing differences across studies in experimental methods that have theory-based implications, (ii) test which methods are associated with larger moral licensing effects, then (iii) use these results to deduce which theory-based moral licensing mechanism is more probable, based on the amount of support in relevant conditions. This method highlights the methodological conditions that elicit moral licensing, advancing our understanding of *when* and *why* moral licensing occurs.

### Present Study

This is the first meta-analysis to systematically investigate whether the moral licensing effect varies according to observation (i.e., are participants’ decisions seen by others?) and use these insights to deduce support for an interpersonal or intrapsychic mechanism. Additionally, this is the first effort to test if the “moral ambiguity” of the dependent measure impacts the moral licensing effect. Importantly, we use the most rigorous meta-analysis methods presently available, accounting for dependencies between effect sizes and publication bias.

#### Moral Licensing Mechanism

To determine the degree of support for interpersonal or intrapsychic mechanisms, we coded methodological factors that theoretically vary for the two alternative hypotheses: degree of observation (i.e., was someone watching?) and the study location (online vs. in-person). If the mechanism is based on self-image, we predict *little-to-no difference* in the effect size based on observation or study location. In contrast, if the moral licensing effect is based on reputation, we predict that a licensing effect would persist when participants are observed or in face-to-face experiments but disappear (or be diminished) when participants are either unobserved or online. Although meta-analysis is correlational in nature, the empirical results can support for which mechanism is more plausible by identifying the conditions under which the effect varies (i.e., the effect is present/absent, smaller/larger).

#### Ambiguity of Moral Actions

Prior research has proposed that the moral licensing effect would be larger when dependent measures are morally ambiguous (Effron & Monin, 2010; Lasarov & Hoffmann, 2020). For example—imagine a participant tasked with choosing to hire a candidate for a job. They’re presented with two candidate resumes, one woman and one man. If the participant chooses to hire the man over the woman when the woman is clearly more qualified, this is unambiguously biased. But, if the participant chooses to hire the man over the women when they have nearly identical CVs, it is ambiguous whether a transgression has occurred. In the latter case, there is more room for interpreting the action generously, and the decision could be justified with many reasons, other than a biased character. Notably, this prediction is agnostic regarding whether moral licensing arises from interpersonal or intrapsychic motives, as it is applicable to both.

#### Additional Moderators

We investigate the effect of the following moderators on the size of the moral licensing effect: control condition (neutral, negative), domain of moral licensing (cooperation, environmental, discrimination), if the manipulation and dependent measure are in the same (or different) domain, type of manipulation, type of reward for the dependent measure (hypothetical or monetary), culture, and publication bias (rationales presented in *SI-A.1.2*).

## Methods

This study was pre-registered (https://osf.io/pu5hf/files/osfstorage) and all materials (data, code) are available (https://osf.io/kzwmr/files/osfstorage).

This manuscript is accompanied by two supplemental files: *Supplement I* (hereafter *SI*) provides clarifying information across all sections of the manuscript; this includes elaborations on methods (*SI.A—Methods*), analyses (*SI.B—Analysis*), discussion (*SI.C—Discussion*. Data is provided in *Supplement II* and in the repository listed above. This report satisfies the PRISMA reporting guidelines (*SI.D—PRSIMA*; Moher et al., 2009).

### Data Collection

#### Published Data

As pre-registered, studies pre-dating 2013 were extracted from previous meta-analyses (Blanken et al., 2015; Simbrunner & Schlegelmilch, 2017), and we conducted searches for papers on moral licensing dated between 2013 and 2018 on Google Scholar, PsycInfo, and Web of Science. We used the following search terms: “*moral licensing,” “moral spillover,” “psychological licensing,” “self-licensing,” “moral balancing,” “moral compensation,” “moral credentialing,” “moral credential,”* and “*moral credentials.”* To update the analysis, we identified moral licensing studies following the publication of Ferguson and colleagues (2024) meta-analysis which used similar search criteria for studies until July 2022; we extracted all relevant information from studies not previously included in this effort.^3^

#### Unpublished Data

We made calls for unpublished data to several outlets: Twitter, the Society for Personality and Social Psychology (SPSP) internet forum and mailing list (November 2017), the Human Behavior and Evolution Society (HBES; November 2017) and the European Human Behavior and Evolution Association (EHBEA; November 2017). Lastly, we searched the following conference programs (2014 to 2018) for relevant abstracts: Society for Personality and Social Psychology (SPSP), European Association of Social Psychology (EASP), International Conference on Environmental Psychology (ICEP), Association for Psychological Science (APS), Eastern Psychological Association (EPA), Midwestern Psychological Association (MPA), American Psychological Association (APA), and the Canadian Psychological Association (CPA). We emailed the authors for their study information. We considered all studies until February 15, 2019.

### Inclusion Criteria

We had four pre-registered inclusion criteria. All records were screened by the first author; decision points for study inclusion are presented in *SI-A.1*.

**1. Moral Domain.** The behaviors studied (i.e., experimental manipulation, dependent variable) must be moral. Although what is or is not moral can be subjective, we aligned our definition to what *most* people would consider moral, in accordance with two general theories of morality (i.e., moral foundations theory, Graham et al., 2013; morality-as-cooperation theory, Curry, 2016; elaboration: *SI-A.1.3*). For example, we included studies which investigated monetary donations, environmental giving, equality in hiring, cooperation, cheating, and volunteering. We excluded studies on self-regulation (e.g., willingness to exercise, consume sweets) or consumer decisions.
**2. Self-Licensing Manipulation.** We included studies where licenses were experimentally manipulated by (a) indicating intended “good” or moral behavior, (b) performing a “good” or moral action, or (c) report the recall of a past “good” or moral behavior. We excluded manipulations that should not theoretically influence a participants’ self-image or reputation, such as studies on vicarious moral licensing (e.g., Jordan et al., 2011), where participants “licensed” through others’ actions, rather than their own. Additionally, we excluded manipulations that tested boundary conditions (i.e., with no predicted difference between manipulation and control) because the theoretical prediction is that the licensing manipulation would not cause licensing (e.g., recalling an action in the distant past; see *SI-A.1.5*; Conway & Peetz, 2012). If a study included both boundary and non-boundary conditions, we only excluded the boundary condition.
**3. Experimental Design.** All studies included must have an experimental design that manipulated moral licensing. Correlational designs were excluded.
**4. Relevant Data.** Relevant data (or statistics) must be obtainable for inclusion. When relevant information was missing, we contacted the authors to request information. If we received no response after two attempts or by the cutoff date (February 15, 2019; August 12, 2024), where possible, we computed effect size estimates (e.g., compute effect size estimates by dividing the sample size evenly among conditions), otherwise the study was excluded (see excluded studies: *SI-A.1.6*, Table S2).

#### Included Studies

**Figure 2.**
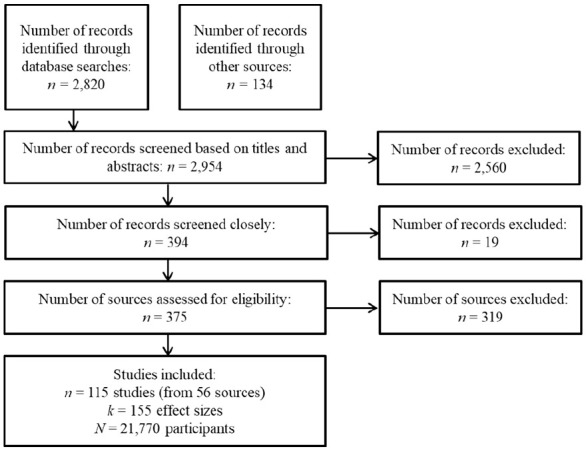
Flowchart of meta-analysis search results.

We included a total of 155 effect sizes from 115 studies with 21,770 participants (Figure 2). Of these, 88 were published and 67 were unpublished. Notably, some participants were counted twice when multiple dependent measures were coded from a single study, or multiple experimental conditions were compared to a single control condition. We account for these dependencies by using a multi-level analytic approach and cluster-robust variance estimation, nesting effect sizes within papers and accounting for dependencies between effects from the same study (see method rationales in *SI-A.2.3* and *SI-A.2.4*).

### Variable Coding

#### Coding Procedure

Coding scales were pre-registered. Two trained coders independently rated observation (weighted kappa = 0.96, *z* = 9.47, *p* < .001) and ambiguity (weighted kappa = 0.76, *z* = 7.80, *p* < .001). Both the observation and ambiguity scales were developed by the research team (see *SI-A.1.1* for full scales). Although coders were not blind to the hypotheses, we minimized bias by separating roles: two coders extracted methodological details (without statistical training), while a separate coder extracted statistical data. Although not technically blind, we can assume that method coders were ignorant of effect size information. Most papers did not report effect sizes directly; when they did, it was rarely as Cohen’s *d* or Hedge’s *g*. Instead, they provided data from which effect sizes could be calculated. Thus, we can assume that observation and ambiguity were independently assessed from effect sizes.

#### Observation

Coders used a 3-point scale:

(1) *no observation*—participants not observed or unaware of being observed;
(2) *some/unclear observation*—others present but no direct observation; and
(3) *explicit observation*—participants directly observed or aware their responses would be seen.

For example, participants alone in a lab or online were coded as *no observation*; those in shared spaces (e.g., labs or classrooms) as *some observation*; and those responding directly to the experimenter as *explicit observation*. Due to limited information or no author response, 35 studies couldn’t be coded for observation but were included in other analyses.

#### Ambiguity

Coders rated outcome ambiguity on a 4-point scale:

(1) *little-to-no ambiguity*—actions clearly diagnostic of moral character (e.g., cheating for higher payoff, hiring an unqualified white candidate over a qualified black candidate),
(2) *some ambiguity*—typically diagnostic of moral character, but with exceptions (e.g., donations to charity),
(3) *moderate ambiguity*—partially diagnostic of moral character with many exceptions (e.g., willing to fake illness to attend a concert),
(4) *high ambiguity*—actions not very diagnostic of moral character (e.g., hiring a white candidate over a black candidate, when both are equally qualified).

After coders rated each variable independently, they discussed discrepancies to reach consensus; unresolved cases were reviewed by the research team. Additional moderators (e.g., study domain, measure type, manipulation, monetary outcome, publication status, control condition, participant culture) were also extracted (see *SI-A.1.2* for rationales).

### Analyses

#### Effect Size Estimation

We calculated standardized mean differences (Cohen’s *d*) based on standard deviations (or standard errors) to calculate effect sizes. When these were not available, we converted other effect sizes, such as *t* or *χ*^2^ to Cohen’s *d*, or used pooled standard deviations where necessary (see formulas in *SI-A.2.6*). We applied Hedge’s *g* correction because Cohen’s *d* tends to inflate effect sizes for studies with low *N*s (Lipsey & Wilson, 2001). Effect sizes were coded where positive values reflect greater moral licensing.

#### Multi-Level Meta-Analyses

We computed multilevel mixed-effect meta-analyses and meta-regressions with random intercepts to account for dependent effect sizes (nested within paper and study) using the *metafor* package (Viechtbauer, 2010) in R (Team, 2016). Then, we applied cluster-robust variance estimate for correlated and hierarchical effects (i.e., CHE model) using the *ClubSandwich* package to account for non-independence of model errors (Pustejovsky & Tipton, 2022). Effects with missing data were omitted from respective analyses.

#### Publication Bias and Robust Bayesian Meta-Analysis

To our knowledge, there is no meta-analytic method that accounts for publication bias in multilevel models. To account for publication bias, we computed robust Bayesian meta-analyses (RoBMA), which estimates publication bias, but does not correct for dependencies in effect sizes. To adjust for publication bias, RoBMA computes eight distinct ways of adjusting for publication bias, which include PET, PEESE, and six weight functions. Then, models are simultaneously computed with a combination of all possible components (i.e., estimates: 2 (Effect vs. No Effect) × 2 (Heterogeneity vs. no Heterogeneity) × 9 (Publication Bias [8 models] vs. No Publication Bias), resulting in 36 different models. Then, Bayesian model averaging, based on a weighted combination of their estimates, is used to provide an overall estimate of the model which accounts for publication bias (see Bartoš et al., 2022). This method has three advantages. First, the Bayesian framework allows us to quantify the relative evidence for the null hypothesis; this distinguishes between the absence of evidence and evidence of absence. Second, model averaging allows us to base inferences based both on “normal” models and the models adjusted for publication bias, instead of basing all decisions on a single model. This is notable as there are different assumptions, strengths, and limitations to each of the different bias-correction techniques employed in meta-analysis, which are mitigated by averaging the effects (Carter et al., 2019). Thirdly, Bayesian approaches estimate the probability of an effect (and of bias), rather than making dichotomous decisions based on *p*-values. This is particularly relevant to our use: testing the probability of each mechanism. This analytic strategy combines the best available publication bias corrections, averages them to obtain an overall estimate, and returns an estimate of the probability of an effect. We report Bayesian robust meta-analyses for the main analyses.

To compute RoMBA models, we used the RoBMA package in R (Bartoš et al., 2022). We test the presence/absence of effects of interest using Bayes Factors (BFs), using default settings and priors (i.e., standard normal distribution on effect sizes, inverse gamma distribution [α = 1, β = 0.15] on heterogeneity, six weight functions and PET-PEESE publication-bias adjustment, prior probabilities of 0.50 for effect size, heterogeneity, and publication bias), which perform well in scenarios typical of psychology. To assess the influence of priors, we conducted sensitivity analyses which are reported in *SI-B.5*. We interpret Bayes Factor (BF) as: substantial evidence for an effect, values ≥ 10; moderate evidence as values between 3 and 10; weak evidence for an effect, values between 1 and 3; weak evidence against an effect, values between 1 and 1/3; moderate evidence against an effect, values between 1/3 and 1/10, substantial evidence against an effect, values ≤ 1/10 (Bartoš et al., 2022).

#### Justification for Deviations from the Pre-Registration

This analysis strategy differed from our pre-registration because it is better suited for the data structure of this meta-analysis. Multilevel models account for the non-independence of the data and RoBMA estimates publication bias. The pre-registered analyses assumed effect size independence, which was not met. Despite this difference in the analytic techniques, we (i) followed the pre-registered analysis plan to the best of our ability and (ii) conducted the pre-registered analyses (presented in *SI-B.7*). Results are consistent with those below.

All deviations from the pre-registration are justified in *SI-A.2.1*. Using these new (non-pre-registered, but more rigorous) methods, we carried out the pre-registered analysis plan. Analyses below were pre-registered, unless otherwise indicated.

## Results

### Overall Effect of Moral Licensing

#### Multilevel Meta-Analysis

First, we tested if there was an overall effect of moral licensing, using the pooled effect size of the three-level meta-analytic model. There was a small overall effect of moral licensing, Hedge’s *g* = 0.21, 95% CI [0.12, 0.29], *t*(154) = 4.87, *p* < .001; Figure 1). The estimated variance components were τ^2^_Level3_ = 0.06 _and_ τ^2^_Level2_ = 0.04. This model had very high heterogeneity, *Q(*154) = = 1982.79, *p* < .001, with the between-clusters variance estimate being 36.04% (*I*^2^_Level 3_) and the within-cluster heterogeneity was 51.13% (*I*^2^_Level 2_). This result replicated after excluding effect with negative controls (i.e., donut designs), Hedge’s *g* = 0.15, 95% CI [0.05, 0.24], *t*(114) = 2.92, *p* = .004.

#### Robust Bayesian Meta-Analysis

Then, we used RoBMA to estimate the probability of a moral licensing effect when accounting for publication bias. RoBMA indicated weak evidence for an effect, BF_10_ = 1.37, however, the averaged effect size estimate was negative^4^ (Hedge’s *g* = −0.08, 95% CI [−0.12, 0.00], heterogeneity: τ = = 0.17, 95% CI [0.13, 0.21]), which indicates weak evidence for a moral consistency effect (i.e. people are more moral after initially acting morally, compared to controls) rather than a moral licensing effect, after accounting for publication bias. See Table 1.

**Table 1.** Robust Bayesian Meta-Analysis (RoBMA) Model Summaries.

Analysis	Test	Models	Prior Probability	Posterior Probability	Hedge’s *g* (estimate)	Bayes Factor (BF)^a^
Overall model	Effect	18/36	0.50	0.62	−0.02 [−0.12, 0.00]	0.46
	Heterogeneity	18/36	0.50	1.00		7.81 × 10^11^
	Bias	32/36	0.50	1.00		1.90 × 10^9^
No observation condition	Effect	18/36	0.50	0.26	−0.01 [−0.08, 0.00]	0.11
	Heterogeneity	18/36	0.50	1.00		5.32 × 10^8^
	Bias	32/36	0.50	1.00		2515.01
Some observation conditions	Effect	18/36	0.50	0.11	−0.00 [−0.11, 0.05]	0.12
	Heterogeneity	18/36	0.50	1.00		492.72
	Bias	32/36	0.50	1.00		7715.02
**Explicit observation condition**	**Effect**	**18/36**	**0.50**	**0.90**	**0.51** [0.00, 0.79]	**9.14**
	Heterogeneity	18/36	0.50	0.28		0.38
	Bias	32/36	0.50	0.67		2.00

*Note.* Bolded values indicate effects with moderate or substantial support.

^a^Bayes Factors (BF) are interpreted as follows: ≥10 = substantial evidence for an effect; 3–10 = moderate; 1–3 = weak evidence for an effect; 1–1/3 = weak evidence against; 1/3–1/10 = moderate evidence against; ≤1/10 = substantial evidence against an effect. All model diagnostics were met (R-hat < 1.01; ESS > 500). See *SI-B.5* for analyses with alternative priors.

In this model, there was extreme evidence for heterogeneity, BF^rf^ = 4.87 × 10^23^, and publication bias, BF^pb^ = 4.68 × 10^19^. The MCMC diagnostics were good (R-hat values <1.01; ESS >500). We replicated the result using only neutral controls, again finding weak evidence of an effect, BF_10_ = 1.03, Hedge’s *g* = −0.05, 95% CI [−0.16, 0.00]; see *SI-B.4*.

We tested the sensitivity of our analysis to different prior distributions across six models, using (i) priors based on previous meta-analytic estimates (*d* = 0.31, *g* = 0.11) and (ii) recommended specifications (Bartoš et al., 2022). Four models showed evidence against an effect, while two showed weak evidence in favor of it (BF_₁₀_ = 0.23–2.27; Hedge’s g = −0.06). All models indicated strong evidence of heterogeneity and publication bias (*SI-B.5.1*).

### Moderator Analyses

Analyses below were broadly replicated with only neutral controls; see *SI-B.1*.

#### Effect of Observation

##### Multilevel Meta-Analysis

**Figure 3.**
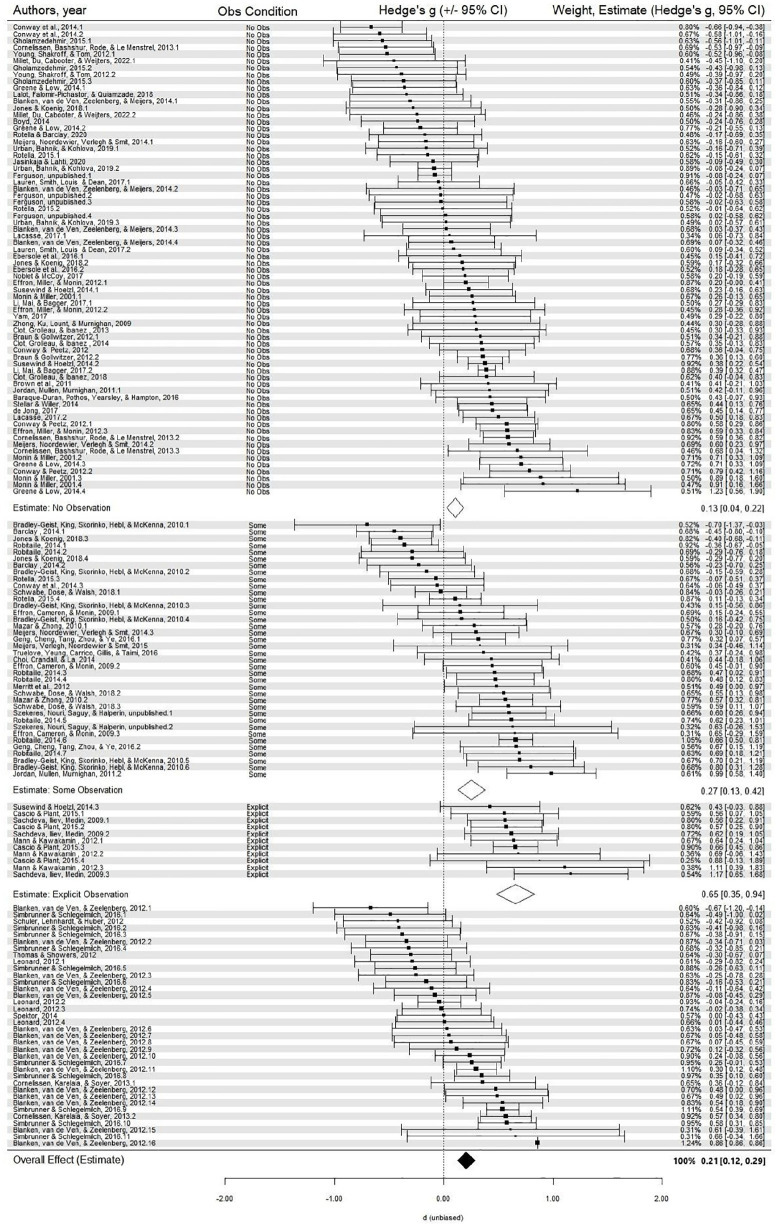
Forest plot of moral licensing effects based on multilevel models (random effect), by observation condition (white diamonds), and overall analysis (black diamond). *Note*. Obs = observation. This model does not adjust for publication bias. Effect sizes are grouped by observation condition; studies without a listed condition were included in the overall analysis. Each dot represents a study’s effect size (with CI), with dot size reflecting its analytic weight; arrows indicate CIs extending beyond the graph range. Diamonds show average effect sizes: the top indicates the estimate, width reflects the 95% CI. White diamonds represent subgroup estimates by observation condition; the black diamond shows the overall estimate.

**Figure 4.**
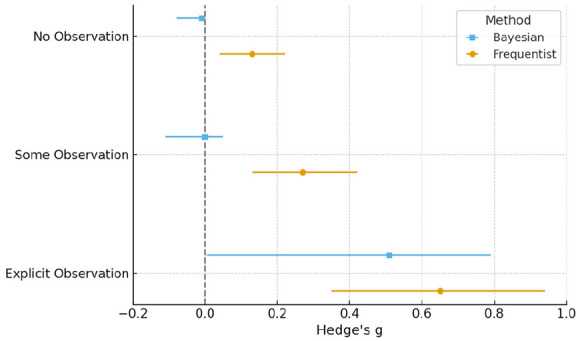
Comparison of aggregate effect size estimates from the multi-level meta-analytic models (frequentist) and robust Bayesian meta-analysis (±95% CI), by observation condition. *Note.* Multilevel analyses did not adjust for publication bias; as bias was detected, these estimates are likely inflated. Dots represent the group’s aggregate effect size. CIs indicate either 95% CI confidence intervals (frequentist) or 95% CI credible intervals (Bayesian)^5^, as applicable.

We tested if effect sizes differed according to observation. Observation moderated the moral licensing effect size, *F*(2, 116) = 6.27, *p* = .003. The smallest effect was in the *no observation* condition (Hedge’s *g* = 0.13, 95% CI [0.04, 0.22], *t*(116) = 2.73, *p* = .007), followed by the *some observation* condition (Hedge’s *g* = 0.27, 95% CI [0.13, 0.40], *t*(116) = 3.83, *p* < .001), and the largest effect in the *explicit observation* condition (Hedge’s *g* = 0.65, 95% CI [0.35, 0.94], *t*(116) *=* 4.32, *p* < .001). The effect size was significantly larger in the *explicit observation* condition compared to both the *no observation* condition and *some observation* condition, *t*(116) = −3.31, *p* = .001; *t*(116) = −2.31, *p* = .023, respectively. The effect sizes in the *no observation* and *some observation* conditions did not differ, *t*(116) = 1.71, *p* = .091; see Figure 3 and Figure 4. This pattern of results was replicated when excluding studies with differences in participant observability between the manipulation and the dependent measure (see *SI-B.2*).

Next, we hypothesized that in-person studies would elicit a greater sense of feeling observed than online studies because there are comparatively more social cues. Thus, we tested data collection location (online vs. in-person) as a proxy for observation. Location moderated the moral licensing effect, *F*(1, 117) = 7.82, *p* = .006, with a larger effect for in-person experiments (Hedge’s *g* = 0.30, 95% CI [0.19, 0.41], *t*(117*)* = 5.60, *p* < .001) compared to online experiments (Hedge’s *g* = 0.07, 95% CI [−0.02, 0.20], *t* = 1.59, *p* = .115). The effect size for online experiments did not differ from zero.

**Figure 5.**
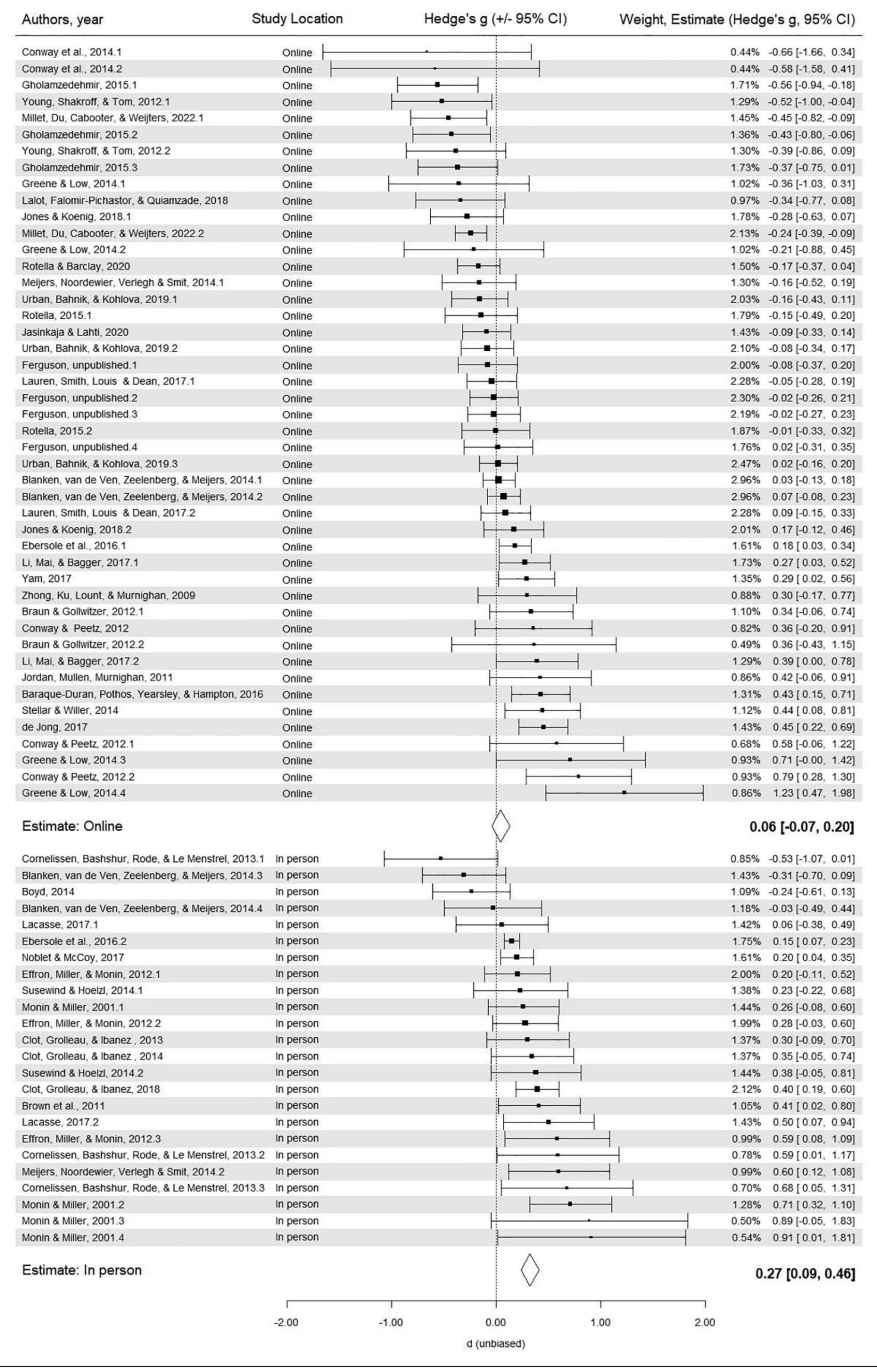
Forest plot of effect estimates in the “no observation” condition, by data collection location. *Note*. This model does not adjust for publication bias. Dots represent individual study effect sizes (with CIs); dot size reflects study weight, and arrows indicate CIs extending beyond the graph. Diamonds show average effect sizes: the top indicates the estimate, width shows the 95% CI. White diamonds reflect location-specific subsets (e.g., online, in-person); the black diamond represents the overall estimate for studies coded as *no observation*.

As noted above, there was high heterogeneity in the no observation condition. To explain some of the variability in effect sizes, we analyzed if the effect sizes in “*no observation*” condition differed according to data collection location. Even when no one is watching, we’d predict that the moral licensing effect would be smaller for studies conducted online (with little opportunity for social cues), compared to in-person studies (with more opportunity for social cues). Although the test of moderation did not reach statistical significance, *F*(1, 68) = 3.67, *p* = .060, unobserved experiments conducted in-person (i.e., lab, classrooms, field) had a significant moral licensing effect (Hedge’s *g* = 0.25, 95% CI [0.12, 0.38], *t*(68) = 3.80, *p* < .001), whereas online experiments did not (Hedge’s *g* = 0.06, 95% CI [−0.04, 0.17], *t* = 1.20, *p* = .233); see Figure 5. Although this analysis was not pre-registered, it is consistent with the pre-registered prediction that the effect would be larger with greater opportunity for observation.

##### Robust Bayesian Meta-Analysis

We applied RoBMA to estimate the probability of an effect across observation conditions (see Table 1 & Figure 4); diagnostic criteria were satisfied.

##### No Observation

In the “no observation” condition, there was weak evidence against an effect (BF_10_ = 0.36), with substantial evidence for both publication bias (BF^pb^ = 2.16 × 10^7^) and heterogeneity (BF^rf^ = 2.25 × 10^17^). The model-averaged effect size estimate was Hedge’s *g* = −0.02, 95% CI [−0.10, 0.00], and heterogeneity estimate was τ = 0.17, 95% CI [0.12, 0.22]; this result was robust to sensitivity analyses (BF_10_*s*: 0.04 to 0.23; Hedge’s g: −0.01 to 0.00; *SI-B.5.2*).

##### Some Observation

Similarly, results from the “some observation” condition indicated moderate evidence against an effect (BF_10_ = 0.19) and substantial evidence for publication bias (BF^pb^ = 2.22 × 10^8^) and heterogeneity (BF^rf^ = 1.22 × 10^5^). The model-averaged effect size estimate was Hedge’s *g* = −0.01, 95% CI [−0.14, 0.00], and heterogeneity estimate was τ = 0.22, 95% CI [0.15, 0.29]; this pattern was broadly replicated in sensitivity analyses (BF_10_*s*: 0.28 to 1.32; Hedge’s *g*: 0.01 to 0.06; see *SI-B.5.3*).

##### Explicit Observation

Contrastingly, there was moderate evidence in favor of an effect (BF_10_ = 9.14) for explicit observation, with 9.14 times more evidence in favor of a moral licensing effect compared to a null model. There was weak evidence of bias (BF^pb^ = 2.00), and weak evidence against heterogeneity (BF^rf^ = 0.38). The resulting model-averaged effect size estimate was Hedge’s *g* = 0.51, 95% CI [0.00, 0.79], heterogeneity estimate, τ = 0.03, 95% CI [0.00, 0.22]. This result was replicated in five out of six sensitivity analyses (BF_10_*s*: 0.73 to 18.88; Hedge’s *g*: 0.22 to 0.47; *SI-B.5.4*).

#### Meta-Regression: Observation, Study Location, and Publication Bias

Furthermore, to identify which moderator had the most influence, we tested the relative influence of moderators in a multilevel meta-regression analysis. We included the three significant moderators: observation condition (no/some/explicit observation), study location (online/in-person), and publication status (published/unpublished; discussed below). The model was significant, *F*(4,107) = 5.39, *p* < .001, with evidence of heterogeneity (*QE* = 249.17, *p* < .001).

Observation moderated moral licensing; larger licensing effects were found in the *explicit observation* condition compared to *no observation* condition, *t*(107) = 2.66, *p* = .008; the *no-* and *some observation* conditions did not differ, *t*(107) = 1.44, *p* = .152. Study location was not significant, *t*(107) = −0.35, *p* = .721, suggesting that explicit observation has a greater influence on moral licensing effect sizes compared to study location. Publication status was also significant, *t*(107) = 3.01, *p* = .003, demonstrating an independent effect of publication bias and explicit observation on the moral licensing effect size. These results were replicated in analyses: (i) excluding publication status, and (ii) including manipulation type and control condition (see *SI-B.6*). Although these analyses were not pre-registered, they are consistent with our pre-registered predictions.

#### Methodological Moderators

We examined whether the moral licensing effect was moderated by outcome ambiguity, publication status, control condition type, study domain, and manipulation type. All analyses were conducted using random effects multilevel meta-analyses; see Table 2 for effect size estimates. Analysis rationales are provided in *SI-A.1.2;* discussion of results in *SI-C.4*.

**Table 2.** Moderator and Subgroup Analyses of Moral Licensing, using Multi-Level Meta-Analysis.

	Estimates from Multi-Level Models		Descriptives	
Moderator	Moderator Test (Gray) Effect size (*g*) [95% CI]	Test of Effect Size (vs. Null Model)	*k*	*N*
Observation*	***F*(2, 116)** = **6.27, *p*** = **.003**		119	18,997
No observation	**0.13** [0.04, 0.22]	*t*(116) = 2.73, *p* = .007	70	15,313
Some observation	**0.27** [0.13, 0.42]	*t*(116) = 3.83, *p* < .001	38	3,138
Explicit observation	**0.65** [0.335, 0.94]	*t*(116) = 4.32, *p* < .001	11	546
***Ambiguity***	*F*(3, 140) = 0.78, *p* = .509		144	20,878
Little-to-no	**0.18** [0.02, 0.34]	*t*(140) = 2.21, *p* = .029	23	2,940
Some	**0.15** [0.05, 0.26]	*t*(140) = 2.90, *p* = .004	67	9,644
Moderate	0.13 [−0.04, 0.30]	*t*(140) = 1.49, *p* = .138	20	2,480
High	**0.27** [0.13, 0.42]	*t*(140) = 3.67, *p* = .001	34	5,814
Study location*	***F*(1, 117) = 7.82, *p*** = **.006**		119	18,997
In-person	**0.30** [0.19, 0.41]	*t*(117) = 5.60, *p* < .001	50	10,056
Online	0.09 [−0.02, 0.20]	*t*(117) = 1.59, *p* = .115	69	8,941
Publication status***	***F*(1, 153)** = **9.11, *p*** **=** **.003**		155	21.770
Unpublished	0.02 [−0.11, 0.15]	*t*(153) = 0.25, *p* = .806	67	6,478
Published	**0.26** [0.16, 0.35]	*t*(153) = 5.44, *p* < .001	88	15,292
***Control condition***	*F*(1, 153) = 2.13, *p* = .145		148	19,335
Neutral	**0.14** [0.04, 0.24]	*t*(153) = 2.89, *p* = .004	115	14,404
Negative	**0.26** [0.12, 0.40]	*t*(153) = 3.72, *p* < .001	40	7,366
***Same vs different domain*** ^a^	*F*(1, 142) = 0.56, *p* = .455		144	20,878
Same	**0.17** [0.08, 0.26]	*t*(142) = 3.72, *p* < .001	105	16,786
Different	**0.23** [0.08, 0.37]	*t*(142) = 3.13, *p* = .002	39	4,092
***Across domains***	*F*(2, 101) = 0.16, *p* = .850		104	16,722
Cooperation	**0.16** [0.04, 0.28]	*t*(101) = 2.56, *p* = .012	63	7,700
Environmental	0.13 [−0.09, 0.35]	*t*(101) = 1.15, *p* = .255	13	3,712
Discrimination	**0.21** [0.03, 0.38]	*t*(101) = 2.30, *p* = .023	28	5,310
***Manipulation type***	*F*(4, 114) = 2.15, *p* = .079		119	18,444
Imagined	**0.24** [0.04, 0.44]	*t*(114) = 2.36, *p* = .020	20	1,781
Writing prime	0.01 [−0.17, 0.18]	*t*(114) = 0.11, *p* = .915	22	4,334
Intended behaviors	**0.53** [0.13, 0.92]	*t*(114) = 2.65, *p* = .009	5	553
Recall	**0.18** [0.03, 0.33]	*t*(114) = 2.42, *p* = .017	30	4,463
Behaviors	**0.26** [0.14, 0.38]	*t*(114) = 4.14, *p* < .001	42	7,866
Participant culture***	***F*(5, 140)** **=** **4.55, *p* < .001**		146	15,787
North America	**0.26** [0.15, 0.37]	*t*(140) = 4.61, *p* < .001	81	9,399
Europe	**0.15** [0.03, 0.32]	*t*(140) = 2.32, *p* < .001	51	5,039
United Kingdom	−0.45 [−1.04, 0.13]	*t*(140) = −1.52, *p* = .130	3	334
Australia	0.02 [−0.57, 0.61]	*t*(140) = 0.08, *p* = .937	2	548
Asia	**−0.36** [−0.65, −0.08]	*t*(140) = −2.51, *p* = .013	7	707
Africa	0.32 [−0.19, 0.83]	*t*(140) = 1.26, *p* = .211	2	467
***Monetary DV***	*F*(1, 107) = 0.75, *p* = .388		109	17,474
Hypothetical	**0.21** [0.12, 0.31]	*t*(107) = 4.65, *p* < .001	85	14,293
Monetary	0.15 [−0.01, 0.30]	*t*(107) = 1.91, *p* = .059	24	3,181

*Notes.* Bolded values indicate that the effect size estimates were significantly different than zero.

* indicates significance at *p* < .05, ** and *** at *p* < .001. Moderator test results and effect size estimates (with 95% CI confidence intervals) are presented under the “moderator test” column, while tests determining if the subgroup is significantly different than zero are presented under “effect size tests.” All effect sizes are presented as Hedge’s *g*.

a This analysis only contained studies in which the IV and DV were in the same domain.

##### Ambiguity

We investigated if the ambiguity of the dependent measure (i.e., no, low, moderate, or high ambiguity) moderated the moral licensing effect. There was no effect of ambiguity, *F*(1, 140) = 0.78, *p* = .509. As pre-registered, we compared the extreme values of ambiguity (i.e., no ambiguity vs high ambiguity); again, ambiguity did not moderate moral licensing, *F*(1, 55) = 0.65, *p* = .423.

##### Publication Status

Publication status moderated the moral licensing effect, *F*(1, 153) = 9.11, *p* = .003, with published experiments having larger effect sizes (Hedge’s *g* = 0.26, 95% CI [0.16, 0.35], *t*(153) = 5.44, *p* < .001) compared to unpublished experiments (Hedge’s *g* = 0.02, 95% CI [−0.11, 0.15], *t*(153) = 0.25, *p* = .806).

##### Control Condition

The type of control condition (i.e., neutral vs negative controls) did not moderate the size of the moral licensing effect, *F*(1, 153) = 2.15, *p* = .145.

##### Moral Licensing Domain

The domain of moral licensing (i.e., cooperative, environmental, discrimination) did not moderate moral licensing, *F*(3, 101) = 0.16, *p* = .921.

##### Domain Consistency

Domain consistency for the licensing manipulation and dependent measure did not moderate the size of the moral licensing effect, *F*(1, 142) = 0.56, *p* = .455. See *SI-C.2* for discussion of this result.

##### Manipulation Type

Moral licensing can be induced through various manipulations, including: imagining a moral act (imagined actions), completing a moral prime (e.g., writing moral words and reflecting on their meaning; writing primes), reporting intended moral behavior (intended actions), recalling a past moral act, or performing an actual moral behavior (e.g., donating to charity). The results of the moderation analysis indicate that the type of manipulation did not moderate the moral licensing effect, *F*(4, 81) = 1.74, *p* = .149. However, there are differences in effect size estimates among these manipulations; see Table 2.

##### Participant Culture

Participant culture moderated moral licensing in a previous meta-analysis (Simbrunner & Schlegelmilch, 2017). We attempted to replicate this analysis using a broader range of studies; participant culture moderated the moral licensing effect, *F*(5, 140) = 4.55, *p* = .001. However, the results deviate from cultural patterns (e.g., North America aligns with continental Europe but contrasts with the UK). These findings should be interpreted with caution; they are not meaningful because (i) the small number of studies and (ii) confounding between cultural and methodological factors. Studies within the same culture are often conducted by the same labs using similar methods (e.g., manipulations, measures, samples), making cultural interpretations problematic.

We replicated the analysis including only studies from North America and Europe (k > 10 for each); there was no difference among these two cultures, *F*(1, 130) = 1.52, *p* = .221. This exploratory analysis was not pre-registered.

##### Monetary DV

Incentivizing studies with real money can impact study results (Rotella et al., 2019). Thus, we investigated if effect sizes differed among incentivized and non-incentivized studies. Monetization did not moderate the effect, *F*(1, 107) = 0.75, *p* = .388.

## Discussion

In this pre-registered meta-analysis, we (1) systematically investigated whether the moral licensing effect varies according to observation (i.e., are participants’ decisions seen by others?) to deduce support for an interpersonal or intrapsychic effect and (2) tested if the “moral ambiguity” of the dependent measure impacts the moral licensing effect.

### Moral Licensing: An Interpersonal or Intrapsychic Effect?

Based on the results presented in this paper, we conclude that there is strong support for moral licensing as a social, interpersonal effect, elicited through reputational mechanisms (e.g., observation). On the other hand, the support for moral licensing as an intrapsychic effect elicited through self-image, is inconclusive. This interpretation is grounded in four key findings:

First, we tested if the moral licensing effect size varied according to three conditions: (i) *explicit observation*, where participants reported to the experimenters or received feedback to their responses, (ii) *some observation*, where participants were in the presence of others but did not interact, and (iii) *no observation*, where participants were alone. Results supported our pre-registered hypothesis: there was a larger moral licensing effect when participants were observed. Across analyses, there was stronger support for moral licensing when participants were observed (medium effect estimates: Hedge’s *g* = 0.51 to 0.65; BF = 9.14), compared to unobserved (null to small estimates: Hedge’s *g* = −0.01 to 0.13; BF = 0.11). In fact, in the multilevel meta-analyses, observation moderated the moral licensing effect, where there was a larger effect in the *explicit observation* condition (Hedge’s *g* = 0.65) compared to the *no observation* condition (Hedge’s *g* = 0.13). The effect size estimate for the *some observation* condition (Hedge’s *g* = 0.27) fell neatly between the two extreme categories. This result was robust in analyses that (i) excluded negative controls and (ii) used a two-level (non-multilevel) model (i.e., the pre-registered analysis).

Second, Bayesian analyses revealed moderate evidence for an effect only in the *explicit observation* condition (over 9 times more evidence in favor of an effect, compared to no effect), with this finding remaining robust in 5 out of 6 sensitivity analyses (83%). In contrast, Bayesian analyses of the *no-* and *some-observation* conditions showed either weak evidence for an effect or moderate evidence against one—providing limited support for the moral licensing effect in these conditions.

These complimentary methods reveal the same pattern of results: strongest support for studies that were explicitly observed, weaker support for studies that were not observed. More confidence should be placed in the (more robust) Bayesian results, because: (i) the multilevel model results are likely overestimates given that publication bias was identified as an issue^6^, (ii) unlike the multilevel models, RoBMA corrects for publication bias, (iii) RoBMA computes 36 models with different bias correction techniques, then averages across them (analytically stronger than computing single model correcting for specified multilevel dependencies), and (iv) these results were broadly consistent across sensitivity analyses. Importantly, these results do not imply that there is no intrapsychic component to the effect (discussed further below). Rather, we can only infer that moral licensing is unlikely to be systematically elicited across (methodologically variable) studies with limited observation.

Third, we found that in-person studies—where participants are exposed to more social cues (i.e., a proxy of observation)—showed a larger licensing effect than online studies. Notably, even among studies coded as unobserved, a moral licensing effect emerged in in-person contexts but was absent in online contexts. Given that in-lab settings generally have more social cues, location can be treated as a rough proxy for the level of observation. These effects will be driven by the same underlying mechanism.

Lastly, in meta-regressions, *explicit observation* predicted moral licensing effects beyond study location, publication bias, and manipulation type in meta-regressions.

Taken together, these findings provide converging and analytically robust evidence that the moral licensing effect is larger when individuals are observed, while evidence for the effect in unobserved contexts is weak and inconsistent. Based on this evidence, we can conclude there is strong support for moral licensing as a social effect elicited through interpersonal motives. If the effect was solely elicited through intrapsychic motives, effect sizes would not vary according to observation. By understanding the process driving moral licensing, we can predict the circumstances that favor licensing over consistency, which bridges these two literatures (see discussion in *SI-C.5*).

We must note that the *explicit observation* condition result was based on 11 effects. There is no universally agreed-upon minimum number of studies required in meta-analysis; however, 2–10 effects are often recommended as a minimum (Lipsey &Wilson, 2001). Although 11 studies are at the low end, it is sufficient to meta-analyze. Importantly, in analyses with fewer studies, results are more reliable when effect sizes are consistent across studies, there is low heterogeneity among studies, and there are sufficient participants. All three conditions are met in this case, with 546 participants across the included studies. Importantly, results were analytically robust across four frequentist analyses (i.e., three multilevel models: main text, excluding negative controls [*SI-B.1*], excluding studies with change in observation [*SI-B.2*]; two-level model [*SI-B.6*]), seven Bayesian analyses (main text, analysis excluding negative controls [*SI*-*B.4*], five sensitivity analyses [*SI*-*B.5.4*]), and three meta-regressions (main text, *SI-B.6*). Additionally, we conceptually replicated this finding by comparing the location of studies (in-person, online)—where only studies conducted in-person within the *no observation* condition had a significant effect. Combined, these lines of evidence suggest that this is a robust effect.

### Does Moral Ambiguity Impact the Moral Licensing Effect?

Next, we hypothesized that moral licensing would be stronger when dependent variables were morally ambiguous, as suggested by prior work (Effron et al., 2009; Effron & Monin, 2010; Lasarov & Hoffmann, 2020; Merritt et al., 2010; Monin & Miller, 2001; Mullen & Monin, 2016). Ambiguity makes it difficult to judge actions as clearly moral or immoral, potentially allowing greater justification for licensing. Contrary to this prediction, ambiguity did not moderate the moral licensing effect. Notably, however, meta-analysis does not systematically vary ambiguity conditions, and coding relied on the interpretation of the actions by coders which may differ from the interpretations by participants. Further experimental research is needed to clarify the role of ambiguity in moral licensing.

### Caution: Interpreting the Overall Effect of Moral Licensing

Lastly, we tested the overall effect of moral licensing. Multilevel results indicated a small overall effect (Hedge’s *g* = 0.21), however, this result is of limited value because (1) the multilevel result is likely an over-estimate (i.e., the analysis did not correct for publication bias), (2) RoBMA results indicated weak evidence against the existence of an effect, and (3) there was strong evidence of heterogeneity, indicating that there are substantial differences among studies. This indicates that the moral licensing literature is highly heterogeneous, therefore interpreting the overall effect without accounting for methodological moderators may not be meaningful (i.e., comparing apples and oranges). Future work investigating the heterogeneity of this literature (e.g., methods and sample moderators) is needed.

### On Moral Licensing: Further Theorizing

#### How Does a Social Moral Licensing Effect Work?

In the present study, we find support for moral licensing as a social, interpersonal effect. That is, once somebody demonstrates to another person (not just themselves) that they behave morally, they behave less morally. But how does this work?

We speculate that once a moral reputation is established, a target could subsequently behave slightly less morally (but not so uncooperative that they are judged as immoral or deemed a moral hypocrite) in a similar situation/action and still maintain a moral reputation. We theorize that this is because maintaining a reputation is less costly (e.g., time, effort, money) than first creating one. In other words, for those with an established moral reputation, moderately consistent behavior may be enough to sustain it, whereas individuals without such a reputation must demonstrate stronger moral actions to be seen as cooperative by observers. This is consistent with research finding that targets are judged less harshly after they have established a good reputation (Effron & Monin, 2010; Polman et al., 2013). It’s important to note that in these studies, the participants and observers were not previously acquainted. Moral licensing may operate differently with previous acquaintance, however, one study found moral licensing among friends (Polman & Lu, 2022).

Notably, we take a functional approach in this paper, where we argue that “a good reputation” is the ultimate function underlying the moral licensing effect, but this does not mean that people consciously seek a good reputation. Rather, it is generally beneficial to an individual if others in their environment have a positive impression of them. We argue that “observation” is a situational factor that drives the moral licensing effect: the situational factor (observation) triggers any number of proximate psychological mechanisms (e.g., conscious concern for reputation, psychological closeness, social emotions) which cause us to act in a way that we ultimately benefit from because of a good reputation. Greater opportunities for social interaction naturally create more opportunities for others to form character judgments—such as assessments of likability, friendliness, or morality—incentivizing reputation-based interpersonal motives.

#### Can Moral Licensing be Elicited by Internal Psychological Processes?

These results beg the question—is there an intrapsychic component to moral licensing? The results from the *no-* and *some-observation* conditions are inconclusive, however, these studies were not designed to directly test intrapsychic mechanisms.

On speculative reflection, why should we expect people to spontaneously reflect on their self-image after a moral act? Given the considerable variability in attention and cognition across individuals and circumstances (Judd et al., 2024), it is unlikely that behaving morally would automatically and consistently prompt reflections of moral identity across all individuals–they can think about any number of things. That said, it is possible that moral licensing can be elicited when participants are prompted to think about self-image. Our results suggest this possibility; we found small effects when participants imagine performing a moral action or express intention of performing one. Further work is needed to assess the role of intrapsychic mechanisms.

### Recommendations for Future Research on Moral Licensing

Here, we offer methodological recommendations for future moral licensing experiments.

#### Effect Size for Power Analyses

The multilevel effect size estimates should be interpreted with caution, as publication bias was detected and could not be corrected, likely inflating the results. Therefore, we recommend using the Bayesian estimates as a more reliable basis for conducting power analyses in future experiments.

#### Designing for Reputational Cues

When designing future studies, we recommend implementing procedures that include social incentives. For example, in some of the included studies, participants responded directly to the experimenter or received feedback on their responses. Reputation-based cues should remain consistent throughout both the manipulation and the dependent measure to preserve reputational context. Additionally, researchers should include manipulation checks to assess whether participants are aware of the presence of others and/or whether others gain information about their actions.

Importantly, the same observers should be present during both the manipulation and dependent measure—if the observer changes, the target may need to re-establish a new reputation. Researchers should also consider reputation when planning experiments: Who is watching? How would consecutive decisions in the tasks (both manipulation and DV) impact reputations? Adding reputation may be difficult in online experiments, but not insurmountable (see Dhaliwal et al., 2022).

#### Control Conditions

Lastly, we recommend that researchers use neutral over negative controls; neutral controls assess baseline behaviors, whereas negative controls are a different experimental manipulation (“donut design,” Mullen & Monin, 2016).

### Why This Meta-Analysis Matters: Strengths and Contributions

#### Reconciling Inconsistencies: How Methods Changes Can Mask Effects

This paper addresses a perplexing inconsistency in the moral licensing literature—why do some studies find a moral licensing effect, while others do not? One possible explanation is the replication crisis, where established effects fail to replicate in new studies with better methods (stricter research practices; bigger, more representative samples). We propose an alternative account: that recent changes in psychological research methods—particularly the move from the lab to online platforms—have masked the effect because it is a social effect, elicited in interpersonal contexts with reputation-based cues, rare in online contexts. Our findings support this interpretation.

This highlights an important implication—that experimental contexts can have notable impact on social effects. Online methods can systematically under-detect or under-estimate reputation-based effects, raising important concerns about false negatives. When studies fail to incorporate reputational or social-contextual elements—such as observation or face-to-face interaction—interpersonal effects that are robust in real-world or lab-based settings may go undetected. This methodological mismatch can lead to erroneous conclusions that certain psychological phenomena do not exist, when they are simply not being activated in online conditions. As such, the shift toward online experimentation, while valuable for scale and efficiency, may inadvertently inflate null results for socially contingent processes—contributing to an underestimation of key social mechanisms in psychological theory.

#### Advancing Understanding of Moral Licensing

This is the first meta-analysis to systematically investigate the role of interpersonal (reputation) and intrapsychic (self-image) motives in moral licensing. By reframing the moral licensing effect through the lens of social context, this study challenges the assumption that moral licensing is primarily a self-regulatory process. Instead, our findings suggest that the effect is significantly stronger when reputational cues are present—such as being observed—highlighting the importance of interpersonal cues.

By identifying the conditions under which moral licensing is likely to emerge, this work bridges the moral licensing and moral consistency literatures, predicting when and why individuals will behave *more* or *less* morally following an initial moral act, also linking moral licensing to broader theories of moral self-regulation and impression management.

#### Leveraging Method Variability for Theory Testing

Lastly, this study employs an innovative meta-analytic approach, leveraging existing methodological variation across studies that have theory-based implication, then compares the strength of evidence—both in terms of effect size and probability—for competing mechanisms. This approach offers a novel pathway for testing theoretical frameworks using existing data; with limitations (discussed below).

### Limitations and Directions for Future Research

#### Design of the Original Studies

An important limitation is that the original studies were not designed to assess our variables of interest. To address this, we developed and applied coding schemes for observation and ambiguity. Although imperfect, interrater agreement suggests that these judgments were not arbitrary. Moreover, we used observation as a proxy for how observed people felt, based on the (hopefully reasonable) assumption that people who actually are observed also feel more observed than people who are anonymous.

#### Meta-Analytic Limitations

Meta-analysis is a correlational approach—these results cannot be interpreted as causal. Nevertheless, correlational approaches are widely used in psychology because they can generate meaningful insights. In this paper, we assess support for each theory by interpreting the strength of relationships (i.e., effect sizes) and estimated probability of an effect (i.e., Bayes Factors) in theoretically informed conditions. To assess causation high-powered pre-registered experimental efforts are required.

Because meta-analysis combines results from studies that differ in methods (e.g., samples, procedures, outcomes) there are too few studies to test variable interactions. Thus, we cannot assess whether experimental features influence sizes within or across observation conditions. That said, observation is the most consistent distinguishing factor across analyses; each level included studies that varied in manipulation, domain, and outcomes, the effect of observation is larger than any other factor assessed and predicts variance beyond other moderators. So, if there were a confound, it could not “explain away” the effect of observation.

#### Heterogeneity in Moral Licensing Research

Substantial heterogeneity was identified in analyses (overall effect, *no observation*, and *some observation* conditions), indicating that there is substantial variability among the studies. Thus, there are other important differences in moral licensing studies, beyond the present moderators. Additionally, we did not code for study quality (e.g., internal/external validity, methodological rigor). Although random-effects meta-analysis accounts for heterogeneity, which includes quality differences, including quality assessments would strengthen future work.

#### Continued Theory Development

Although we tested reputation-based mechanisms, testing *how* these decisions are made was beyond the scope of this paper. Future work should examine exactly how reputation-based mechanisms operate in the context of moral licensing. For example, we do not know if moral licensing operates through moral credits or credentials, or both (Effron & Monin, 2010; Merritt et al., 2010; Miller & Effron, 2010; see *SI*, C.6). Lasarov and Hoffmann (2020) outline many predictions about social moral licensing effects, which include the effects of construal, moral hypocrisy, and moral stake for individuals and group-based behaviors. In this paper, we only examine a few predictions that could be derived from a social moral licensing perspective.

## Conclusion

From this systematic effort with 115 effects and more than 20,000 participants, we found robust support that the moral licensing effect is elicited in contexts where participants are observed, which is consistent with the interpretation that moral licensing is an interpersonal effect. We estimate a medium effect (Hedge’s *g* = 0.51 to 0.65) in conditions where participants are observed. However, the results were inconclusive in determining whether the moral licensing is elicited through intrapsychic mechanisms (e.g., self-image). The results from this meta-analysis address why online moral licensing studies may fail to replicate and advance our understanding of when and why we would expect moral licensing compared to moral consistency.

## Supplemental Material

sj-xlsx-1-psp-10.1177_01461672251345512 – Supplemental material for Observation Moderates the Moral Licensing Effect: A Meta-Analytic Test of Interpersonal and Intrapsychic Mechanisms Supplemental material, sj-xlsx-1-psp-10.1177_01461672251345512 for Observation Moderates the Moral Licensing Effect: A Meta-Analytic Test of Interpersonal and Intrapsychic Mechanisms by Amanda Rotella, Jisoo Jung, Christopher Chinn and Pat Barclay in Personality and Social Psychology Bulletin

sj-docx-2-psp-10.1177_01461672251345512 – Supplemental material for Observation Moderates the Moral Licensing Effect: A Meta-Analytic Test of Interpersonal and Intrapsychic Mechanisms Supplemental material, sj-docx-2-psp-10.1177_01461672251345512 for Observation Moderates the Moral Licensing Effect: A Meta-Analytic Test of Interpersonal and Intrapsychic Mechanisms by Amanda Rotella, Jisoo Jung, Christopher Chinn and Pat Barclay in Personality and Social Psychology Bulletin

## Notes

1. This study had 35 participants across three conditions. Based on prior meta-analytic estimates of moral licensing (*d* = −0.05 to 0.32), there was a maximum of 11.7% power to detect an effect.

2. High statistical heterogeneity indicates that estimated effects have substantial differences across studies.

3. This update was not pre-registered; on reviewer request.

4. Positive effect sizes indicate moral licensing; negative effects indicate moral consistency.

5. Asymmetrical Bayesian intervals reflect the shape of posterior distributions, which can be skewed (based on data, model robustness assumptions, prior distributions). RoBMA estimates are averaged across 36 models.

6. Publication bias is more problematic in these studies, compared to the dependencies between studies.
