# Supplementary material for: Observation Moderates the Moral Licensing Effect: A Meta-Analytic Test of Interpersonal and Intrapsychic Mechanisms
Source: Pers Soc Psychol Bull. 2025 Jul 8;52(9):2792–808. doi: 10.1177/01461672251345512 (PMC13392179; doi:10.1177/01461672251345512)
Supplement: sj-docx-2-psp-10.1177_01461672251345512 – Supplemental material for Observation Moderates the Moral Licensing Effect: A Meta-Analytic Test of Interpersonal and Intrapsychic Mechanisms [file sj-docx-2-psp-10.1177_01461672251345512.docx]

Supplement I

(*SI*)

Observation Moderates Moral Licensing:

A Meta-Analytic Test of Interpersonal and Intrapsychic Mechanisms

Table of Contents

[A – Methods 4](#_Toc197002016)

[A.1 – Materials and Procedures 4](#_Toc197002017)

[A.1.1 – Coding: Observation and Ambiguity Scales 4](#_Toc197002018)

[A.1.2 – Rationale for Moderator Analyses 6](#_Toc197002019)

[A.1.3 – Rationale for Inclusion Criteria: What is ‘Moral’? 9](#_Toc197002020)

[A.1.4 – Rationale for Exclusion: Other Sequential Moral Effects 10](#_Toc197002021)

[A.1.5 – Rationale for Exclusion: Boundary Conditions 12](#_Toc197002022)

[A.1.6 – List of Excluded Studies 13](#_Toc197002023)

[A.1.7 – Rationales for Treatment of Experimental Moderators 20](#_Toc197002024)

[A.1.8 – Differences Among Meta-Analyses on Moral Licensing 24](#_Toc197002025)

[A.2 – Analytic Decisions 26](#_Toc197002026)

[A.2.1 – Deviations From the Pre-Registration 26](#_Toc197002027)

[A.2.2 – Assessing Publication Bias 28](#_Toc197002028)

[A.2.3 – Rationale for Multilevel Models 28](#_Toc197002029)

[A.2.4 – Rationale for Cluster-Robust Variance Estimate (CHE) 29](#_Toc197002030)

[A.2.5 – Rationale against using RoBMA moderation analyses 29](#_Toc197002031)

[A.2.6 – Formulas for Effect Size Calculation 30](#_Toc197002032)

[B – Results 32](#_Toc197002033)

[B.1 – Frequentist Analyses: Neutral Controls Only 32](#_Toc197002034)

[B.2 – Frequentist Analyses: Excluding Studies with Change in Observation 34](#_Toc197002035)

[B.3 – Frequentist Analyses: With Change in Observation 35](#_Toc197002036)

[B.4 – Bayesian Analyses: Neutral Controls Only 36](#_Toc197002037)

[B.5 – Bayesian Sensitivity Analyses 37](#_Toc197002038)

[B.5.1 – Sensitivity Analyses: Overall Model 38](#_Toc197002039)

[B.5.2 – Sensitivity Analyses: ‘No Observation’ Model 40](#_Toc197002040)

[B.5.3 – Sensitivity Analyses: ‘Some Observation’ Model 42](#_Toc197002041)

[B.5.4 – Sensitivity Analyses: ‘Explicit Observation’ Model 44](#_Toc197002042)

[B.6 – Meta-Regressions 46](#_Toc197002043)

[B.7 – Pre-Registered Analyses 47](#_Toc197002044)

[C – Discussion 51](#_Toc197002045)

[C.1 – Distinguishing ‘Moral’ Licensing from ‘Other’ Licensing 51](#_Toc197002046)

[C.2 – How Moral Reputation can be Judged Across Domains 51](#_Toc197002047)

[C.3 – Is Moral Licensing an Effect of Observation or Study Location? 52](#_Toc197002048)

[C.4 – Results Discussion for Additional Methodological Moderators 53](#_Toc197002049)

[C.5 –Discussion: Domain (In)Consistency and Reputation Judgements in Moral Licensing 54](#_Toc197002050)

[C.6 – How do Different Manipulations Fit Within a Reputational Account of Moral Licensing? 55](#_Toc197002051)

[C.7 – Moral Licensing vs Moral Consistency 57](#_Toc197002052)

[C.8 – Licensing Mechanisms: Moral Credits vs Credentials 58](#_Toc197002053)

[D – PRISMA Checklist 59](#_Toc197002054)

[E – References 64](#_Toc197002055)

[E.1 – Studies Included in the Meta-Analysis 64](#_Toc197002056)

[E.2 – Supplement References 70](#_Toc197002057)

# A – Methods

## A.1 – Materials and Procedures

### A.1.1 – Coding: Observation and Ambiguity Scales

**Table S1**

1. *Observation Scale*

| Scale Point | Criteria |
| --- | --- |
| 1. No Observation | The participant is not observed or is unaware of observation during the study. For example, the study is being conducted online, or participants are explicitly told they are anonymous and have no reason to expect that they will be observed. |
| 1. Some/Unclear Observation | The study is unclear whether participants were fully aware that they were being observed. Participants might be suspicious that they were being observed. For example, participants fill out a survey in a cubicle with a researcher walking around. |
| 1. Explicit Observation | The participant is aware that their answers are being heard/seen by others or believes that they will be observed by one or more others. This should greatly affect a person’s response as their reputation is clearly at risk. |

1. *Ambiguity Scale^[[1]](#footnote-2)^*

| Scale Point | Criteria |
| --- | --- |
| 1. Little-to-no Ambiguity | Choice of action is highly diagnostic of one’s good vs. bad character. Almost all good people do the moral action, only bad people do the immoral action. There is only a single motivation for the “immoral” act, such that it is nearly |
| 1. Some Ambiguity | Choice of action is moderately diagnostic of one’s good vs. bad character, but with some exceptions. Good people usually do the “moral” action, but not always – there are some conditions when good people will do the “immoral” action. The “immoral” act is generally considered to be bad, although there is at least one potential excuse that could account for the “immoral” act under some circumstances. Someone could defend or justify their decision, but it would take some work, and listeners might not believe the justification. Examples: splitting money unfairly or donating to charity (one might have high needs oneself), willingness to volunteer in general (may have time constraints), support for the environment (though can be very ambiguous for some DVs) |
| 1. Moderate Ambiguity | Choice of action is partly diagnostic of one’s good vs. bad character, but there are many exceptions. Good people do the moral action more than bad people do, but there are many situations where good people would not do so and/or many good excuses for doing the “immoral” action. Seeing the immoral action would predispose an audience to think of the actor as “less good”, but one could easily convince them otherwise. Example: willingness to volunteer at a specific time and place when one might be unavailable, willingness to perform a type of help that one might not be able to perform (e.g., blood donations & whether one meets exclusion criteria) |
| 1. High Ambiguity | Choice of action is not very diagnostic of one’s good vs. bad character, there are too many exceptions. Many good people do the “immoral” action: there are many valid motives, reasons, and excuses for doing so. Although the “immoral” action may be seen as bad, there are enough valid reasons for doing so that it is difficult to assess the “true” motives of the participant. Someone could very easily defend or justify their decision, or the decision might not even need defending. Examples: which of two similar and equally qualified people do you hire, donating to a dubious charity or questionable recipient. |

### A.1.2 – Rationale for Moderator Analyses

Below we justify and describe the moderator analyses. For each moderator analysis, we computed a multi-level meta-analysis.

***Observation.*** People tend to behave more cooperatively when they are being observed, as it provides an opportunity to build a positive reputation. We proposed that this reputational motive underlies the moral licensing effect—predicting that licensing is more likely when participants are observed during the licensing phase, as they are actively establishing their public reputation.

Moral licensing is likely to occur once a good reputation has been established. After doing so, individuals may feel licensed to act less cooperatively, having earned "moral credits" in the eyes of others. Therefore, we predicted a stronger licensing effect when participants were observed during the licensing manipulation (e.g., by an experimenter, confederate, or peers), compared to when they were unobserved. Conversely, if licensing operates through self-image alone, the effect should be equally strong regardless of whether participants are observed or anonymous.

***Location of Study.*** In-person psychological studies typically involve more observation cues than online studies—such as the presence of an experimenter or being in a shared space with others—making participants feel more observed. Even when anonymity is maintained, lab settings can still influence prosocial behavior, suggesting participants perceive some level of observation (Barmettler et al., 2011). Based on this, we predicted smaller moral licensing effects in online studies, where reputational cues (e.g., visibility, physical presence) are limited. To test this, we coded study location as either online or in-person, with the latter including lab, field, and classroom settings.

***Moral Ambiguity of the Dependent Measure.*** We theorized that reputation influences the moral licensing effect: once individuals establish themselves as moral or good, they may feel permitted to act slightly less morally without damaging that reputation. However, this effect depends on how clearly the dependent measure signals moral behaviour. In other words, the size of the licensing effect is influenced by how interpretable the behaviour is to observers.

When a dependent measure is highly ambiguous, it offers little clear information for others to judge whether someone acted morally or immorally. In contrast, when a measure has a clearly ‘right’ or ‘wrong’ response—such as blatant moral transgressions (Effron & Monin, 2010)—it becomes easier for observers to evaluate the person's character. Based on this, we predicted stronger licensing effects in studies using morally ambiguous dependent measures.

***Publication Status.*** As found in prior meta-analyses, published studies had larger effects than unpublished studies (Blanken et al., 2015; Simbrunner & Schlegelmilch, 2017), likely due to the tendency for studies with significant or supportive findings to be published more frequently than null results (Ioannidis, 2005). To examine this pattern in our updated dataset, we tested whether publication status moderated the size of the moral licensing effect.

***Control Condition****.* Moral licensing studies often use either neutral or negative control conditions. Prior meta-analyses have shown mixed findings: one found no moderating effect of type of control condition (Blanken et al., 2015), while another reported that negative controls produced larger effects than neutral ones (Simbrunner & Schlegelmilch, 2017). Theoretically, larger differences are expected when using negative controls, potentially due to a licensing effect, a compensation effect, or a combination of both. When comparing initial immoral (negative) behaviours to moral (positive) ones, it's difficult to disentangle whether subsequent behaviour reflects moral licensing, compensation, or both.

Given this, we predicted a replication of the prior finding—larger effects when using negative controls compared to neutral ones. However, we expected a smaller effect in our sample due to differences in inclusion criteria: specifically, when studies included both control types, we only included the neutral comparison, whereas previous meta-analyses included both comparisons in their analyses.

***Participant Culture.*** Simbrunner and Schlegelmilch (2017) found that culture moderated the moral licensing effect, reporting stronger effects in North America compared to Western Europe, and a moral consistency effect in Southeast Asia. We aimed to replicate and expand on their findings by incorporating more recent studies conducted in additional cultural contexts, including Africa, Australia, and the United Kingdom. This analysis was both confirmatory and exploratory, as we sought to replicate previous results while also examining new cultural contexts without specific predictions about effect sizes.

***Domain Consistency.*** Licensing effects have been shown to influence not only outcomes within the same domain as the initial behavior but also across different domains (Mazar & Zhong, 2010; Miller & Effron, 2010). For instance, engaging in a pro-environmental act has been linked to increased likelihood of cheating (Mazar & Zhong, 2010). Two previous meta-analyses have found that domain consistency did not moderate the licensing effect (Blanken et al., 2015; Simbrunner & Schlegelmilch, 2017). Based on this, we predicted that domain consistency would not moderate the moral licensing effect in our analysis—though we note that this prediction was not preregistered.

***Domain of Study.*** We chose to conduct an exploratory analysis to determine if the effect size varied by domain. We chose to investigate study domain for two reasons (1) domain-specific behaviors are associated with different types of signals, which can influence the degree of reputational benefits others can attribute to the behavior or the degree of one’s prosocial self-perception, and (2) methodological differences across domains may impact the size of the observed effect. By aggregating effect sizes within domains, we aimed to better estimate the expected effect size for each domain—useful for informing future sample size planning—and to understand how domain-specific methods might shape the moral licensing effect.

After reviewing the literature, we categorized studies into moral domains, including cooperative behaviors (e.g., Barclay, 2014; Barque-Duran et al., 2016; Blanken et al., 2014), stealing or cheating (e.g., Clot et al., 2014; Mazar & Zhong, 2010), job hiring tasks involving race or gender (e.g., Cascio & Plant, 2015; Monin & Miller, 2001), and environmental tasks (e.g., Lacasse, 2019; Meijers et al., 2015). This analysis only included studies where both the licensing manipulation and dependent variable in the same domain. As a result, cheating tasks could not be included in this analysis because cheating was not manipulated in any study.

***Manipulation Type.*** Moral licensing has been studied using a variety of task types. To explore whether the type of manipulation influenced effect size, we categorized tasks into five groups: (i) **imagined behaviours**, where participants envisioned themselves performing an action (e.g., Clot et al., 2013, 2014, 2016), (ii) **writing primes**, in which participants described themselves using prosocial words (Sachdeva et al., 2009), (iii) **intended behaviours**, where participants reported future moral intentions (e.g., Effron & Conway, 2015), (iv) **recall of past behaviours** (e.g., Conway & Peetz, 2012), and (v) **behavioural tasks**, involving actual behaviour (e.g., Jones & Koenig, 2018; Mazar & Zhong, 2010).

This analysis expands on Blanken, van de Ven, and Zeelenberg’s (2015) moderation analysis, which compared trait-based versus action-based manipulations and found no significant differences in effect size. In contrast, Ferguson (2024) found that hypothetical tasks yielded licensing effects, while recall and behavioural tasks led to consistency effects—though that analysis combined findings from both foot-in-the-door and moral licensing studies.

To build on these mixed findings, we conducted an exploratory analysis focused solely on the moral licensing literature. As we had no specific a priori predictions regarding how manipulation type might influence effect size, this investigation was exploratory in nature.

***Monetary Dependent Variable.*** Payment (compared to unpaid conditions) has been shown to reduce effect sizes in various studies—for instance, leading to lower transfers in economic games (Amir et al., 2012; Bühren & Kundt, 2015; Rotella et al., 2019) and reduced risk-taking behavior (Irwin et al., 1992). This may be due to participants overstating preferences in hypothetical scenarios (List & Gallet, 2001) or adjusting responses for social desirability, also known as “presentation effects” (Camerer et al., 1999). Furthermore, monetary dependent measures often involve competing motivations, weighing financial incentives against prosocial goals.

Because of these mixed motives, we predicted that studies using real monetary outcomes would show a smaller moral licensing effect than those using hypothetical dependent measures, as the manipulation would likely have less influence on financially driven decisions.

### A.1.3 – Rationale for Inclusion Criteria: What is ‘Moral’?

Moral behaviors are subjective. They involve judgement of ‘morality’ from a perceiver (whether of oneself or by others), which varies across populations (Awad et al., 2020; Haidt & Joseph, 2004). To simplify this complex array of behaviors and judgements, we determined if a behavior was ‘moral’ for inclusion in this study based on two key criteria:

1. First, the licensing manipulation or dependent measure must fit within the definition of morality of leading theories of morality (detailed below): Morality-as-Cooperation theory (Curry, 2016) or Moral Foundations theory (Graham et al., 2013).
2. Secondly, it must be readily judged as moral by the majority of people within the relevant population studied. Because this meta-analytic effort is an empirical study looking at average behaviors, any behaviors that are questionably moral (i.e., whether some people might or might not consider it moral, or it might be viewed differently by different groups) would introduce confounding factors that could reduce the size of the effects studied. This would be particularly problematic in cases where ‘questionably moral’ manipulations or outcomes are not evenly distributed cross conditions (e.g., if more ‘questionably moral’ studies were conducted under unobserved conditions compared to observed observed). Thus, these studies were excluded from the present effort.

***Moral Theories: Morality-as-Cooperation & Moral Foundations Theories***

There are two leading theories about human morality, the morality-as-cooperation hypothesis and moral foundations theory (Curry, 2016; Graham et al., 2013). The Morality-as-Cooperation hypothesis suggests that morality is a collection of cultural and biological solutions that solve consistent and recurrent problems of human cooperation (Curry, 2016; Curry et al., 2019). Moral foundations theory, on the other hand, states that there are five distinct domains of morality: care/harm, fairness/cheating, loyalty/betrayal, authority/subversion, and sanctity/degradation. There is notable overlap between the two theories, where two of the five domains in moral foundations theory are related to cooperation: care/harm and fairness/cheating.

For all behaviors that met the definition of moral above, we then applied our second criteria about population-level perceptions of morality. If any could be considered questionably moral (i.e., hand washing, eating red meat), they were excluded from the present analysis.

### A.1.4 – Rationale for Exclusion: Other Sequential Moral Effects

We excluded additional bodies of literature on sequential moral behaviors for two reasons:

1. First, we focused on the moral licensing effect in our pre-registration, in which we specified search terms that were only related to moral licensing.
2. Because of the methodological differences among sequential moral behaviour effects (i.e., foot-in-the-door, door-in-the-face, moral cleansing, rebound effects), our theoretical predictions about effect sizes in these studies differ from those made for the moral licensing literature.

***Procedure and Theory Differences for Sequential Moral Behaviour Effects***

Moral licensing is one effect in a broader array of studies that examine sequential moral behaviors, where one (im)moral behavior sequentially precedes engaging in another (im)moral behavior. These include effects such as the *foot-in-the-door* (i.e., where a small request increases the chance of someone agreeing to a subsequent larger request), *rebound* (i.e., effects in the sustainability literature, where a new/better technology or behavior is introduced, which is followed by increase in sustainability behaviors), *moral cleansing* (i.e., when someone performs an immoral action, subsequently behaves more morally), and *door-in-the-face* (i.e., when someone declines a large request, they are more likely to accept a subsequent smaller request).

Although on the surface there are similarities between these literatures, these systematic differences in experimental methodologies would have likely produced systematic differences in the obtained effect sizes. ’Body of literature’ would have had to be included as a moderator for all variables, or a more powerful approach would be to conduct separate meta-analyses for both foot-in-the-door effects and rebound effects, which was beyond the scope of this effort.

1. **Valence of manipulation (Effects: Moral Cleansing; Door-in-the-Face).** Moral cleansing and door-in-the face effects both start with a *negative manipulation*, where the first request is either a negative moral action (i.e., moral cleansing) or declining a request (i.e., door-in-the-face), which results in an increase in moral behavior (i.e., being more moral or complying with a request) in a subsequent action. This is the opposite of the moral licensing effect, which has a *positive manipulation* followed by a decrease in moral behavior.
2. **Systematic variation in the ‘size’ of manipulation (Effect: Foot-in-the-Door).** Foot-in-the-door experiments are designed to study compliance effects. These methods always start with a small request from the participant, and is followed up with a larger request, which are not always in the moral domain (Burger, 1999). On the other hand, in the moral licensing literature, experiments are designed to study sequential *moral* behaviours, the “license” rarely involves a request from the participant (e.g., can be recall of past behaviour, writing primes, future intentions), and the size of the request is not systematically varied. Even if the differences appear to be small, we would make different predictions about the direction of effects in these bodies of literature if we take into account reputation-based theory. Specifically, because the first request is always small for the foot-in-the-door effect, participants would only establish a weak reputation as a good person. If they do not agree to the second request, which is larger and more costly to the participant, they would expose their agreement as a thin veil: they are only a good person as long as they don’t have to do much. So, denying the second request would expose them as a non-cooperator, which would have reputational consequences. Thus, we would predict that people would be *more cooperative* in the subsequent behaviours. On the other hand, when a good reputation is established and the second action is of similar or of lesser size, people can afford to be less cooperative and still maintain that reputation. Therefore, based on the relative magnitudes of the initial and subsequent tasks in foot-in-the-door versus moral licensing studies, we would expect a consistency effect (or rather people be more cooperative in the second instance) for the foot-in-the-door effect, whereas we predict a licensing effect in the moral licensing literature.
3. **Study Context & Design (Effect: Rebound Effect).** Rebound effects refer to an increase in energy consumption (or other sustainability behavior) following the introduction of a new/better technology or behaviour. These studies typically employ quasi-experimental or correlational designs, or use secondary sources of data (Sorrell, Dimitropoulous, & Sommerville, 2009). Additionally, unlike in the moral licensing literature, participants in rebound effects experiments typically do not self-license (i.e., they are not licensed by their own past behaviours, rather by others implementing a technological intervention which results in pro-environmental benefits). Because self-licensing is a key criterion for the current project, we excluded this body of literature.

##

### A.1.5 – Rationale for Exclusion: Boundary Conditions

Boundary conditions are, by definition, conditions where the effect of interest is no longer thought to occur (i.e., conditions where the effect does not apply, or becomes non-significant). These manipulations are chosen to intentionally attenuate or eliminate an effect, based on the theorized mechanism. Boundary conditions are important for testing the theory of the effect, to determine when, why, what, and who the effect in question applies to. Notably, when testing boundary conditions the prediction is that there will be no effect (or it will be reduced) rather than usual approach in psychology where the predictions that there will be an effect. In other words, the prediction is that there will be no difference between the experimental and control conditions because the manipulation is predicted to be ineffective in eliciting the effect .

Although testing boundary conditions is important for understanding when and why an effect happens, they should not be included in meta-analyses attempting to determine the true size of an effect. This is because they are testing different things – regular tests are attempting to find an effect, whereas boundary tests are attempting to find a null result. In meta-analysis, this is often referred to ‘comparing apples and oranges.’ If included in meta-analysis to estimate the size of a true effect, including boundary conditions should theoretically lead to an under-estimate of the true effect because the predicted effect size from these studies would be 0.

Given the purpose of testing boundary conditions is different than testing the existence of an effect, which could lead to an under-estimate of the true effect, we chose to exclude these from the present meta-analysis. Therefore, if a study included both boundary and non-boundary conditions, then we excluded the boundary conditions and included the rest. If a study only included a boundary condition, without a non-boundary condition, then we excluded it altogether.

### A.1.6 – List of Excluded Studies

Below is a list of studies excluded from the meta-analysis. For papers lacking the necessary statistics to compute effect sizes, we contacted the authors to request the missing data. If no response was received after two follow-up attempts or by the cutoff date (February 15, 2019), we estimated effect sizes when feasible (e.g., by evenly dividing the total sample size across conditions). If estimation was not possible, the study was excluded from the analysis.

**Table S2**

Excluded Study List and Rationales

| **Author** | **Study** | **Exclusion Rationale** |
| --- | --- | --- |
| Aquino et al., 2009 | 1 | There was no self-licensing; manipulations included reading the Ten Commandments and writing a story using morally laden terms. These do not fit the inclusion criteria that participants should license by indicating (a) intended ‘good’ or moral behavior, (b) performing a ‘good’ or moral action, or (c) report the recall of a past ‘good’ or moral behavior |
| Aquino et al., 2009 | 4 | Not enough information provided to calculate effect sizes (no group ns or standard deviations); no response from author |
| Barque-Duran et al., 2016 | 3 | Not enough information provided to calculate effect sizes; No response from authors |
| Barclay, Unpublished | 2 | Licensing ‘toughness’ is not included as a characteristic of morality according to moral foundations or morality-as-cooperation theories |
| Blanken et al., 2015 | 7, 7, 9, 9 (unpublished data from meta-analysis) | Not enough information to include study and authors did not respond to contact requests |
| Botchway, 2014 | all | Unable to access dissertation or contact author |
| Boyd, 2014 | 1 | Not enough information provided to calculate effect sizes for all comparisons |
| Bradley-Geist et al., 2010 | 3 | Not enough information provided to calculate effect sizes; No response from authors |
| Bradley-Geist et al., 2010 | 4 | The comparison between the two acquaintance conditions was not included because there was not enough information provided to calculate effect sizes |
| Bradley-Geist et al., 2010 | 5 | Dependent measure was how much participants the degree to which participants used emotion in descriptions of minorities. As emotionality is vague and not clearly associated with the major theories of morality, this study was excluded. Additionally, this is the sole study with this type of DV included in the reviewed moral licensing literature, which is problematic for inclusion in meta-analysis. |
| Brañas-Garza et al., 2013 | all | Authors used a repeated measures within-subjects design. To keep studies methodologically consistent, we excluded any studies that were not between-subjects. Additionally, there was also no self-licensing; participants played a series of economic games. Decisions in economic games are not clearly (im)moral and vary substantially across cultures (Nouri & Traum, 2013). |
| Braun & Gollwitzer, 2012 | all | Crime leniency for outgroup members is not clearly a moral behavior, according to either major theory of morality. That is, whether someone indicates high or low leniency for outgroup members does not necessarily reflect that the target is moral or immoral (is it more moral to be lenient, or more moral to not be lenient?). Thus, morality is difficult to establish with this DV. |
| Brewer et al., 2007 | all | Not clearly a moral licensing study. This paper investigated the effect of vaccination on disease-avoidance behaviors |
| Brown et al., 2011 | all | Licensing manipulation did not fit inclusion criteria.  In this study, participant rated the *likelihood of performing moral actions*. Likelihood judgments do not fit our manipulation inclusion criteria of it being: (a) an intended good or moral behaviour, (b) performing a good or moral behaviour, or (c) did not recall past moral behaviour. Thus, it does not fit inclusion criteria |
| Cain et al., 2005 | all | Estimation and making recommendations does not fit inclusion criteria that "The licensing manipulation must include participants indicating (a) intended ‘good’ or moral behaviour, (b) performing a ‘good’ or moral action, or (c) report the recall of a past ‘good’ or moral behaviour." Additionally, the licensing manipulation may be confounded with other variables, such as reputation and this study used a repeated-measure design (which were excluded in the present meta-analysis). |
| Cain et al., 2011 | all | Participants were told that the advisor had either been made aware of the study incentives or not. This is not a self-licensing manipulation, and therefore does not fit inclusion criteria that "The licensing manipulation must include participants indicating (a) intended ‘good’ or moral behaviour, (b) performing a ‘good’ or moral action, or (c) report the recall of a past ‘good’ or moral behaviour."  Additionally, manipulation may be confounded with other variables, such as reputation. |
| Carattini & Tavoni, 2016 | all | Licensing was not manipulated; does not fit inclusion criteria |
| Carrico et al., 2018 | all | Eating red meat is ‘questionably moral’, which varies according to in-groups (e.g., vegetarians/vegans my moralize against it, however, meat consumers may not). As this study includes a more general population, the dependent measure is not clearly moralized and does not fit inclusion criteria. |
| Chiou et al., 2011 | all | This paper investigated the effect of dietary supplements licensing health-risk behaviors. Studies are not in the moral domain; does not fit inclusion criteria |
| Choi et al., 2014 | 1 | Not enough information provided to calculate effect sizes |
| Choi et al., 2014 | 2 | Not enough information provided to calculate effect sizes |
| Choi et al., 2014 | 3 | Not enough information to accurately compute effect size |
| Conway & Peetz, 2012 | 1, 2 | Excluded 'distant past' manipulation because it is a boundary condition. The prediction was that a long-past license would not produce a license. As such, we would not expect a moral licensing effect in this study and was excluded. |
| Conway et al., (2014; unpublished) | 2, 3 | Moral manipulation was to describe the “behavior, values, lifestyle, and appearance” of superheroes, no self-licensing. |
| Cornelissen et al., 2007 | all | Social labeling does not fit inclusion criteria for self-licensing manipulation. Participants did not create the social labels, they were provided. |
| De Witt Huberts et al., 2012 | all | Studies investigate hedonic consumption, which are not clearly in the moral domain; does not fit inclusion criteria |
| Effron, 2014 | all | Estimation of other's perception of racism is not their own moral behaviors and would not create a self-license; does not fit inclusion criteria |
| Effron & Knowles, 2015 | all | There were no self-licensing manipulations |
| Effron & Monin, 2010 | all | There were no self-licensing manipulations |
| Effron et al., 2012 | 1, 4 | DVs of anticipated evaluations by others and of estimates of racist opportunities does not fit inclusion criteria. These are not self-licensing manipulations |
| Effron et al., 2013 | all | This paper investigates the effect of indulgence and unhealthy behaviours on counterfactual sins. Indulgence and health are not clearly in the moral domain; does not fit inclusion criteria |
| Ek, 2015 | all | Licensing manipulation is continuous, not categorical. This does not fit our inclusion criteria that licensing must be manipulated |
| Engelaar, 2017 | all | Same sample as de Jong (2017) |
| Eskine et al., 2012 | all | Studies investigated how moral events influenced ratings of beverages. DV is not in the moral domain; does not fit inclusion criteria |
| Falomir-Pichastor et al., 2018 | all | Preference for nationality is only tangentially related to morality, and there are no clear moral evaluation (is greater or lower preference associated with morality?). |
| Ferguson, 2019 | 1b, 2a, 2b | Excluded immoral intentions dependent measures, because it was a one-sided prediction. That is, if an effect was found it could be attributed to moral licensing, but if it was not found it may or may not be attributable to moral licensing. |
| Fishbach & Dhar, 2005 | all | Studies investigated of perceived goal progress on consumer choice. Studies are not in the moral domain; does not fit inclusion criteria |
| Frimer et al., 2015 | all | Describing personal goals is not a self-licensing behavior in the moral domain. This licensing manipulation does not fit our inclusion criteria |
|  | all | Licensing manipulation is a continuous measure and not experimentally manipulated; does not fit inclusion criteria |
| Garvey & Bolton, 2017 | all | Licensing was not manipulated; does not fit inclusion criteria |
| Geng et al., 2016 | 1a | Water consumption DV is not (clearly) in the moral domain; may have not been perceived as moral to participants. |
| Geng et al., 2016 | 3a/b | This experiment tested to reduce moral licensing effect and therefore there was no control condition (licensing condition was the control in this case) |
| Gholamzedehmir, 2015 | 1 | DVs lacked enough info to calculate ES |
| Gholamzedehmir, 2015 | 2 & 3 | Attitude and Moral norm DVs did not meet our inclusion criteria because participants did not (a) intend ‘good’ or moral behaviour, (b) perform a ‘good’ or moral action, or (c) report the recall of a past ‘good’ or moral behaviour. |
| Gholamzedehmir, 2015 | 4 | Not enough information provided to calculate effect sizes; No response from authors |
| Gholamzedehmir, 2015 | 5 | Did not manipulate licensing |
| Gholamzedehmir, 2015 | 1 | Not enough information provided to calculate effect sizes; no response from author |
| Gneezy et al., 2012 | all | Participants did not make donations themselves, therefore there was no self-licensing manipulation |
| Hayley & Zinkiewicz, 2013 | all | Not enough statistics to calculate effect sizes |
| Ho et al., 2016 | all | This study investigated how peer information influences production of negative externalities. There is no self-licensing; does not fit inclusion criteria |
| Jeong & Koo, 2015 | all | Purchasing a luxury/value brand of a produce is not in the moral domain |
| Jordan & Monin, 2008 | all | Self-rated morality is not a behavioural dependent variable |
| Jordan et al., 2011 | 1 | Self-rated morality is not a behavioural dependent variable |
| Jordan et al., 2011 | 1 | Indicating one's own morality is not a behavioural dependent measure |
| Karmarkar & Bollinger, 2015 | all | Purchasing organic products is not clearly in the moral domain (e.g., potentially health); does not meet inclusion criteria of being in a moral domain |
| Khan & Dhar, 2006 | all | Dependent measure (i.e. choice between conventional or luxury good) is not in the moral domain |
| Khan & Dhar, 2007 | all | Studies are not in the moral domain (e.g. consumerism, choice between conventional or luxury goods); does not fit inclusion criteria |
| Klotz & Bolino, 2013 | all | Model is not empirically tested |
| Koritzky, 2006 | all | Participants could act to benefit the other player in the first game (whatever that was), but this isn’t necessarily moral, it could be strategic, thus the manipulation might not have granted a license, but merely given participants a chance to make a strategic decision |
| Kouchaki, 2011 | all | There was no self-licensing (only vicarious); does not fit inclusion criteria |
| Lalot et al., 2018 | 1 | Telling participants that an article has either majority or minority support from a population is not a licensing manipulation. There is no opportunity for self-licensing |
| Lalot et al., 2018 | 3 | Licensing was not manipulated; does not fit inclusion criteria |
| Lanzini & Thøgersen, 2014 | all | There was no self-licensing; does not fit inclusion criteria |
| Leonard, 2012 | Conditions on food consumption | Conditions on food consumption are not in the moral domain; does not fit inclusion criteria |
| Li et al., 2017 | 2 | Not enough information provided to calculate effect sizes; No response from authors |
| Lin et al., 2016 | all – except for Study 2 follow up data | Licensing was not manipulated; does not fit inclusion criteria |
| Mann & Kawakami, 2012 | 1,2 | Excluded IAT, not a behavioural dependent measure |
| May & Irmak, 2014 | all | Investigated indulgence in eating, spending, and studying. Studies are not in the moral domain; does not fit inclusion criteria |
| Mazar & Zhong, 2010 | 1 | No licensing manipulation |
| Meijers et al., 2015 | 2.1 | Omitted because we included the published paper, not the one from the dissertation to avoid duplicates |
| Meijers et al., 2015 | 3.3 pre-test | Did not look at behavioural effects of being licensed |
| Meijers et al., 2015 | Chapters 4 and 5 | Did not manipulate licensing |
| Meijers et al., 2015 | 3.3 | Excluded DVs on identity relevance; not in the moral domain |
| Merritt et al., 2012 | 1, 2 | Not enough information provided to calculate effect sizes; No response from authors |
| Merritt et al., 2012 | 3 | Did not manipulate licensing |
| Merritt et al., 2012 | all – except for Study 2 follow up data | The licensing dependent variable was not preceded by good behavior. Rather, participants know they might have to behave questionably later and therefore strategically license. Thus, the licensing manipulation is confounded with strategy |
| Millet et al., 2022 | 2 | Multilevel statistics; unable to covert stats for inclusion in the meta-analysis |
| Mukhopadhyay & Johar, 2009 | all | Studies on consumer choice and temptation; not clearly in the moral domain; does not fit inclusion criteria |
| Mukhopadhyay et al., 2008 | all | Studies on consumer choice and temptation; not clearly in the moral domain; does not fit inclusion criteria |
| Nagel, 2014 | all | Not enough information provided to calculate effect sizes; No response from authors |
| Ong et al., 2014 | all | Participants do not perform good behaviours themselves, therefore there is no licensing. |
| Panzone et al., 2013 |  | Licensing was not manipulated; does not fit inclusion criteria |
| Polman et al., 2013 | all | There is no self-licensing; does not fit inclusion criteria |
| Raska, 2010 | 1 to 3, 4b, 5 | Consumption-based dependent measure (luxury vs non-luxury goods or good/bad food); not in the moral domain |
| Raska, 2010 | 4a | The licensing manipulation does not include participants indicating (a) intended ‘good’ or moral behaviour, (b) performing a ‘good’ or moral action, or (c) report the recall of a past ‘good’ or moral behaviour. |
| Robitaille, 2014 | 1.3 | Not enough information to accurately compute effect size |
| Rotella, 2015 | Chap 2, Study 1, DV: Watching Others | Manipulation for "watching others" condition did not fit inclusion criteria |
| Schwabe et al., 2018 | 1 | Organic coffee may not be considered 'moral' by all, therefore this study was excluded |
| Schwabe et al., 2018 | 1-4 | No self-licensing; does not fit inclusion criteria |
| Seçilmiş, 2018 | all | Not enough information provided to calculate effect sizes (no group ns or standard deviations); no response from author |
| Shaw et al., 2011 | all | Licensing manipulation is continuous, not categorical; does not fit inclusion criteria that licensing was manipulated |
| SimanTov-Nachlieli et al., 2018 | all | There was no self-licensing; does not fit inclusion criteria |
| Simon & O’Brien, 2015 | all | No self-licensing; does not fit inclusion criteria |
| Steinhorst & Klöckner, 2018 | all | Licensing manipulation is continuous, not categorical; does not fit inclusion criteria that licensing was manipulated |
| Szekeres et al., unpublished | 1 | Undisclosed condition does not fit our inclusion criteria. There was no opportunity to self-license |
| Tiefenbeck et al., 2013 | all | No self-licensing; does not fit inclusion criteria |
| Ule et al., 2009 |  | DV confounded with strategy |
| Zhang & Hunt, 2008 | all | Rebound effects are not moral licensing; does not fit inclusion criteria |
| Zhong et al., 2010 | all | Within-participant design; Not enough information provided to calculate effect size |

### A.1.7 – Rationales for Treatment of Experimental Moderators

**Table S3**

Rationale of Treatment of Experimental Moderators in Moral Licensing Studies

| **Authors** | **Study** | **Justification for Moderators**  **(i.e. Inclusion/Exclusion justifications)** |
| --- | --- | --- |
| Barque-Duran et al., 2016 | 2 | Only included: Stage 1; outcome-based mindset.  Authors predicted a moral licensing in the outcome-based mindset, and moral consistency for rules-based mind-set. Thus, because the authors predicted the rules-based mindset would not create licensing, we only included the outcome-based mindset in the present analysis. |
| Barclay, 2014 | 1 | Included: Compared neutral control vs normal licensing, and neutral control vs less licensing |
| Bradley-Geist et al., 2010 | 1, 2 | Included both the ‘forced to write’ and ‘choice to write’ about minorities conditions.  Although the authors predicted a larger effect in the choice condition, they did not predict that there would be no moral licensing effect in the forced condition. The forced condition still provides participants the opportunity to establish credits/credentials, and as a result it was included in our meta-analysis. |
| Conway & Peetz, 2012 | 1, 2 | The authors predicted that moral licensing would not occur when participants would remember an action in the distant past. This may have reputation-based consequences because being slightly good a year ago would be unlikely to influence one’s present reputation. Therefore, we excluded the distant past condition from our analyses. |
| Conway & Peetz, 2012 | 3 | This study examined whether participants considered the concrete steps involved in performing moral/immoral actions (action condition) or possessing moral traits (identity condition).  Authors predicted licensing in the action condition, but not identity condition. Moreover, for a reputation-based interpretation of moral licensing, we would only expect a licensing in the action condition because actions carry reputation-based information, whereas simply stating how those traits would relate to their personality would not (or to a very small degree). Thus, we only included the action condition. |
| Conway et al., (2014; unpublished) | 1, 4 | Combined donations to charities (i.e., library donations to school, university, and prison; Study 1) and willingness to help charities (i.e., willingness to help school and prison libraries). We combined them because our coding for observation and ambiguity did not vary according to the specific charity. Additionally, these items were measured within-subjects and all were prosocial (charitable donations). |
| Cornelissen et al., 2013 | 1, 2, 3 | Only included outcome-based mindset.  Authors predicted a moral licensing in the outcome-based mindset, and moral consistency for rules-based mind-set. Additionally, not following the rules in front of an audience might have reputational costs, leading to a consistency effect. Thus, we only included the outcome-based mindset in the present analysis. |
| Engelaar, 2017 | 1 | Not enough information to include neutral control, so used negative control. |
| Ferguson, 2019 | 1b, 2a, 2b | Excluded immoral intentions dependent measure because it was a one-sided prediction. That is, if an effect was found it could be attributed to moral licensing, but if it was not found it may or may not be attributable to moral licensing. |
| Geng et al., 2016 | 2 | Although the researchers predicted that there would be a larger licensing effect in the progress condition than the commitment condition, there were not enough stats provided to include only the commitment condition. Statistics were provided for the overall effect. Thus, we used the combined (progress and commitment conditions) stats. |
| Greene & Low, 2014 | 1 | Included both the ‘private’ and ‘public’ conditions. Although the authors predicted a smaller effect in the public condition, this is contrary to the predictions made in the present analysis. Additionally, the public and private conditions were only imagined, and not really public/private. |
| Lacasse, 2019 | 1 | No a priori prediction for differences in the behavior adoption with message condition or with self-report calendar condition; thus, both were included in this analysis. |
| Lalot et al., 2018 | 2 | Excluded minority support condition because authors predicted it would produce a consistency effect. Also, when pro-environmental values are not supported by the majority of population (i.e 12% in the minority condition), there would be no reputational advantage to being pro-environmental. |
| Mann & Kawakami, 2012 | 3 | Compared progress feedback condition (where authors predicted a moral licensing effect) to no feedback condition instead of the negative feedback condition, as per inclusion criteria of using a neutral over a negative control when both were manipulated. |
| Noblet & McCoy, 2018 | 1 | We compared the Dose (green license) to No Dose (no green license), where the Does-Green and Dose-Not-Green conditions were combined (both provided opportunities for moral licenses). |
| Robitaille, 2014 | 1.1, 1.2 | Participants either put their recalled behaviors in an envelope (closure condition) or not (no closure condition) before the experimenter collected them.  Although the author predicted no licensing in the closure condition, we included both conditions because (a) the act of enclosing reports of past moral behaviors should not impact moral licensing if self-image is the mechanism, and (b) an alternative way to interpret this manipulation is that the experimenter can see responses in the no-closure condition, but not in the closure condition, thus it is a reputation-based manipulation. |
| Rotella, 2015 | Chap 2, Study 1 | Excluded condition where participants were ‘watching others’ because a license was not established for themselves. |
| Schwabe et al., 2018 | 1, 2, 3 | Authors predicted a moral consistency effect for prevention-focused manipulation. This manipulation had participants “describe two of their duties and obligations and wrote a short paragraph about them” and think about losses before recalling a past moral behaviour. If moral licensing was a reputation-based phenomenon, focusing on a loss or obligation might reduce the “licence” one gets from being a good person, because observers would not attribute a loss as a good action and obligations are not actions that one does because they are a good or moral person, but because they have to. Therefore, we would not expect licensing in these conditions and excluded conditions with these manipulations. |
| Susewind & Hoelzl, 2014 | 1, 2 | The researchers predicted: a) a moral consistency effect among participants who were primed with their commitment towards a moral goal (commitment condition); b) a moral licensing effect among participants who were primed with their progress towards a moral goal (progress condition). Therefore, we excluded the commitment condition from both studies. See further justification in the discussion section of the manuscript.  We also included the control condition in Study 1, because licensing was predicted to be found here. |
| Szekeres et al., unpublished | 1 | Participants read an empathy-inducing article, then were given the chance to disclose their feelings/empathy (disclosure condition), which can be interpreted as a moral self-license. However, when participants were not given the opportunity to disclose, there was no license. Thus, the no-disclosure condition was excluded. |
| Urban et al., 2019 | 2, 3 | Moral licensing was not predicted for the conventional condition because no license was established. Therefore, we excluded this condition, and compared the green condition to the control condition. |

### A.1.8 – Differences Among Meta-Analyses on Moral Licensing

**Table S4**

*Comparisons Between Meta-Analyses on Moral Licensing*

|  | **Meta-Analysis** | | | | |
| --- | --- | --- | --- | --- | --- |
|  | **Blanken et al., 2015** | **Simbrunner &**  **Schlegelmilch, 2017** | **Kuper & Bott, 2019** | **Ferguson et al., 2024** | **This Paper** |
| **Purpose** | Estimate the overall moral licensing effect and some moderators. | Replicate Blanken et al. (2015) and examine culture as moderator. | Test whether evidence for moral licensing was inflated by publication bias. | Broadened scope to sequential moral behavior (SMB), including licensing, consistency, foot-in-the-door, and door-in-the-face effects; aimed to identify conditions predicting each. | Test whether observation moderates moral licensing; test ambiguity and other moderators. |
| **Included Years** | Up to 2014 | Up to 2016 | Same studies as Blanken & Simbrunner (rechecked). | Up to 2022 | Up to 2022 |
| **Search Strategy** | Searched Web of Science, Google Scholar; journal RSS feeds; calls on SPSP, SJDM, ASPO forums and conferences​ | Built on Blanken et al.'s database; updated search in ProQuest, EBSCO, and Web of Science; included more studies without strict moral licensing labels​ | Re-analyzed Simbrunner's dataset with corrections (outlined in article); no new search​. | Comprehensive multi-database search (PsycInfo; Proquest, Medline, Web of Science); separate strings for moral balancing, foot-in-the-door (FITD), environmental spillover; searched reference lists; calls for unpublished data​ | Combined Blanken et al., Simbrunner et al., Ferguson et al. studies (up to 2022); independent search of 2013–2018 studies on Google Scholar, PsycInfo, Web of Science; calls for unpublished data at major conferences |
| **Inclusion Criteria** | (1) Behavior in a moral domain;  (2) Independent variable must involve actual/intended moral action or recall;  (3) Dependent variable must be behavior (not just attitudes);  (4) Adequate statistics​. | Broader: Included some studies outside Blanken’s strict criteria; excluded papers on prospective moral licensing | Same as Simbrunner but corrected coding errors and removed ineligible studies (e.g., vicarious licensing, non-moral tasks); aggregated multiple outcomes within same studies | (1) Individual participants; (2) Experimental design with random allocation; (3) Positive/negative moral manipulations (own behavior, not vicarious); (4) Control group included.  Included effects: moral balancing/licensing, moral consistency, door-in-the-face, foot-in-the-door | (1) Moral domain behaviour;  (2) Self-licensing manipulation;  (3) Experimental design;  (4) Adequate statistics​ |
| **Tested Moderators** | Publication status (published/unpublished)​;  Licensing manipulation (traits/actions);  Dependent variable (behaviou/hypothetical);  Domain consistency (same/different) | Culture (North America, Europe, Southeast Asia); Type of control condition (negative/neutral); Type of decision (self-related/society-related); Publication status (published/unpublished) | Culture moderator (US/Europe);  Control condition (Neutral/Negative) | Manipulation type (behaviour, recall, hypothetical); domain-consistency (same/different); target behavior valence (positive/negative) | Observation, study location, ambiguity of dependent variable, control condition, moral domain, domain consistency, monetary incentives, culture​, publication bias |
| **Analysis strategy** | Random-effects meta-analysis. | Random-effects meta-analysis; classical fail-safe N to address publication bias. | Random effects meta-analysis; then corrected with PET-PEESE and 3-PSM for publication bias​ | Random effects meta-analysis with robust variance estimation; corrected with PET-PEESE and 3-PSM for publication bias | Multilevel meta-analysis; robust Bayesian meta-analysis​, with sensitivity analyses. Robustness checks. |
| **Key findings** | Found a small-to-moderate overall moral licensing effect (Cohen’s d = 0.31). Only moderator identified was publication status: published studies had larger effects​. No effect of manipulation type, dependent variable, domain consistency or control condition. | Found a similar overall effect (Cohen’s d = 0.32). Identified culture as a significant moderator: moral licensing stronger in Western samples, reversed or absent in Southeast Asian samples. Type of control condition moderated the effect, with smaller effects for neutral controls. | After correcting for publication bias, found smaller/ null effects.  (PET-PEESE: d = –0.05; 3-PSM: d = 0.18). Concluded moral licensing effects were likely inflated​. Control condition moderated moral licensing; smaller effects with neutral controls compared to negative controls. | Broader analysis of sequential moral behavior (SMB). Found small moral licensing effect (g = 0.11), moral licensing mainly occurs for negative target behaviours; positive consistency in prosocial foot-in-the-door (FITD) studies. Effects small and sensitive to file-drawer bias | Found strong support for a social moral licensing mechanism: larger licensing effects when participants were observed. No moderation by ambiguity. Bayesian meta-analysis confirmed these patterns​ |
| **Effect Size** | Cohen’s *d* | Cohen’s *d* | Cohen’s *d* | Hedge’s *g* | Hedge’s *g* |
| **Other methods differences** | Included boundary conditions and vicarious licensing manipulations. | Broader but inconsistent coding; some included studies were misclassified or duplicates. | Did not collect new studies; focused on testing bias in previous datasets with better methods. | Expanded scope: included both moral licensing and positive consistency effects; not limited to prior self-labeling; computed correlated effects and hierarchical effects models. | Theory-driven criteria; excluded vicarious licensing, non-moral outcomes, non-boundary conditions; multi-level models correct for dependencies;  pre-registered. |

## A.2 – Analytic Decisions

### A.2.1 – Deviations From the Pre-Registration

Here we list the changes made during the research process that were different than the original pre-registration. All changes were made to improve the quality of the science from theoretical, methodological, and analytical perspectives.

**Hypotheses.** The hypotheses did not differ from the pre-registration.

**Inclusion Criteria and Decisions.**

*Pre-Registered Decisions.* These pre-registered decisions differed from previous meta-analyses (Blanken et al., 2015; Kuper & Bott, 2019; Simbrunner & Schlegelmilch, 2017).

1. Our inclusion criteria specified that both the (i) licensing manipulation and (ii) the dependent measures must be moral.
2. The licensing manipulation must include participants indicating (a) intended ‘good’ or moral behaviour, (b) performing a ‘good’ or moral action, or (c) report the recall of a past ‘good’ or moral behaviour.
3. In cases where there are two different moral licensing conditions (e.g., A and B) and a single control group (C), we report separate effect sizes for each licensing condition.
4. When studies included both a negative and a neutral control condition, we only include neutral control, omitting the data from the negative control. Prior meta-analyses included both conditions (Blanken, van de Ven, & Zeelenberg, 2015; Simbrunner, & Schlegelmilch, 2017).
5. When studies had multiple dependent measures that fit our criteria, we first assessed if they received the same codes across all moderator analyses. If yes, combined the variables. If not, included them as separate effect sizes.
6. We conducted literature searches in Google Scholar, PsycInfo, and Web of Science from 2013 (stopping data from prior meta-analysis efforts) and the pre-registered cut-off data, June 30 2018.

*Other Decisions (Not Pre-Registered).*

1. Boundary conditions were excluded (justified above, *A.1.5*).
2. We further updated our analysis following the publication of Ferguson (2024), which included studies until July 2022. Studies identified in this paper were assessed against our inclusion criteria. All moral licensing studies not assessed for the present effort were examined for inclusion.

**Analysis Strategy.** This approach differs significantly from our preregistration, as we adopted more robust and current methods. Initially, we planned to use different software (ESCI), assess bias with a funnel plot, and run standard (non-multilevel) models. Below, we explain the analysis we conducted and our rationale for deviating from the original plan.

1. **Software.** We analyzed all data in R, a more powerful and flexible software for meta-analysis. This allowed us to include a larger number of effect sizes (ESCI was limited to 20) and use more robust and replicable methods.
2. **Multilevel models.** We did not pre-register the use of multilevel models. However, many effects came from the same study or paper, meaning they weren’t independent—sharing methods, authorship, and likely similar outcomes. Since meta-analyses assume data independence, multilevel modeling was the most suitable approach for our data structure. Additionally, we used cluster-robust variance estimation to address correlated and hierarchical effects. This method, not included in our preregistration, is a recent advancement (Pustejovsky & Tipton, 2022) that wasn’t available at the time.
3. **Bias correction***.* We initially pre-registered the use of funnel plots to assess publication bias, but this approach is not compatible with multilevel data. Additionally, each publication bias correction method has its own strengths and weaknesses and performs variably depending on the context (Carter et al., 2019). To address this, we used Bayesian Robust Meta-Analysis, which applies 36 different bias correction models and averages them to estimate the probability of an effect. This method offers several advantages: it avoids over-reliance on any single correction technique, estimates the likelihood of bias in the dataset, and calculates the probability that a true effect exists given the data and potential biases. As such, it serves as a robustness check for our multilevel analysis while also evaluating bias.

**Specified Tests.** We followed our pre-registered analysis plan for the observation and ambiguity variables, which included comparing the extremes of the coding scale and overall analyses, with all subcategories. For observation, we chose to report the full moderation analysis in-text, as both the full model and the comparison of only the extreme categories (i.e., no observation vs. explicit observation) yielded significant results. While we initially pre-registered the extreme-category comparison as the strongest theoretical test, reporting the full model was appropriate given that all results aligned. The only deviation from the pre-registration was our use of multilevel models to account for dependencies among effect sizes in these analyses.

We did not examine interaction between observation and ambiguity because there were too few studies included per cell to meaningfully analyze.

### A.2.2 – Assessing Publication Bias

To our knowledge, there are currently no statistical tests available to assess publication bias in multilevel models. Although robust Bayesian meta-analysis does not account for data non-independence, it evaluates publication bias by fitting 36 different models and estimating the likelihood of bias. While these analyses were not pre-registered, they represent the most appropriate and up-to-date methods given the structure of our data.

In our full analysis—including all effect sizes—this method indicated extremely strong evidence of bias (BF = 1.90 × 10⁹). This is consistent with previous meta-analyses on moral licensing, which have consistently found evidence of publication bias (Blanken et al., 2015; Kuper & Bott, 2019; Simbrunner & Schlegelmilch, 2017). Given that unpublished studies generally have smaller effects than published ones, the effect size estimates obtained from the multi-level models are likely over-estimated. We recommend interpreting the lower confidence intervals of the estimates, or the estimates obtained from the Bayesian analyses.

### A.2.3 – Rationale for Multilevel Models

**Model Fit Comparison (multi-level v standard).** To determine whether a multilevel model was warranted, we compared model fits between two-level (i.e., standard meta-analysis) and three-level (multilevel meta-analysis) models.

Full (three-level) model had a better fit, relative to the two-level alternative. The Akaike Information Criterion (three-level AIC = 139.7, two-level AIC = 158.0) and Bayesian Information Criterion (three-level BIC = 148.8, two-level BIC = 164.0) are lower for this model, which indicates favorable performance. The likelihood ratio test (LRT) comparing both models is significant (χ^2^ = 20.29, p < .001) and points in the same direction.

**Nesting Structure.** We assume that individual effect sizes (level 2; defined by es_id) are nested within papers (or research efforts; many include multiple individual studies) (level 3; defined by author; see Table S5).

### A.2.4 – Rationale for Cluster-Robust Variance Estimate (CHE)

The Correlated and Hierarchical Effects (CHE) model groups multiple effect sizes into clusters based on shared features (e.g., same study, research group, or cultural context), while accounting for correlated sampling errors within clusters—such as when multiple measurements are taken from the same sample (Pustejovsky & Tipton, 2021; see Table S3).

In our analysis, clusters were defined at the study level, assuming that multiple effect sizes from the same study were correlated. This included cases where studies reported multiple dependent variables or conditions, producing related effect sizes. To address this, we used cluster-robust variance estimation.

***CHE Sensitivity Analysis***

The CHE model assumes that effect sizes within clusters are correlated, and that this correlation is consistent both within and across studies. Since the true correlation is unknown, we conducted a sensitivity analysis using small (g = 0.2), medium (g = 0.5), and large (g = 0.8) assumed correlations to assess their impact on the overall moral licensing effect. The results showed minimal variation (small: *g =* 0.1770; medium: *g =* 0.1777; large: *g =* 0.1787; all *p*s < .001), thus we continued analyses assuming a moderate correlation (i.e., 0.5) among effect sizes.

**Table S5**

*Dependencies in the Frequentist Analyses using Multilevel Models and Cluster-Robust Variance Estimation*

| **Levels Included** | **Level Definitions and Dependencies** |
| --- | --- |
| Level 1 | Individual participants |
| Level 2 | Individual effect sizes (pooled; es_id; this is standard non-multilevel meta-analyses) |
| Level 3 | Paper (accounts for dependencies between effects within a research effort, such as multi-study papers; paper_id) |
| CHE | Accounts for dependencies between effect sizes from the same study (i.e., some studies had multiple dependent variables or multiple licensing conditions, producing multiple related effect sizes; study_id) |

### A.2.5 – Rationale against using RoBMA moderation analyses

A moderated robust Bayesian meta-analysis method was recently developed (Bartoš et al., 2023). However, this approach assumes that publication bias operates uniformly across all conditions—an assumption that does not hold in our dataset. Our RoBMA results show substantially greater evidence of publication bias in the *no observation* condition compared to the *explicit observation* condition. Theoretically, this is also expected, as conditions producing smaller effects are more susceptible to publication bias. Therefore, we cannot justify using an analysis that assumes equal bias across conditions, as this would violate key assumptions of the method.

### A.2.6 – Formulas for Effect Size Calculation

We will be using standardized mean differences. Cohen’s *d* based on means and standard deviations will be calculates using the following formulas:


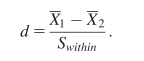


Where *X_1_* and *X*_2_ are the sample means in the two groups and *S_within_* is the within-groups pooled standard deviation:


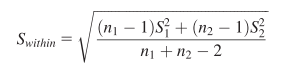


Here *n_1_* and *n_2_* are the sample sizes in the two groups, while *S_1_* and *S*_2_ are the standard deviations from the two groups. The variance of *d* will be calculated as:


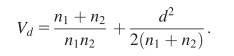


Where the square root of *V_d_* is the standard error of *d*:


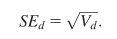


Bias will be corrected for using Hedges’ *g*, presented in the following formulas where *J* is the correction factor and *df* are the degrees of freedom (i.e., n1 + n2 – 2):


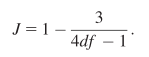


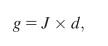


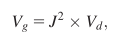


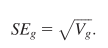


Cohen’s *d* based on *t* values will be calculated with the following formula, where *t* is the *t* statistic from the *t*-test and *n* is the overall sample size (Rosenthal & Rosnow, 1991). Then the variance of d will be calculated using the formula above for and the bias corrections (above) will be applied.


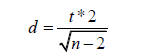


Repeated Measures Effect Size and Standard Deviations (Cumming, 2012)


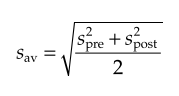


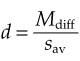


Sources: Borenstein, 2009; Rosnow, 1991

# B – Results

## B.1 – Frequentist Analyses: Neutral Controls Only

These analyses exclude donut designs, which compare experimental effects with negative rather than neutral controls, retaining only effects compared to neutral controls.

**Table S6**

*Moderator and Subgroup Analyses of the Moral Licensing Effect With Neutral Controls Only, using Multi-Level Meta-Analysis.*

|  | | **Estimates from multi-level models** | | **Descriptives** | |
| --- | --- | --- | --- | --- | --- |
| **Moderator** | | **Moderator test (grey)**  **Effect size (*g*) [95CI]** | **Test of effect size**  **(vs null model)** | **k** | **N** |
| **Overall Moral Licensing Effect** | | **0.15 [0.05, 0.24]** | ***t*(114) = 2.92, *p* = .004** | **114** | **14,404** |
| Observation*** | | *F*(2, 83) = 9.87, *p* < .001 |  | 111 | 12,346 |
|  | No observation | 0.06 [-0.03, 0.22] | *t*(83) = 1.27, *p* = .128 | 49 | 9,538 |
|  | Some observation | **0.21** [0.07, 0.34] | *t*(83) = 2.97, *p* = .004 | 29 | 2,410 |
|  | Explicit observation | **0.72** [0.43, 1.01] | *t*(83) = 4.98, *p* < .001 | 8 | 398 |
| Ambiguity | | *F*(3, 100) = 2.01, *p* = .118 |  | 137 | 13,482 |
|  | Little-to-no | **0.28** [0.09, 0.48] | *t*(100) = 2.86, *p* = .005 | 14 | 1,610 |
|  | Some | 0.07 [-0.04, 0.18] | *t*(100) = 1.32, *p* = .188 | 54 | 8.565 |
|  | Moderate | 0.13 [-0.08, 0.34] | *t*(100) = 1.20, *p* = .234 | 13 | 1.567 |
|  | High | **0.27** [0.09, 0.44] | *t*(100) = 2.99, *p* = .004 | 23 | 1,770 |
| Study location† | | *F*(1, 84) = 3.71, *p* = .058 |  | 112 | 12,346 |
|  | In-person | **0.25** [0.12, 0.38] | *t*(84) = 3.88, *p* < .001 | 48 | 4,915 |
|  | Online | 0.07 [-0.06, 0.21] | *t*(84) = 1.13, *p* = .264 | 38 | 7,431 |
| Publication status*** | | *F*(1, 113) = 10.94, *p* = .001 |  | 115 | 14,404 |
|  | Unpublished | -0.05 [-0.19, 0.10] | *t*(113) = -0.63, *p* = .532 | 56 | 5,462 |
|  | Published | **0.26** [0.15, 0.38] | *t*(113) = 4.50, *p* < .001 | 59 | 8,942 |
| Same vs different domain ^a^ | | *F*(1, 102) = 3.89, *p* = .051 |  | 104 | 13,512 |
|  | Same | **0.11** [0.00, 0.21] | *t*(102) = 2.04, *p* = .044 | 77 | 10,990 |
|  | Different | **0.30** [0.12, 0.47] | *t*(102) = 3.42, *p* < .001 | 27 | 2,522 |
| Across domains | | *F*(2, 74) = 0.34, *p* = .709 |  | 98 | 17,285 |
|  | Cooperation | 0.09 [-0.05, 0.23] | *t*(74) = 1.30, *p* = .197 | 52 | 6,684 |
|  | Environmental | 0.10 [-0.22, 0.42] | *t*(74) = 0.61, *p* = .544 | 6 | 2,770 |
|  | Discrimination | 0.19 [-0.03, 0.41] | *t*(74) = 1.77, *p* = .081 | 19 | 1,536 |
| Manipulation type | | *F*(4, 81) = 1.74, *p* = .149 |  | 112 | 17,223 |
|  | Imagined | 0.11 [-0.12, 0.34] | *t*(81) = 0.93, *p* = .356 | 16 | 1,582 |
|  | Writing Prime | 0.04 [-0.14, 0.22] | *t*(81) = 0.43, *p* = .668 | 21 | 4,223 |
|  | Intended Behaviors | **0.52** [0.11, 0.93] | *t*(81) = 2.53, *p* = .014 | 5 | 553 |
|  | Recall | 0.13 [-0.06, 0.31] | *t*(81) = 1.34, *p* = .184 | 20 | 3,277 |
|  | Behaviors | **0.27** [0.11, 0.43] | *t*(81) = 3.27, *p* = .002 | 24 | 2,711 |
| Participant culture*** | | *F*(4,107) = 7.45, *p* < .001 |  | 112 | 12,689 |
|  | North America | **0.21** [0.10, 0.33] | *t*(107) = 3.71, *p* < .001 | 65 | 8,184 |
|  | Europe | 0.10 [-0.07, 0.28] | *t*(107) = 1.19, *p* = .235 | 37 | 3,149 |
|  | United Kingdom | -0.45 [-0.99, 0.09] | *t*(107) = -1.65, *p* = .102 | 3 | 334 |
|  | Asia | **-0.58** [-0.90, -0.26] | *t*(107) = -3.63, *p* < .001 | 5 | 555 |
|  | Africa | 0.30 [-0.18, 0.79] | *t*(107) = 1.24, *p* = .219 | 2 | 467 |
| Monetary DV | | *F*(1,73) = 0.88, *p* = .352 |  | 75 | 10,726 |
|  | Hypothetical | **0.19** [0.07, 0.31] | *t*(73)= 3.12, *p* =.003 | 57 | 7,907 |
|  | Monetary | 0.09 [-0.11, 0.29] | *t*(73) = 0.89, *p* = .378 | 18 | 2,819 |

*Notes.* * indicates significance at *p* < .05, ** at *p* < .01, and ***** at *p* < .001*.* ***†*** indicated marginal significance (*p* < .06). Moderator test results and effect size estimates (with 95% confidence intervals) are presented under the ‘moderator test’ column, while tests determining if the subgroup is significantly different than zero are presented under ‘effect size tests’. All effect sizes are presented as Hedge’s *g*.

^a^ This analysis only contained studies in which the IV and DV were in the same domain

## B.2 – Frequentist Analyses: Excluding Studies with Change in Observation

To confirm that participants believed they were observed in both the moral licensing manipulation and the dependent measure, we excluded studies in which there was a change in observation (e.g., participants were more or less observed during the licensing manipulation compared to the dependent variable). Although this analysis was underpowered (*k*s: 63 in *no observation*, 24 in *some observation*, 3 in *explicit observation*), it serves as a confirmation check for the pattern of results obtained in the overall analysis.

Observation moderated the moral licensing effect size, *F*(2, 87) = 6.66, *p* = .002. The smallest effect was in the *no observation* condition (Hedge’s *g* = 0.12, 95CI[0.03, 0.21], *t*(87) *=* 2.76, *p* = .007), followed by the *some observation* condition (Hedge’s *g* = 0.40, 95CI[0.25, 0.55], *t*(87) *=* 5.27, *p* < .001), and the largest effect in the *explicit observation* condition (Hedge’s *g* = 0.73, 95CI[0.17, 1.29], *t*(87) *=* 2.59, *p* = .011). The effect was significantly larger in the *explicit observation* condition compared to *no observation*, *t*(87) = 2.12, *p* = .037, but did not differ from the *some observation* condition, *t*(87) = 1.12, *p* = .264. The effect was smaller *under no observation*, compared to *some observation*, *t*(87) = 3.13, *p* = .002. With only three studies included in the *explicit observation* condition, these results should be interpreted with caution. Notably, however, the results from this analysis are consistent with the overall effect obtained.

## B.3 – Frequentist Analyses: With Change in Observation

In exploratory analyses, we examined if a change in observation from the manipulation to DV (e.g., observed in manipulation to unobserved in DV, or vice versa), impacted the size of the moral licensing effect, using multilevel models.

Overall, change in observation did not moderate the moral licensing effect *F*(2, 110) = 2.37, *p* = .098. Interestingly, the largest effect was when there was no change in observation between the moral licensing manipulation and the DV, Hedge’s *g* = 0.24, 95CI[0.14, 0.33]*, t*(110) = 4.96, *p* < .001. Moral licensing effect estimates were no different from zero when participants were less observed during the moral licensing manipulation compared to the DV, Hedge’s *g* = 0.04, 95CI[-0.18, 0.26]*, t*(110) = 0.34, *p* = .734, and when participants were more observed during the moral licensing manipulation compared to the DV, Hedge’s *g* = 0.12, 95CI[-0.07, 0.31]*, t*(110) = 1.21, *p* = .229. Differences between conditions did not reach statistical significance, although we will note the low number of studies in some conditions (no change in observation: k = 90; more observed during the DV: k = 8, less observed during the DV: k =15).

These results are consistent with moral licensing theory using a reputation-based mechanism, we would expect a moral licensing effect when participants have had the opportunity to establish a reputation to someone, then they can act slightly less morally and still maintain that reputation. Notably if you did not establish that reputation (e.g., were unobserved during the manipulation), then we would not expect a moral licensing effect. If you were more observed during the manipulation, the theory is less clear. Here we would expect that participants may return to their baseline moral behaviours (i.e., how they would behave when unobserved), however this may be difficult to do in an experimental context. If participants were observed, then immediately unobserved, this may have created expectancy effects. Future research with more rigorous methods is needed to untangle these effects.

We’d like to note that the results of this analysis should be interpreted with caution and be seen as preliminary (at best). Indeed, this analysis was exploratory and not pre-registered, but more importantly, the groupings of more/less/same observation during the DV are not clear -those with no change in observation include all studies that had no change in observation between the manipulation and DV – whether unobserved or explicitly observed. Similarly, being more/less observed during the DV could be going from unobserved to some observation or to explicit observation, which would be associated with different effect sizes (if not different predictions). Thus, clear experimental manipulation is required to confirm this result.

## B.4 – Bayesian Analyses: Neutral Controls Only

These analyses exclude donut designs, which compare experimental effects with negative rather than neutral controls, retaining only effects compared to neutral controls.

**Table S7**

*Robust Bayesian Meta-Analysis (RoBMA) Model Summaries for Analyses Only Including Neutral Controls*

| Analysis | Test | Models | Prior probability | Posterior probability | Hedge’s *g* (estimate) | Bayes Factor (BF)^a^ |
| --- | --- | --- | --- | --- | --- | --- |
| Overall Model | Effect | 18/36 | 0.50 | 0.51 | -0.05  [-0.16, 0.00] | 1.03 |
|  | Heterogeneity | 18/36 | 0.50 | 1.00 |  | 9.84 x 10^7^ |
|  | Bias | 32/36 | 0.50 | 1.00 |  | 5.76 x 10^6^ |
| No observation condition | Effect | 18/36 | 0.50 | 0.45 | -0.04  [-0.14, 0.00] | 0.81 |
|  | Heterogeneity | 18/36 | 0.50 | 1.00 |  | 5.58 x 10^11^ |
|  | Bias | 32/36 | 0.50 | 1.00 |  | 2.08 x 10^3^ |
| Some observation condition | Effect | 18/36 | 0.50 | 0.32 | -0.03  [-0.17, 0.00] | 0.46 |
|  | Heterogeneity | 18/36 | 0.50 | 0.95 |  | 19.01 |
|  | Bias | 32/36 | 0.50 | 1.00 |  | 6.94 x 10^8^ |
| Explicit observation condition | Effect | 18/36 | 0.50 | 0.94 | 0.58  [0.00, 0.81] | 15.98 |
|  | Heterogeneity | 18/36 | 0.50 | 0.23 |  | 0.29 |
|  | Bias | 32/36 | 0.50 | 0.60 |  | 1.47 |

*^a^Note.* Negative controls (donut designs) were excluded from these analyses. We interpret Bayes Factor (BF) as: substantial evidence for an effect, values ≥ 10; moderate evidence as values between 3 and 10; anecdotal evidence for an effect, values between 1 and 3; anecdotal evidence against an effect, values between 1 and 1/3; moderate evidence against an effect, values between 1/3 and 1/10, substantial evidence against an effect, values ≤ 1/10. All model diagnostic were met (all R-hat values < 1.01; all ESS > 500).

## B.5 – Bayesian Sensitivity Analyses

Sensitivity analyses were conducted to examine how varying prior distributions influenced the results of the RoBMA analyses. Below, we report the outcomes for the overall moral licensing effect and for each observation condition: no observation, some observation, and explicit observation.

For these analyses, we applied six distinct prior distributions, described in detail below. Each prior was consistently applied across all groups to ensure comparability: the overall model, no observation, some observation, and explicit observation.

***Informed Priors Distributions of Effect Sizes***

As recommended by Bartos and colleagues (2024), we computed three sensitivity analyses using informed prior distributions based on the literature for effect sizes:

1. d ~ Cauchy(location = 0, scale = 0.707). This is a default prior distribution in Bayes factor testing, appropriate when large effects cannot be ruled out,
2. d ~ Student−t[0, ∞](location = 0.35, scale = 0.102, df = 3). This is an informed prior distribution for small to medium effects, known as the “Oosterwijk prior distribution”, and
3. δ ~ Normal [0,] (M = 0.30, SD = 0.15). This is an informed prior distribution for small to medium effects, called the “Vohs prior distribution”.

As recommended by Bartos and colleagues (2024), we computed one sensitivity analyses using informed prior distributions based on heterogeneity:

1. Inverse-Gamma (shape = 1, scale = 0.15) empirical prior distribution for the heterogeneity parameter τ of the Cohen’s d effect size.

Lastly, we used two prior distributions for effect size based on the meta-analytic results obtained from prior meta-analyses on moral licensing:

1. Normal distribution, M = 0.31, 95CI [.23, .38], SD = 0.37, (Blanken et al., 2015).
2. Normal distribution, M = 0.11, 95CI [.05, .17], SD = 0.41, (Ferguson et al., 2024).

### B.5.1 – Sensitivity Analyses: Overall Model

Sensitivity analyses for the overall model, which included all moral licensing studies that fit our inclusion criteria, are presented in Table S8. Results were:

- One effect with substantial evidence against an effect (values less than 1/10; BF = 0.02)
- One effect with moderate evidence against an effect (values between 1/3 and 1/10; BF = 0.23)
- Two effects with anecdotal evidence against an effect (values between 1 and 1/3; BFs = 0.04 to 0.88)
- Two effects with anecdotal evidence for an effect (values between 1 and 3; BFs = 1.61 to 2.27)

Notably, in 5/6 of the analyses, the effect returned was negative, indicating a moral consistency effect rather than a moral licensing effect. These results are consistent with the analysis in the main text which found anecdotal evidence for an effect. Combined, these analyses, which correct for publication bias, indicate that (i) when combined in one analysis, we do not find a moral licensing effect, and (ii) this result is analytically robust to different prior distributions.

All models found substantial evidence for heterogeneity, indicating that there is substantial variation among the included studies. Likewise, all models found substantial evidence for publication bias, with unpublished effects being under-represented in the current effort. As a result, without applying a bias correction (e.g., in the multilevel analyses presented in main text), the summary effect size is likely over-estimated, given that unpublished studies usually have smaller effects than published ones.

**Table S8**

Robust Bayesian Meta-Analysis (RoBMA) Sensitivity Analyses of the Overall Moral Licensing Effect, Using Six Different Prior Distributions

| Prior  Distributions | Test | Models | Prior probability | Posterior probability | Hedge’s *g* (estimate) | Bayes Factor (BF)^a^ |
| --- | --- | --- | --- | --- | --- | --- |
| Blanken et al. 2015  (normal distribution,  *d* = 0.31, *SD* = 0.37) | Effect | 18/36 | 0.50 | 0.62 | -0.06  [-0.13, 0.01] | 1.61 |
|  | Heterogeneity | 18/36 | 0.50 | 1.00 |  | 6.55 x 10^10^ |
|  | Bias | 32/36 | 0.50 | 1.00 |  | 4.16 x 10^7^ |
| Ferguson et al. 2024  (normal distribution,  *g* = 0.11, *SD* = 0.41) | Effect | 18/36 | 0.50 | 0.69 | -0.06  [-0.13, 0.01] | 2.27 |
|  | Heterogeneity | 18/36 | 0.50 | 1.00 |  | 7.27 x 10^10^ |
|  | Bias | 32/36 | 0.50 | 1.00 |  | 5.19 x 10^7^ |
| Vohs prior distribution ^b^ | Effect | 18/36 | 0.50 | 0.29 | -0.06  [-0.13, 0.03] | 0.40 |
|  | Heterogeneity | 18/36 | 0.50 | 1.00 |  | 4.32 x 10^10^ |
|  | Bias | 32/36 | 0.50 | 1.00 |  | 1.46 x 10^7^ |
| Priors for when large effects can’t be ruled out ^c^ | Effect | 18/36 | 0.50 | 0.47 | -0.06  [-0.14, 0.01] | 0.88 |
|  | Heterogeneity | 18/36 | 0.50 | 1.00 |  | 7.33 x 10^10^ |
|  | Bias | 32/36 | 0.50 | 1.00 |  | 5.01 x 10^7^ |
| Oosterwijk prior distribution ^d^ | Effect | 18/36 | 0.50 | 0.18 | -0.06  [-0.14, 0.02] | 0.23 |
|  | Heterogeneity | 18/36 | 0.50 | 1.00 |  | 6.14 x 10^10^ |
|  | Bias | 32/36 | 0.50 | 1.00 |  | 2.24 x 10^7^ |
| Prior distribution on heterogeneity ^e^ | Effect | 18/36 | 0.50 | 0.02 | 0.00  [0.00, 0.00] | 0.02 |
|  | Heterogeneity | 18/36 | 0.50 | 1.00 |  | 7.67 x 10^10^ |
|  | Bias | 32/36 | 0.50 | 1.00 |  | 4.92 x 10^7^ |

*Notes.* **^a^**We interpret Bayes Factor (BF) as: substantial evidence for an effect, values ≥ 10; moderate evidence as values between 3 and 10; anecdotal evidence for an effect, values between 1 and 3; anecdotal evidence against an effect, values between 1 and 1/3; moderate evidence against an effect, values between 1/3 and 1/10, substantial evidence against an effect, values ≤ 1/10. All model diagnostic were met (all R-hat values < 1.01; all ESS > 500).

^b^ Informed prior distribution for small- to medium-sized effects (Normal distribution, M = 0.30, SD = 0.15), as recommended for sensitivity analyses by Bartos and colleagues (2022)

^c^ d ~ Cauchy(location = 0, scale = 0.707)—a default prior distribution in Bayes factor testing, appropriate when large effects cannot be ruled out, as recommended for sensitivity analyses by Bartos and colleagues (2022)

^d^ Student−t[0, ∞](location = 0.35, scale = 0.102, df = 3); an informed prior distribution for small- to medium-sized effects, as recommended for sensitivity analyses by Bartos and colleagues (2022)

^e^ Inverse Gamma (shape = 1, scale = 0.15) empirical prior distribution for the heterogeneity parameter τ of the Cohen’s d effect size

### B.5.2 – Sensitivity Analyses: ‘No Observation’ Model

Sensitivity analyses of the ‘no observation’ model are presented in Table S9. Results indicated either anecdotal evidence against an effect (values between 1 and 1/3; four effects) or moderate evidence against an effect (values between 1/3 and 1/10; two effects).

These results are consistent with the analysis in the main text which found anecdotal evidence against an effect. Combined, these analyses, which correct for publication bias, indicate that (i) unobserved studies (i.e., those without opportunity for real social cues) do not elicit a moral licensing effect, and (ii) this result is analytically robust to different prior distributions.

All models found substantial evidence for heterogeneity, indicating that there is substantial variation among the included studies. Likewise, all models found substantial evidence for publication bias, with more published effects included in the analysis compared to unpublished effects. As a result, without applying a bias correction (e.g., in the multilevel analyses presented in main text), the summary effect size is likely over-estimated, given that unpublished studies usually have smaller effects than published ones.

**Table S9**

Robust Bayesian Meta-Analysis (RoBMA) Sensitivity Analyses of the Moral Licensing Effect in the No Observation Condition, Using Six Different Prior Distributions

| Priors | Test | Models | Prior probability | Posterior probability | Hedge’s *g* (estimate) | Bayes Factor (BF)^a^ |
| --- | --- | --- | --- | --- | --- | --- |
| Blanken et al. 2015  (normal distribution,  *d* = 0.31, *SD* = 0.37) | Effect | 18/36 | 0.50 | 0.15 | -0.01  [-0.09, 0.00] | 0.17 |
|  | Heterogeneity | 18/36 | 0.50 | 1.00 |  | 7.59 x 10^8^ |
|  | Bias | 32/36 | 0.50 | 1.00 |  | 1026.78 |
| Ferguson et al. 2024  (normal distribution,  *g* = 0.11, *SD* = 0.41) | Effect | 18/36 | 0.50 | 0.19 | -0.01  [-0.10, 0.00] | 0.23 |
|  | Heterogeneity | 18/36 | 0.50 | 1.00 |  | 7.29 x 10^8^ |
|  | Bias | 32/36 | 0.50 | 1.00 |  | 1033.03 |
| Vohs prior distribution ^b^ | Effect | 18/36 | 0.50 | 0.04 | 0.00  [0.00, 0.05] | 0.04 |
|  | Heterogeneity | 18/36 | 0.50 | 1.00 |  | 5.51 x 10^8^ |
|  | Bias | 32/36 | 0.50 | 1.00 |  | 347.86 |
| Priors for when large effects can’t be ruled out ^c^ | Effect | 18/36 | 0.50 | 0.10 | -0.00  [-0.08, 0.00] | 0.11 |
|  | Heterogeneity | 18/36 | 0.50 | 1.00 |  | 8.67 x 10^8^ |
|  | Bias | 32/36 | 0.50 | 1.00 |  | 1550.10 |
| Oosterwijk prior distribution ^d^ | Effect | 18/36 | 0.50 | 0.18 | -0.01  [-0.10, 0.00] | 0.21 |
|  | Heterogeneity | 18/36 | 0.50 | 1.00 |  | 2.69 x 10^7^ |
|  | Bias | 32/36 | 0.50 | 1.00 |  | 647.22 |
| Prior distribution on heterogeneity ^e^ | Effect | 18/36 | 0.50 | 0.04 | 0.00  [0.00, 0.05] | 0.04 |
|  | Heterogeneity | 18/36 | 0.50 | 1.00 |  | 5.47 x 10^8^ |
|  | Bias | 32/36 | 0.50 | 1.00 |  | 345.01 |

*Notes.* **^a^**We interpret Bayes Factor (BF) as: substantial evidence for an effect, values ≥ 10; moderate evidence as values between 3 and 10; anecdotal evidence for an effect, values between 1 and 3; anecdotal evidence against an effect, values between 1 and 1/3; moderate evidence against an effect, values between 1/3 and 1/10, substantial evidence against an effect, values ≤ 1/10. All model diagnostic were met (all R-hat values < 1.01; all ESS > 500).

^b^ Informed prior distribution for small- to medium-sized effects (Normal distribution, M = 0.30, SD = 0.15), as recommended for sensitivity analyses by Bartos and colleagues (2022)

^c^ d ~ Cauchy(location = 0, scale = 0.707)—a default prior distribution in Bayes factor testing, appropriate when large effects cannot be ruled out, as recommended for sensitivity analyses by Bartos and colleagues (2022)

^d^ Student−t[0, ∞](location = 0.35, scale = 0.102, df = 3); an informed prior distribution for small- to medium-sized effects, as recommended for sensitivity analyses by Bartos and colleagues (2022)

^e^ Inverse Gamma (shape = 1, scale = 0.15) empirical prior distribution for the heterogeneity parameter τ of the Cohen’s d effect size

### B.5.3 – Sensitivity Analyses: ‘Some Observation’ Model

Sensitivity analyses of the ‘some observation’ model are presented in Table S10. Results indicated either anecdotal evidence against an effect (values between 1 and 1/3; three effects) , moderate evidence against an effect (values between 1/3 and 1/10; two effects), and one estimate presenting anecdotal evidence for an effect (BF = 1.32). These results are consistent with the analysis in the main text which found moderate evidence against an effect (BF = 0.19). Combined, these analyses, which correct for publication bias, indicate that (i) ‘some observation’ (i.e., those with opportunity for real social cues, without direct observation) do not elicit a moral licensing effect, and (ii) this result is analytically robust to different prior distributions.

All models found substantial evidence for heterogeneity, indicating that there is substantial variation among the included studies. Likewise, all models found substantial evidence for publication bias, with unpublished effects being under-represented in the sample. Thus, a bias correction is necessary to interpret the effect size estimate; the effect size from the multi-level models is likely over-estimated.

**Table S10**

Robust Bayesian Meta-Analysis (RoBMA) Sensitivity Analyses of the Moral Licensing Effect in the Some Observation Condition, Using Six Different Prior Distributions

| Priors | Test | Models | Prior probability | Posterior probability | Hedge’s *g* (estimate) | Bayes Factor (BF)^a^ |
| --- | --- | --- | --- | --- | --- | --- |
| Blanken et al. 2015  (normal distribution,  *d* = 0.31, *SD* = 0.37) | Effect | 18/36 | 0.50 | 0.35 | 0.03  [-0.06, 0.19] | 0.54 |
|  | Heterogeneity | 18/36 | 0.50 | 1.00 |  | 224.22 |
|  | Bias | 32/36 | 0.50 | 1.00 |  | 463.79 |
| Ferguson et al. 2024  (normal distribution,  *g* = 0.11, *SD* = 0.41) | Effect | 18/36 | 0.50 | 0.37 | 0.03  [-0.09, 0.19] | 0.59 |
|  | Heterogeneity | 18/36 | 0.50 | 1.00 |  | 215.94 |
|  | Bias | 32/36 | 0.50 | 1.00 |  | 551.61 |
| Vohs prior distribution ^b^ | Effect | 18/36 | 0.50 | 0.38 | 0.05  [0.00, 0.22] | 0.63 |
|  | Heterogeneity | 18/36 | 0.50 | 1.00 |  | 220.48 |
|  | Bias | 32/36 | 0.50 | 1.00 |  | 222.16 |
| Priors for when large effects can’t be ruled out ^c^ | Effect | 18/36 | 0.50 | 0.21 | 0.01  [-0.07, 0.16] | 0.27 |
|  | Heterogeneity | 18/36 | 0.50 | 1.00 |  | 361.56 |
|  | Bias | 32/36 | 0.50 | 1.00 |  | 998.01 |
| Oosterwijk prior distribution ^d^ | Effect | 18/36 | 0.50 | 0.22 | 0.03  [0.00, 0.22] | 0.28 |
|  | Heterogeneity | 18/36 | 0.50 | 1.00 |  | 449.82 |
|  | Bias | 32/36 | 0.50 | 1.00 |  | 195.59 |
| Prior distribution on heterogeneity ^e^ | Effect | 18/36 | 0.50 | 0.57 | 0.06  [0.00, 0.19] | 1.32 |
|  | Heterogeneity | 18/36 | 0.50 | 0.99 |  | 94.76 |
|  | Bias | 32/36 | 0.50 | 1.00 |  | 502.55 |

*Notes.* **^a^**We interpret Bayes Factor (BF) as: substantial evidence for an effect, values ≥ 10; moderate evidence as values between 3 and 10; anecdotal evidence for an effect, values between 1 and 3; anecdotal evidence against an effect, values between 1 and 1/3; moderate evidence against an effect, values between 1/3 and 1/10, substantial evidence against an effect, values ≤ 1/10. All model diagnostic were met (all R-hat values < 1.01; all ESS > 500).

^b^ Informed prior distribution for small- to medium-sized effects (Normal distribution, M = 0.30, SD = 0.15), as recommended for sensitivity analyses by Bartos and colleagues (2022)

^c^ d ~ Cauchy(location = 0, scale = 0.707)—a default prior distribution in Bayes factor testing, appropriate when large effects cannot be ruled out, as recommended for sensitivity analyses by Bartos and colleagues (2022)

^d^ Student−t[0, ∞](location = 0.35, scale = 0.102, df = 3); an informed prior distribution for small- to medium-sized effects, as recommended for sensitivity analyses by Bartos and colleagues (2022)

^e^ Inverse Gamma (shape = 1, scale = 0.15) empirical prior distribution for the heterogeneity parameter τ of the Cohen’s d effect size

### B.5.4 – Sensitivity Analyses: ‘Explicit Observation’ Model

Sensitivity analyses of the ‘explicit observation’ model are presented in Table S11. Results were: three effects demonstrated substantial evidence for an effect (values > 10; BFs: 11.39 to 18.88), two effects demonstrated moderate evidence for an effect (values between 3 and 10; 5.81 to 9.80), and only one resulted in anecdotal evidence against an effect (BF = 0.73). These results are consistent with the analysis in the main text, which found moderate evidence for an effect (BF = 9.14). Combined, these analyses, which correct for publication bias, indicate that (i)observed studies (i.e., those with the opportunity for real social cues) elicit a moral licensing effect, and (ii) this result is analytically robust to different prior distributions.

Models in this analysis mostly found anecdotal evidence against heterogeneity, indicating that these are comparable studies. All models found moderate (or substantial) evidence for publication bias, with published effects being over-represented. As a result, without applying a bias correction (e.g., in the multilevel analyses presented in main text), the summary effect size is likely over-estimated, given that unpublished studies usually have smaller effects than published ones.

**Table S11**

Robust Bayesian Meta-Analysis (RoBMA) Sensitivity Analyses of the Moral Licensing Effect in the Explicit Observation Condition, Using Six Different Prior Distributions

| Priors | Test | Models | Prior probability | Posterior probability | Hedge’s *g* (estimate) | Bayes Factor (BF)^a^ |
| --- | --- | --- | --- | --- | --- | --- |
| Blanken et al. 2015  (normal distribution,  *d* = 0.31, *SD* = 0.37) | Effect | 18/36 | 0.50 | 0.94 | 0.47  [0.00, 0.75] | 16.87 |
|  | Heterogeneity | 18/36 | 0.50 | 0.34 |  | 0.52 |
|  | Bias | 32/36 | 0.50 | 0.86 |  | 6.04 |
| Ferguson et al. 2024  (normal distribution,  *g* = 0.11, *SD* = 0.41) | Effect | 18/36 | 0.50 | 0.92 | 0.44  [0.00, 0.74] | 11.39 |
|  | Heterogeneity | 18/36 | 0.50 | 0.37 |  | 0.58 |
|  | Bias | 32/36 | 0.50 | 0.88 |  | 7.24 |
| Vohs prior distribution ^b^ | Effect | 18/36 | 0.50 | 0.95 | 0.40  [0.00, 0.63] | 18.88 |
|  | Heterogeneity | 18/36 | 0.50 | 0.38 |  | 0.61 |
|  | Bias | 32/36 | 0.50 | 0.95 |  | 17.51 |
| Priors for when large effects can’t be ruled out ^c^ | Effect | 18/36 | 0.50 | 0.85 | 0.42  [0.00, 0.75] | 5.81 |
|  | Heterogeneity | 18/36 | 0.50 | 0.39 |  | 0.64 |
|  | Bias | 32/36 | 0.50 | 0.87 |  | 6.59 |
| Oosterwijk prior distribution ^d^ | Effect | 18/36 | 0.50 | 0.42 | 0.22  [0.00, 0.74] | 0.73 |
|  | Heterogeneity | 18/36 | 0.50 | 0.56 |  | 1.29 |
|  | Bias | 32/36 | 0.50 | 0.92 |  | 11.95 |
| Prior distribution on heterogeneity ^e^ | Effect | 18/36 | 0.50 | 0.91 | 0.40  [0.00, 0.73] | 9.80 |
|  | Heterogeneity | 18/36 | 0.50 | 0.41 |  | 0.69 |
|  | Bias | 32/36 | 0.50 | 0.90 |  | 0.40 |

*Notes.* **^a^**We interpret Bayes Factor (BF) as: substantial evidence for an effect, values ≥ 10; moderate evidence as values between 3 and 10; anecdotal evidence for an effect, values between 1 and 3; anecdotal evidence against an effect, values between 1 and 1/3; moderate evidence against an effect, values between 1/3 and 1/10, substantial evidence against an effect, values ≤ 1/10. All model diagnostic were met (all R-hat values < 1.01; all ESS > 500).

^b^ Informed prior distribution for small- to medium-sized effects (Normal distribution, M = 0.30, SD = 0.15), as recommended for sensitivity analyses by Bartos and colleagues (2022)

^c^ d ~ Cauchy(location = 0, scale = 0.707)—a default prior distribution in Bayes factor testing, appropriate when large effects cannot be ruled out, as recommended for sensitivity analyses by Bartos and colleagues (2022)

^d^ Student−t[0, ∞](location = 0.35, scale = 0.102, df = 3); an informed prior distribution for small- to medium-sized effects, as recommended for sensitivity analyses by Bartos and colleagues (2022)

^e^ Inverse Gamma (shape = 1, scale = 0.15) empirical prior distribution for the heterogeneity parameter τ of the Cohen’s d effect size

## B.6 – Meta-Regressions

Meta-regressions can be used to determine which moderators have the most impact on the moral licensing effect. Categorical moderators are dummy coded; estimated differences in effect sizes are compared among two moderator subgroups (e.g., differences for both the *explicit* and *some observation* subgroups are compared to the *no observation* subgroup)

**Robustness Check 1: Observation Condition vs Study Location**

To examine the influence of observation condition compared to location of study on the moral licensing effect, we conducted a meta-regression. We included observation condition (no observation, some observation, explicit observation) and study location (online vs in-person). We used a multilevel random-effects model, accounting for multiple effect sizes within studies.

Before running the model, we tested for multicollinearity among predictors. Variance Inflation Factors (VIFs) were within acceptable limits (VIF < 1.59), indicating that the predictors were sufficiently independent to be included in the same model.

***Results******.*** The model was significant, *F*(3,108) = 3.41, *p* = .020; residual heterogeneity remained significant, *QE*(108) = 268.19, *p* < .001.

Observation condition significantly moderated the moral licensing effect; larger licensing effects observed in the *explicit observation* condition compared to no *observation condition*, *t*(107) = 2.66, *p* = .008, however there was no difference between the *no observation* and *some observation* conditions, *t*(107) = 1.44, *p* = .152. In this model, study location (online vs. in-person) did not significantly predict effect size variation, *t*(107) = -0.35, *p* = .721.

Thus, explicit observation best predicts moral licensing effect size in this model.

**Robustness Check 2: Observation Condition, Study Location, Publication Status, Manipulation Type, Control Condition**

In this analysis, we included observation condition (no observation, some observation, explicit observation), study location (online vs, in-person), publication status (published vs. unpublished), manipulation type^[[2]](#footnote-3)^ (imagined, writing primes, recall tasks, and behavioural tasks), and control condition (negative vs. neutral)^[[3]](#footnote-4)^. Note that ‘intended behaviour’ manipulations were excluded because there were too few studies (*k* =5).

***Results.*** The model was significant, *F*(8,98) = 2.97, *p* = .005. Residual heterogeneity remained significant in this model, *QE*(98) = 226.00, *p* < .001.

Again, observation condition significantly moderated the moral licensing effect; larger licensing effects observed in the *explicit observation* condition compared to no *observation condition*, *t*(98) = 2.23, *p* = .028, with no difference between the *no observation* and *some observation* conditions, *t*(98) = 1.57, *p* = .118. Published studies had larger effects than unpublished studies, *t*(98) = 2.75, *p* = .007. All other methods factors were non-significant: study location (online vs. in-person), *t*(98) = 0.07, *p* = .994; manipulation types, *t*s(98) < 1.88, *p*s > .063; and control condition, *t*(98) = 0.57, *p* = .568.

Thus, explicit observation and publication bias are the bests predictors in this model.

## B.7 – Pre-Registered Analyses

Below we present the analyses specified in our pre-registration. We include them to demonstrate that the results from the pre-registered analyses are analytically robust to analytic method (i.e., results are consistently in the same direction, despite analytic strategy). The analyses in the main text are more suitable for the data structure of the present analysis.

In our pre-registration, we specified that standard two-level meta-analyses would be conducted in R using the metafor package and that publication bias would be assessed using a funnel plot. That these analyses do not correct for the multilevel nature of the data and use PET-PEESE as a bias correction. Although PET-PEESE has been widely used in psychological meta-analyses since it was introduced, it has been found to be overly conservative. As such, we recommend only interpret these results as convergent evidence for the analyses in text; they are less appropriate given recent advances in meta-analysis. Note that we did not pre-register the bias-correction technique at the time of the pre-registration; we provide justification below for this choice.

***Publication Bias.*** The funnel plot was found to be asymmetrical (*z* = 4.03, *p* < .001), which is suggestive of publication bias (see *Figure S1*).


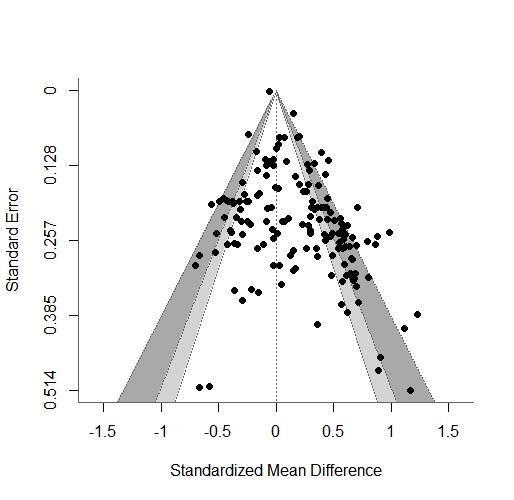


**Figure S1.** Funnel plot of the effect sizes (Cohen’s d) and standard errors of each study (dots) included in the overall analysis. The dark grey areas represent experiments with effect sizes that were statistically significant at p < .05, while the light grey area represents marginal significance (i.e., .10 > p > .05). Studies that fall outside of this area are highly statistically significant (p < .01). The vertical reference line has been set to zero, and the center line does not reflect the mean effect size. The funnel plot was found to be asymmetrical (z = 3.72, p < .001), which is suggestive of publication bias.

***Analytic Approach.*** To begin, we computed naïve (uncorrected) models for the overall effect and moderator analyses. Subsequently, we applied the precision-effect test with standard error (PET-PEESE) to correct for publication bias for the overall model and moderator variables (Stanley & Doucouliagos, 2014; Stanley 2017). We first applied PET, then if the variable intercept was not significant at *p* < .100, we applied PEESE, as per the recommendations by Stanley (2017). To test the effects of the methodological moderators in the presence of others, we also computed random-effects meta-regression models (both naïve and corrected with PET-PEESE), which were weighted by inverse variance. Because moderators were categorical, we dichotomized them into low and high categories, and dummy coded as 0 or 1. These are further described in the results section. When there was missing data, those effect sizes were omitted from the respective analyses.

PET-PEESE is a combination of two approaches: the precision-effect test (PET) and the precision-effect estimate (PEESE). PET fits a linear regression to the tested relationship to estimate the true effect after correcting for small study effects and publication bias, while PEESE fits a quadratic relationship between the observed effect size and standard error to correct for bias. PET and PEESE perform better under different conditions, which PET-PEESE addresses by using the statistical significance of the PET estimate to determine whether the PET or PEESE estimate is used as the final result (Carter, Schönbrodt, Gervais, & Hilgard, 2017; Stanley & Doucouliagos, 2014).

This method has been used in a number of recent meta-analyses (e.g. Carter et al., 2015; Kuper & Bott, 2018; Lethonen et al., 2018). Moreover, computational studies suggest that PET-PEESE out-performs other commonly-used bias-correction methods, such as trim-and-fill, the weighted average of adequately powered studies, and p-curve methods (Becker, 2005; Stanley, 2017; Carter, Schönbrodt, Gervais, & Hilgard, 2017). However, we must note that PET-PEESE has several limitations. These include poor performance when there is a small number of studies, small sample sizes across all studies, and under high heterogeneity which results in both over and under estimations of effect sizes (Stanley, 2017). Based on Stanley’s (2017) recommendation that we do not use this method when heterogeneity exceeds (*I^2^*) 80%, this model is appropriate for use in the present study. We must caution that the PET-PEESE corrections are like an educated guess, and single estimates should be understood as such (Lehtonen et al., 2018). To account for limitations, we encourage others to use our openly-available data to apply other bias-correction methods to determine how it affects the data. Although we did not pre-register analyses correcting for publication bias, we found evidence for publication bias in our sample using a regression test for funnel plot asymmetry, *t*(153) = 3.72, *p* < .001, which justifies these analyses (see *Figure S1*).

Results from these analyses are presented in *Table S12.*

**Table S12**

Naïve (Uncorrected) Random-Effects Moderator Two-Level Meta-Analyses, Corrected for Publication Bias using 3-PSM and PET-PEESE (as Pre-Registered, Not Multilevel Models)

|  | | Uncorrected (Naïve) Models;  Moderation test (grey) | | 3-PSM Bias Corrections | | PET-PEESE Bias Corrections;  Moderation test (grey) | |
| --- | --- | --- | --- | --- | --- | --- | --- |
| Moderator | | *d*  [95CI] | Test statistic | *d*  [95CI] | Test statistic | *d*  [95CI] | Test statistic |
| Overall Model | | 0.16***  [0.10, 0.22] | t(154) = 5.36,  *p* < .001 | -0.02  [-0.08, 0.04] | *z* = -0.59,  *p* = .556 | -0.06***  [-0.07, -0.05] | ***t***(153) *=-*15.04,  *p* < .001 |
| Observation*** | |  | *F*(2, 116) = 10.66,  *p* < .001 |  |  |  | *F*(3, 115) = 9.10,  *p* < .001 |
|  | No Observation | 0.11**  [0.13, 0.19] | *t*(116) = 2.70,  *p* = .008 | -0.01  [-0.09, 0.08] | *z* = -0.13,  *p* = .894 | 0.03  [-0.07. 0.13] | *t*(115) = 0.66,  *p* = .513 |
|  | Some Observation | 0.25***  [0.14, 0.37] | *t*(116) = 4.35.  *p* < .001 | -0.04  [-0.17, 0.08] | *z* = -0.67,  *p* = .502 | 0.14*  [-0.00, 0.29] | *t*(115) = 1.98,  *p* = .050 |
|  | Explicit Observation | 0.68***  [.43, .92] | *t*(116) = 5.52,  *p* < .001 | 0.50***  [0.37, 0.64] | *z* = 7.26,  *p* < .001 | 0.50***  [0.21, 0.78] | *t*(115) = 4.47,  *p* < .001 |
| Location of Study*** | |  | *F*(1, 117) = 17.83,  *p* < .001 |  |  |  | *F*(2, 116) = 13.72,  *p* < .001 |
|  | Online | 0.04  [-0.05, 0.14] | *t*(117) = 0.94,  *p* = .351 | -0.05  [-0.13, 0.02] | *z* = -1.42, *p* = .156 | -0.04  [-0.15, 0.06] | *t*(116) = -0.78,  *p* = .438 |
|  | In-Person | 0.31***  [0.23, 0.40] | *t*(117) = 7.23,  *p* < .001 | 0.06  [-0.05, 0.16] | *z* = 1.09, *p* = .275 | 0.18**  [0.06, 0.30] | *t*(116) = 2.97,  *p* = .004 |
| Ambiguity* | |  | *F*(3, 140) = 3.23,  *p* = .024 |  |  |  | *F*(4, 139) = 4.89,  *p* = .001 |
|  | Little-to-No Ambiguity | 0.22**  [0.07, 0.37] | *t*(140) = 2.96,  *p* = .004 | 0.02  [-0.17, 0.22] | *z* = 0.22, *p* = .825 | 0.11  [-0.05, 0.27] | *t*(139) = 1.40,  *p* = .165 |
|  | Some Ambiguity | 0.10**  [0.01, 0.18] | *t*(140) = 2.29,  *p* = .023 | -0.04  [-0.13, 0.06] | *z* = -0.81, *p* = .417 | -0.01  [-0.11, 0.10] | *t*(139) = -0.14,  *p* = .885 |
|  | Moderate Ambiguity | 0.13  [-0.02, 0.29] | *t*(140) = 1.67,  *p* = .097 | 0.06  [-0.06, 0.17] | *z* = 0.99, *p* = .321 | 0.02  [-0.15, 0.19] | *t*(139) = 0.25,  *p* = .804 |
|  | High Ambiguity | .33***  [0.20, 0.49] | *t*(140) = 5.15,  *p* < .001 | Model did not converge |  | 0.18*  [0.03, 0.34] | *t*(139) = 2.42,  *p* = .017 |

*Notes.* * indicates significance at *p* < .05, ** at *p* < .01, *** at *p* < .001

# C – Discussion

## C.1 – Distinguishing ‘Moral’ Licensing from ‘Other’ Licensing

In this meta-analysis, we chose to only include studies that were broadly moral (defined as being consistent with either moral foundations theory, or morality-as-cooperation hypothesis). This decision was made for the following reasons:

1. To have consistency among studies and study methodology; a common criticism of meta-analyses is that they can ‘compare apples and oranges’ meaning the aggregate effect size estimates are not meaningful because the studies are too different to be averaged and generate an interpretable effect size. Indeed, extremely high heterogeneity has been reported in all the moral licensing meta-analyses.
2. To be comparable with prior moral licensing meta-analytic efforts (Blanken et al., 2015; Simbrunner & Schlegelmilch, 2017)
3. We cannot assume the same mechanism across other domains of licensing (e.g., eating behaviors, exercise, etc.)

Licensing has been reported in many domains, which includes eating behaviours (De Witt Huberts et al., 2012), exercise behaviors (West et al., 2017), and conspicuous consumption (Khan & Dhar, 2006, 2007). Despite the apparent similarity with moral licensing, other domains may have key differences in *why* the licensing behavior happens. For example, engaging in exercise to permit oneself to eat more calories is common practice and advice in fitness circles. Indeed, people who exercise more require more calories throughout the day. This mental calculation may be learned and operate different than moral licensing, where doing something ‘good’ to then be ‘bad’ is not culturally-relevant advice. Although we do advance that there can be a reputation-based component here, where if one wants to signal a desired trait (eating healthy, exercise regularly) they can do so, then subsequently engage in those behaviors to a lesser degree and still maintain that reputation when someone is watching, it may not be the only mechanism operating here. As such, we chose to exclude licensing behaviors in other domains from the present study. We encourage other researchers in these domains to investigate *when and why* licensing occurs for those behaviors, to compare similarities and/or differences to the moral licensing effect.

We also thank a reviewer for also highlighting the similarity to Hollander’s concept of “idiosyncrasy credits” (Hollander, 1958) – people who have proven their normativity to others are subsequently more able to break norms by being idiosyncratic. In these studies, initially making the ‘good’ choice (e.g., eating healthy, exercising) subsequently licenses the participants to make a less ‘good’ choice.

## C.2 – How Moral Reputation can be Judged Across Domains

Licensing has been found to not only affect DVs in the same domain as the manipulation, but also across domains (Mazar & Zhong, 2010; Miller & Effron, 2010). For example, a pro-environmental license has been found to affect cheating behaviour (Mazar & Zhong, 2010). Two previous meta-analyses have found that domain consistency did not moderate the licensing effect (Blanken et al., 2015; Simbrunner & Schlegelmilch, 2017).

We propose that this may be because of *what* is being signaled. In the domains we examine (cooperation, discrimination, and environmental), the specific information being communicated may be different (e.g., honesty, valuing others, non-discrimination, pro-environmentalism), however all domains share a single unifying characteristic: that the actor has moral/prosocial intentions. For example, people’s environmentalism is correlated with their cooperation because both are caused by their prosocial intentions (Barclay & Barker, 2020).

Thus, because these variables share an underlying cause, a moral licensing effect would be the same magnitude regardless of whether the licensing manipulation and DV are in the same or different; similar information is being communicated in both domains. If the information was different across the two tasks (e.g., one non-moral task which does not signal morality, such as physical strength and the other prosocial behavior) we would not expect a licensing effect, unless there is a ‘halo effect’ across all positive judgments.

## C.3 – Is Moral Licensing an Effect of Observation or Study Location?

Which is the stronger effect, observation or location of study? To compare among these two (related) conditions and determine whether this effect is driven by observation or the location of the study from theoretic and empirical standpoint, we compare the moderation analysis for the no observation condition directly to the explicit observation condition. When the ‘some observation’ condition is excluded, the moderation result is stronger than the in lab vs online moderation result (Observation: *F*(1, 79) = 11.27, *p =* .001; Location: *F*(1, 117) = 7.8, *p =* .006). As such, it’s more likely that the effect of “online vs. in-person” is driven by the higher levels of observation in-person, rather than effect of “observed vs. non-observed” being an epiphenomenon of something else that happens to differ online vs. in-person.

We argue that both the observation and location analyses address the same underlying mechanism, i.e., whether there are more (vs fewer) interpersonal cues. We believe the stronger analysis is the observation condition because it is more constant; by judging the study descriptions to determine if there were (1) direct observation and social interactions (i.e., explicit observation), (2) opportunities for social interactions and observation (i.e., some observation), and (3) no opportunity for social interactions or observation. This will be more variable in the location analysis, as in-lab studies could vary from no observation to high observation (or no social cues to high social cues).

## C.4 – Results Discussion for Additional Methodological Moderators

**Publication Bias.** Unsurprisingly, there were larger moral licensing effects among published studies compared to unpublished studies, which was consistent with prior meta-analyses (Blanken et al., 2015; Simbrunner & Schlegelmilch, 2017).

**Control Condition.** Interestingly, we did not find a moderating effect of control condition; consistent with the results from Blanken and colleagues (2015). Notably, the effect estimates were in the predicted direction, with studies with neutral controls (Hedge’s *g =* .17) having a smaller effect than those with negative controls (Hedge’s *g* = 0.26; donut designs).

**Domain.** To our knowledge, our study was the first analysis to estimate the moral licensing effect by domain, which returned similar estimates across domains. This suggests that moral licensing is likely a similar effect across types of moral behaviours. Similarly, domain consistency (i.e., same vs different) did not moderate the moral licensing effect. This analysis replicated the findings of two previous meta-analyses (Blanken et al., 2015; Simbrunner & Schlegelmilch, 2017). Interestingly, this is contrary to mental accounting theory (Thaler, 1985), which proposes that people are more likely to behave immorally within the same domain. The theoretical rationale for domain consistency is discussed further below, in section C.5.

**Monetary DV.** We predicted that DVs in which participants could earn money would produce smaller licensing effects than hypothetical measures because paid DVs were likely less ambiguous than hypothetical DVs. Moreover, studies in other domains have found payment to reduce effect sizes (Amir et al., 2012; Rotella et al., 2019). Although there was a larger effect size estimate for hypothetical dependent measures (Hedge’s *g =* .24) compared to monetary dependent measures (Hedge’s *g =* .17), the moderator analysis was not significant.

**Manipulation Type.** The type of manipulation did not significantly moderate the moral licensing effect. However, this overall finding should be interpreted with caution, as the five manipulation categories varied in sample size—some quite small (see further discussion in C.6).

Reporting future intended behavior produced the largest moral licensing effect. However, this finding is based on only five studies, several of which involved participants being explicitly observed. As a result, the effect is confounded by observation. Therefore, we cannot draw strong conclusions about the specific role of intended behavior alone.

Recall tasks, behavioral tasks, and imagining tasks all showed small moral licensing effects. Only writing primes failed to produce a significant effect. Again, these outcomes may be confounded with other methodological variables.

**Participant Culture.** Lastly, we aimed to replicate Simbrunner and Schlegelmilch’s (2017) moderating effect of participant culture on moral licensing. Although we did find a significant moderation, we argue that these estimates should not be interpreted. Notably, our analysis expanded the cultural groups and included Australia, Africa, and the UK, in addition to the original three (North America, Europe and Sout-East Asia).

With this extension, we note several limitations. For the majority of these geographic regions, there are few estimates. Secondly, the cultural pattern is unclear. Notably, performing analyses like this in meta-analysis are of limited value because of confounding factors –the efforts for each geographic region are usually done by the same lab group, which may differ on many factors, such as methods. As such, we cannot distinguish the effect of culture or methods. Given this, we caution against the use of cultural comparisons in meta-analysis unless we eliminate other possible confounds.

## C.5 –Discussion: Domain (In)Consistency and Reputation Judgements in Moral Licensing

Domain consistency did not moderate moral licensing effect in our analyses. At first glance, this might seem inconsistent with a reputation-based account of moral licensing. However, while there is evidence of domain-specific moral judgments, there also is evidence that people make generalized moral character judgments, integrating behaviours across domains into global reputational impressions. These processes aren’t mutually exclusive. If observers generalize reputational judgments, it helps explain why domain consistency did not moderate the moral licensing effect. Here we present evidence for domain general moral judgments:

1. ***Social Networks & Generalized Morality.*** Moral character judgments can be generalized, particularly in large social networks. Studies have shown that as social networks grow larger and more complex (e.g., in larger populations), people tend to perceive morality as a generalized dimension rather than context-specific traits. The authors argue that this allows individuals to predict cooperation and make decisions in novel or unfamiliar situations more efficiently (Jackson et al., 2023).
2. ***Holistic Impressions.*** Research finds that individuals form holistic impressions through the integration of multiple cues. For instance, warmth and competence are often integrated to form global impressions of character (Fiske et al., 2007).
3. ***Judgement Weighting.*** Some studies find that some judgments are weighted more heavily in character judgments (e.g., honesty, fairness, kindness) compared to others (e.g., competence), however, these actions are integrated into assessments rather than relying on a single behaviour (Goodwin et al., 2014). In fact, Effron and Monin (2010) argue, in a foundational paper on moral licensing, that people integrate past moral actions to from a holistic impression.
4. ***Similar Reputation Signals.*** Experimental psychology research finds that cooperative, moral, and environmental behaviours are often judged through the same reputational lens—as indicators of trustworthiness and prosociality (Barclay & Barker, 2020; Barclay & Willer, 2007; Bradley et al., 2018). For example, Bradley, Lawrence, & Ferguson (2018) find that cooperation and morality are similarly influenced by reputational concerns.

***Summary.*** Together, this evidence suggests that people often generalize across domains when forming moral impressions. As a result, the absence of a domain consistency effect in our analysis does not undermine the reputational account of moral licensing. Instead, it aligns with the idea that behaviours from different domains are often judged similarly and integrated into a cohesive moral reputation. Thus, the absence of a domain consistency effect does not undermine the reputational explanation of moral licensing.

***Additional Speculation.*** That said, domain integration may not always occur uniformly. We speculate that the extent to which behaviours across domains are seen as morally related will vary by cultural context, salience of the domain, and audience expectations. For example, in cultures where environmentalism is tightly linked to prosocial identity, pro-environmental behaviours may be more readily integrated with other moral behaviours. In contrast, where environmentalism is not strongly moralized, such behaviours may be less likely to influence moral reputational judgments. Testing this is beyond the scope (and ability) of the current effort but would be interesting avenues for future work. In fact, we expect that the generalizability of moral judgements would influence moral licensing; actions that are informative of a general moral character would produce a licensing effect, whereas disparate (or are domain-specific) actions would be unlikely to produce licensing effects.

## C.6 – How do Different Manipulations Fit Within a Reputational Account of Moral Licensing?

Moral licensing manipulations vary considerably. Below we outline how the different manipulation types relate to a reputation-based account of moral licensing. In our analyses, we included five categories of moral licensing manipulation: intended behaviours, imagined behaviours, writing primes, recall tasks, and real behavioural tasks. Within each category, the tasks and procedures varied considerably; as such

**Intended Behaviors**. While intentions may seem like weak reputational signals (“talk is cheap”), they can still carry reputational weight when publicly expressed. Stating a moral intention (e.g., planning to donate) can function as a reputational commitment, especially in contexts where observation or future accountability is implied. Research shows that people often use self-reported intentions as a form of social signaling, particularly when they know they are being observed (Effron & Monin, 2010). This implies that observers are willing to make character judgments based on stated intentions alone.

While such signals may seem insubstantial, in the absence of additional information, people often rely on minimal cues to make rapid, automatic inferences about others' moral character (Uhlmann et al., 2015). In fact, social psychology research has identified several mechanisms that underlie these quick character judgments, including thin slicing, spontaneous trait inference, and the fundamental attribution error. Taken together, this suggests that publicly expressed intentions can be sufficient for observers to make reputation-based evaluations.

In our dataset, intended behavior manipulations were often used in the *explicit observation* condition. Thus, the larger effect sizes associated with these manipulations are confounded by the presence of reputational cues. That is, the observed effects reflect the combined influence of stated intentions and being observed, rather than intention alone.

Examples of intended behaviour tasks include: Asking participants if they were interested in taking part of a fundraiser, intentions to donate, intentions to engage in pro-environmental behaviours (Cascio & Plant, 2015).

**Imagined Behaviours**. If participants are prompted to imagine performing an action, then these tasks are conceptually less tied to reputation, as they typically lack a public or observable component. If licensing is elicited in these conditions, it would likely be due to intrapsychic motives. However, if participants are asked to describe (verbally or written) and are made aware that others will hear/read the tasks or incorporate other observation-like elements (e.g., experimenter feedback), then they can involve a reputational component. As stated above, people can rely on minimal cues to make rapid, automatic inferences about others' moral character (Uhlmann et al., 2015). It is likely this can extend to descriptions of imagined behaviours.

Examples: Participants imagined themselves volunteering for university or imagine themselves performing a good deed (Clot et al., 2014, 2018).

**Writing Primes**. These manipulations involved writing about positive/moral experiences without recalling a past action. Again, these tasks can convey reputational information if observability is manipulated. In fact, studies in the explicit observation condition used writing primes, where they wrote about themselves using positive words (Sachdeva et al., 2009). However, some tasks may lend themselves more to impression formation of the moral acts, where tasks are more involved by participants (e.g.), compared to ones that do not convey much information about the writer (e.g., coping lists of moral words).

Additional examples include: Writing about positive experiences with a minority (Bradley-Geist et al., 2010b) and describing five good deeds (Young et al., 2012).

**Recall Tasks.** These manipulations involve participants recalling a past moral action. Given that recalling a past action can convey character information, if this information is shared among others in reputation-relevant conditions (e.g., observation), these tasks should elicit moral licensing. Examples include: Writing about a time they were moral or ethical (Cornelissen et al., 2013); recalled a situation where they helped someone else (J. Jordan et al., 2011; Li et al., 2017).

**Behavioural Tasks**. Behavioural manipulations—in which participants perform actual moral actions—are likely to signal moral character, especially when those actions are observable. While the specific behaviours used in these tasks can vary widely, we expect that tasks which clearly communicate moral character will be more effective at producing moral licensing effects.

Examples include: Choosing to hire a minority candidate over a majority candidate (Monin & Miller, 2001), choosing green products (Schwabe et al., 2018; Truelove et al., 2016; Urban et al., 2019), choosing morally good items (Jones & Koenig, 2018).

**Summary.** Not all moral licensing manipulations are equally effective—some signal moral character more clearly than others. From an interpersonal perspective, we predict that licensing manipulations will be most effective when they both (1) clearly convey moral character and (2) engage reputational concerns. In other words, tasks that make moral behaviour easy to interpret, combined with procedures that highlight social evaluation (e.g., public or observable actions, or those framed in reputational terms), are most likely to trigger moral licensing.

Importantly, we caution against drawing conclusions about the effectiveness of manipulation types based on the results from this study (Table 2, main text) because these are confounded with other factors like observation.

## C.7 – Moral Licensing vs Moral Consistency

How would reputation influence when and why we observe a moral consistency effect, a moral licensing effect, or no effect? Here, we elaborate on when and why reputation is theorized to lead to a moral licensing effect or a moral consistency effect (vs no effect). We note that these are theory-based speculations and have not yet been tested empirically.

Moral licensing occurs when someone who initially behaved morally later behaves *less* morally, while moral consistency occurs when someone who initially behaved morally later behaves *more* morally (see Figure 1, main text). How can reputation account for both effects? When might we expect a moral licensing effect compared to no effect? Below we elaborate on three conditions which may calibrate when we observe a moral licensing effect, no effect, or a moral consistency effect. Note that these are not comprehensive conditions, rather they are examples of how reputation-based theory can calibrate moral regulation.

**Observation.** In this paper, we argue that moral licensing occurs when someone establishes a reputation to an observer, which gives them “license” to act subsequently act slightly less morally and maintain that reputation. When no one is observing, we would not predict an effect because one has not established a moral reputation. In this case, there would not be any incentive to appear *more* moral to establish a reputation. The target would have full knowledge of their past moral behaviors and knowledge of their moral identity; it is unlikely that they will calibrate their subsequent moral actions *only* according to the preceding action. Thus, we’d predict that when no one is observing, people will act according to their moral average or baseline. If there is a moral licensing effect based on intrapsychic mechanisms, we would expect that the first moral action would have to be a behavioral outlier, such that is much better than the individual’s ‘average’ moral behaviors, to subsequently justify a license.

With that said, feelings of observation can be elicited through other cues, such as imagining others or through invalid cues such as the watching eyes effect (Bradley et al., 2018). As such, it is possible that moral licensing can still be elicited in the absence of observers. If this does occur, however, we’d predict that it would likely be a smaller effect than one elicited with real social cues.

**Observers.** In the observed moral licensing studies included in this meta-analysis, the same observers witnessed both moral actions. This is important because the moral licensing manipulation would have served to establish a reputation. If this condition was not met, that is that there would be different observers for the first and second moral actions (and participants are aware that there’d be no communication among observers), we would predict no effect because participants would have to establish a ‘first impression’ of moral reputation twice. We’d predict a moral licensing effect only when a moral reputation has already been established. If there were two different observers for the first and second moral tasks, and there would be opportunity for gossip (i.e., sharing information among the two observers), we may expect a moral licensing effect although we speculate that the effect would smaller than for a single observer (i.e., one observer > two observers with communication > two observers without communication). Notably, if the moral licensing manipulation is observed, while the DV is unobserved, it is likely an observation effect, where people are more moral because they are being watched in the first instance (i.e., during the manipulation), but then return to their baseline (unobserved) moral bahaviours in the second instance (i.e., during dependent measure).

## C.8 – Licensing Mechanisms: Moral Credits vs Credentials

Two theories have been proposed to explain how moral licensing happens: moral credits and moral credentials. Moral credits are viewed as a type of currency, where individuals can accrue ‘credits’ by doing good deeds to later pay for bad deeds (Barclay, 2016; Effron & Monin, 2010; Merritt et al., 2010; Miller & Effron, 2010). This is related to reputation-based image-scoring theories, which suggest that reputations are based on scores where prosocial acts increase one’s score and antisocial (or immoral) acts decrease it (Nowak & Sigmund, 1998a, 1998b). However, moral credits theory takes this a step further and suggests that people choose to ‘budget’ their scores based on anticipated future interactions (Merritt et al., 2010).

In contrast to moral credits, it has been proposed that licensing occurs when people have the opportunity to establish credentials (Effron & Monin, 2010; Merritt et al., 2010; Miller & Effron, 2010; Monin & Miller, 2001). That is, when people have demonstrated that they possess a certain characteristic (i.e., they’ve established credentials/reputation), their later behavior that is inconsistent with their original action can be attributed to something other than that characteristic (e.g., a situational factors), and thus they can behave less morally without discrediting themselves. For example, if someone had previously demonstrated that they are not sexist, a later decision to hire a man over a woman can be attributed to something other than a sexist decision. Thus, moral credentials work through a separate mechanism than credits, where one’s perception of the “bad” (or inconsistent) behavior is altered based on prior knowledge.

These two theories vary based on how the second action is interpreted. That is, according to moral credits theory, the second action can still be interpreted in terms of one’s moral character, but this is offset because their *average* moral behavior is better than the score of that single action. On the other hand, according to moral credentials theory, a second behavior that is inconsistent with the first action gets attributed to something other than one’s character (e.g., situational factors). Both theories assume that someone is keeping track of past and present behaviors. However, in both the moral credits and credentials theories it is unclear who the observer is. That is, can these credentials be established to oneself (i.e., self-image) or is it in reference to others (i.e., reputation)? That is the question that we week to address in this research. Further research is necessary to understand the role of credits and credentials in licensing.

# D – PRISMA Checklist

**Table S11**

PRISMA Checklist

| **Section and Topic** | **Item #** | **Checklist item** | **Location where item is reported** |
| --- | --- | --- | --- |
| **TITLE** | | |  |
| Title | 1 | Identify the report as a systematic review. | Yes |
| **ABSTRACT** | | |  |
| Abstract | 2 | See the PRISMA 2020 for Abstracts checklist. | See table below |
| **INTRODUCTION** | | |  |
| Rationale | 3 | Describe the rationale for the review in the context of existing knowledge. | Introduction |
| Objectives | 4 | Provide an explicit statement of the objective(s) or question(s) the review addresses. | Introduction |
| **METHODS** | | |  |
| Eligibility criteria | 5 | Specify the inclusion and exclusion criteria for the review and how studies were grouped for the syntheses. | Methods section |
| Information sources | 6 | Specify all databases, registers, websites, organisations, reference lists and other sources searched or consulted to identify studies. Specify the date when each source was last searched or consulted. | Methods section |
| Search strategy | 7 | Present the full search strategies for all databases, registers and websites, including any filters and limits used. | Methods section |
| Selection process | 8 | Specify the methods used to decide whether a study met the inclusion criteria of the review, including how many reviewers screened each record and each report retrieved, whether they worked independently, and if applicable, details of automation tools used in the process. | Methods section |
| Data collection process | 9 | Specify the methods used to collect data from reports, including how many reviewers collected data from each report, whether they worked independently, any processes for obtaining or confirming data from study investigators, and if applicable, details of automation tools used in the process. | Methods section |
| Data items | 10a | List and define all outcomes for which data were sought. Specify whether all results that were compatible with each outcome domain in each study were sought (e.g. for all measures, time points, analyses), and if not, the methods used to decide which results to collect. | Methods section & supplement |
|  | 10b | List and define all other variables for which data were sought (e.g. participant and intervention characteristics, funding sources). Describe any assumptions made about any missing or unclear information. | supplement |
| Study risk of bias assessment | 11 | Specify the methods used to assess risk of bias in the included studies, including details of the tool(s) used, how many reviewers assessed each study and whether they worked independently, and if applicable, details of automation tools used in the process. | Methods section & supplement |
| Effect measures | 12 | Specify for each outcome the effect measure(s) (e.g. risk ratio, mean difference) used in the synthesis or presentation of results. | Open data |
| Synthesis methods | 13a | Describe the processes used to decide which studies were eligible for each synthesis (e.g. tabulating the study intervention characteristics and comparing against the planned groups for each synthesis (item #5)). | Methods section & supplement |
|  | 13b | Describe any methods required to prepare the data for presentation or synthesis, such as handling of missing summary statistics, or data conversions. | Methods section & supplement |
|  | 13c | Describe any methods used to tabulate or visually display results of individual studies and syntheses. | Methods section & supplement |
|  | 13d | Describe any methods used to synthesize results and provide a rationale for the choice(s). If meta-analysis was performed, describe the model(s), method(s) to identify the presence and extent of statistical heterogeneity, and software package(s) used. | Methods section & supplement |
|  | 13e | Describe any methods used to explore possible causes of heterogeneity among study results (e.g. subgroup analysis, meta-regression). | Results section |
|  | 13f | Describe any sensitivity analyses conducted to assess robustness of the synthesized results. | Results section, supplement |
| Reporting bias assessment | 14 | Describe any methods used to assess risk of bias due to missing results in a synthesis (arising from reporting biases). | Methods section |
| Certainty assessment | 15 | Describe any methods used to assess certainty (or confidence) in the body of evidence for an outcome. | Methods section |
| **RESULTS** | | |  |
| Study selection | 16a | Describe the results of the search and selection process, from the number of records identified in the search to the number of studies included in the review, ideally using a flow diagram. | Methods section & supplement |
|  | 16b | Cite studies that might appear to meet the inclusion criteria, but which were excluded, and explain why they were excluded. | Supplement |
| Study characteristics | 17 | Cite each included study and present its characteristics. | Open data & Supplement |
| Risk of bias in studies | 18 | Present assessments of risk of bias for each included study. | Risk of bias was not assessed |
| Results of individual studies | 19 | For all outcomes, present, for each study: (a) summary statistics for each group (where appropriate) and (b) an effect estimate and its precision (e.g. confidence/credible interval), ideally using structured tables or plots. | Online data |
| Results of syntheses | 20a | For each synthesis, briefly summarise the characteristics and risk of bias among contributing studies. | Risk of bias was not assessed |
|  | 20b | Present results of all statistical syntheses conducted. If meta-analysis was done, present for each the summary estimate and its precision (e.g. confidence/credible interval) and measures of statistical heterogeneity. If comparing groups, describe the direction of the effect. | Methods section & supplement |
|  | 20c | Present results of all investigations of possible causes of heterogeneity among study results. | Methods section & supplement |
|  | 20d | Present results of all sensitivity analyses conducted to assess the robustness of the synthesized results. | Methods section & supplement |
| Reporting biases | 21 | Present assessments of risk of bias due to missing results (arising from reporting biases) for each synthesis assessed. | Methods section & supplement |
| Certainty of evidence | 22 | Present assessments of certainty (or confidence) in the body of evidence for each outcome assessed. | Methods section & supplement |
| **DISCUSSION** | | |  |
| Discussion | 23a | Provide a general interpretation of the results in the context of other evidence. | Discussion |
|  | 23b | Discuss any limitations of the evidence included in the review. | Discussion & supplement |
|  | 23c | Discuss any limitations of the review processes used. | Discussion & supplement |
|  | 23d | Discuss implications of the results for practice, policy, and future research. | Discussion & supplement |
| **OTHER INFORMATION** | | |  |
| Registration and protocol | 24a | Provide registration information for the review, including register name and registration number, or state that the review was not registered. | End of introduction |
|  | 24b | Indicate where the review protocol can be accessed, or state that a protocol was not prepared. | End of introduction |
|  | 24c | Describe and explain any amendments to information provided at registration or in the protocol. | Supplement |
| Support | 25 | Describe sources of financial or non-financial support for the review, and the role of the funders or sponsors in the review. | Acknowledgements |
| Competing interests | 26 | Declare any competing interests of review authors. | Title page |
| Availability of data, code and other materials | 27 | Report which of the following are publicly available and where they can be found: template data collection forms; data extracted from included studies; data used for all analyses; analytic code; any other materials used in the review. | Online supplement |

*From:*  Page MJ, McKenzie JE, Bossuyt PM, Boutron I, Hoffmann TC, Mulrow CD, et al. The PRISMA 2020 statement: an updated guideline for reporting systematic reviews. BMJ 2021;372:n71. doi: 10.1136/bmj.n71

For more information, visit: <http://www.prisma-statement.org/>

**PRISMA for Abstracts Checklist**

| **Section and Topic** | **Item #** | **Checklist item** | **Reported (Yes/No)** |
| --- | --- | --- | --- |
| **TITLE** | | |  |
| Title | 1 | Identify the report as a systematic review. | Yes |
| **BACKGROUND** | | |  |
| Objectives | 2 | Provide an explicit statement of the main objective(s) or question(s) the review addresses. | Yes |
| **METHODS** | | |  |
| Eligibility criteria | 3 | Specify the inclusion and exclusion criteria for the review. | Not in abstract (space limitation), in main text |
| Information sources | 4 | Specify the information sources (e.g. databases, registers) used to identify studies and the date when each was last searched. | Not in abstract (space limitation), in main text |
| Risk of bias | 5 | Specify the methods used to assess risk of bias in the included studies. | Not in abstract (space limitation), in main text |
| Synthesis of results | 6 | Specify the methods used to present and synthesise results. | Yes |
| **RESULTS** | | |  |
| Included studies | 7 | Give the total number of included studies and participants and summarise relevant characteristics of studies. | Yes |
| Synthesis of results | 8 | Present results for main outcomes, preferably indicating the number of included studies and participants for each. If meta-analysis was done, report the summary estimate and confidence/credible interval. If comparing groups, indicate the direction of the effect (i.e. which group is favoured). | Yes |
| **DISCUSSION** | | |  |
| Limitations of evidence | 9 | Provide a brief summary of the limitations of the evidence included in the review (e.g. study risk of bias, inconsistency and imprecision). | Not in abstract (space limitation), in main text |
| Interpretation | 10 | Provide a general interpretation of the results and important implications. | Yes |
| **OTHER** | | |  |
| Funding | 11 | Specify the primary source of funding for the review. | Not in abstract (space limitation), in acknowledgements |
| Registration | 12 | Provide the register name and registration number. | Not in abstract (space limitation), in methods |

*From:*  Page MJ, McKenzie JE, Bossuyt PM, Boutron I, Hoffmann TC, Mulrow CD, et al. The PRISMA 2020 statement: an updated guideline for reporting systematic reviews. BMJ 2021;372:n71. doi: 10.1136/bmj.n71

# E – References

## E.1 – Studies Included in the Meta-Analysis

Barclay. P. (2015). Unpublished raw data.

Barque-Duran, A., Pothos, E. M., Yearsley, J. M., & Hampton, J. A. (2016). Patterns and

evolution of moral behaviour: moral dynamics in everyday life. *Thinking & Reasoning, 22*(1), 31-56.

Blanken, I., van de Ven, N., & Zeelenberg, M. (2015). A meta-analytic review of moral

licensing. Personality and Social Psychology Bulletin, 41(4), 540-558.

Blanken, I., Van de Ven, N., Zeelenberg, M., & Meijers, M. H. C. (2014). Three attempts to

replicate the moral licensing effect*. Social Psychology, 45,* 232-238. doi:10.1027/1864-9335/ a000189

Boyd, K. E. (2014). The effectsof moral licensing on high-cost and low-cost helping

*behaviours* (Honors thesis, University of Dayton).

Bradley-Geist, J. C., King, E. B., Skorinko, J., Hebl, M. R., & McKenna, C. (2010). Moral

credentialing by associa- tion: The importance of choice and relationship closeness. *Personality and Social Psychology Bulletin, 36*, 1564-1575. doi:10.1177/0146167210385921

Braun, J., & Gollwitzer, M. (2012). Leniency for out‐group offenders. European Journal of

Social Psychology, 42(7), 883-892.

Brown, R. P., Tamborski, M., Wang, X., Barnes, C. D., Mumford, M. D., Connely, S., & Devenport, L. D. (2011). Moral credentialing and the rationalization of misconduct. *Ethics and Behavior, 21,* 1-12. doi:10.1080/10508422.2011.537566

Cascio, J., & Plant, E. A. (2015). Prospective moral licensing: Does anticipating doing good later

allow you to be bad now? *Journal of Experimental Social Psychology, 56,* 110-116. doi:S0022103114001450

Choi, B., Crandall, C. S, & La, S. (2014). Permission to be prejudiced: Legitimacy credits in the

evaluation of advertise- ments*. Journal of Applied Social Psychology, 44*, 190-200. doi:10.1111/jasp.12217

Clot, S., Grolleau, G., & Ibanez, L. (2018). Moral self-licencing and social dilemmas: an

experimental analysis from a taking game in Madagascar. *Applied Economics, 50*(27), 2980-2991.

Clot, S., Grolleau, G., & Ibanez, L. (2013). Self-licensing and financial rewards: is morality for

sale?. Economics Bulletin, 33, 2298-2306.

Conway, P., & Peetz, J. (2012). When does feeling moral actually make you a better person?

Conceptual abstraction moderates whether past moral deeds motivate consistency or compensatory behavior. *Personality and Social Psychology Bulletin, 38*(7), 907-919.

Cornelissen, G., Bashshur, M. R., Rode, J., & Le Menestrel, M. (2013). Rules or consequences:

The role of ethical mind- sets in moral dynamics. *Psychological Science, 24*, 482-488. doi:10.1177/0956797612457377

Cornelissen, G., Karelaia, N., & Soyer, E. (2013, September). Clicktivism or slacktivism? The

ironic effects of symbolic prosocial behavior. Paper presented at Society for the

Advancement of Judgment and Decision Making Studies, Granada, Spain.

de Jong, M. (2017). *Donating and self interest.* (Master’s dissertation, Leiden University).

Ebersole, C. R., Atherton, O. E., Belanger, A. L., Skulborstad, H. M., Allen, J. M., Banks, J. B.,

... & Brown, E. R. (2016). Many Labs 3: Evaluating participant pool quality across the academic semester via replication. *Journal of Experimental Social Psychology, 67*, 68-82.

Effron, D. A., Cameron, J. S., & Monin, B. (2009). Endorsing Obama licenses favoring whites.

Journal of Experimental Social Psychology, 45(3), 590-593.

Effron, D. A., Monin, B., & Miller, D. T. (2012). Inventing racist roads not taken: The licensing

effect of immoral counterfactual behaviors. *Journal of Personality and Social Psychology, 103*, 916-932. doi:10.1037/a0030008

Ferguson, R. (2018). How Flexible is Morality? A Test of the Moral Credits Model of Moral

*Balancing.* (Doctoral dissertation, Australian Catholic University).

Geng, L., Cheng, X., Tang, Z., Zhou, K., & Ye, L. (2016). Can previous pro-environmental

behaviours influence subsequent environmental behaviours? The licensing effect of pro-environmental behaviours. *Journal of Pacific Rim Psychology, 10*, e9.

Gholamzedehmir, M. (2015). The Impact of Moral Action and Moral Values. (Doctoral

dissertation, University of Sussex).

Greene, M., & Low, K. (2014). Public integrity, private hypocrisy, and the moral licensing

effect. Social Behavior and Personality: An International Journal, 42(3), 391-400.

Jones, J. A., & Koenig, A. M. (2018). The effect of “socially moral” purchases on future

financial donations. International Journal of Nonprofit and Voluntary Sector Marketing, 23(4), e1614.

Jordan, J., Mullen, E., & Murnighan, J. K. (2011). Striving for the moral self: The effects of

recalling past moral actions on future moral behavior*. Personality and Social Psychology Bulletin, 37*, 701-713. doi:10.1177/0146167211400208

Lacasse, K. (2019). Can’t hurt, might help: Examining the spillover effects from purposefully

adopting a new pro-environmental behavior. *Environment and Behavior, 51*(3), 259-287.

Lalot, F., Falomir-Pichastor, J. M., & Quiamzade, A. (2018). Compensation and consistency

effects in proenvironmental behaviour: The moderating role of majority and minority support for proenvironmental values. *Group Processes & Intergroup Relations, 21*(3), 403-421.

Lauren, N., Smith, L. D., Louis, W. R., & Dean, A. J. (2019). Promoting spillover: how past

behaviors increase environmental intentions by cueing self-perceptions. *Environment and Behavior, 51*(3), 235-258.

Leonard, B. (2012). Unpublished raw data.

Li, A., Mai, K. M., & Bagger, J. (2017). Licensed to say no: How and why does engaging in a

prior moral action influence family support provision?. *Journal of Vocational Behavior, 102*, 86-98.

Mann, N. H., & Kawakami, K. (2012). The long, steep path to equality: Progressing on

egalitarian goals. *Journal of Experimental Psychology: General, 141*, 187-197. doi:10.1037/a002 5602

Mazar, N., & Zhong, C. B. (2010). Do green products make us better people*? Psychological*

*Science, 21*, 494-498. doi:10.1177/0956797610363538

Meijers, M. H. C., Noordewier, M. K., Verlegh, P. W. J., & Smit, E. G. (2014). Identity

relevance moderates the licensing effect (Chapter from doctoral dissertation).

Meijers, M. H., Verlegh, P. W., Noordewier, M. K., & Smit, E. G. (2015). The dark side of

donating: how donating may license environmentally unfriendly behavior. *Social Influence, 10*(4), 250-263.

Merritt, A. C., Effron, D. A., Fein, S., Savitsky, K. K., Tuller, D. M., & Monin, B. (2012). The

strategic pursuit of moral credentials. *Journal of Experimental Social Psychology, 48*, 774-777. doi:10.1016/j.jesp.2011.12.017

Monin, B., & Miller, D. T. (2001). Moral credentials and the expression of prejudice. *Journal of*

*Personality and Social Psychology, 81*, 33-43. doi:10.1037//0022 3514.81.1.33

Noblet, C. L., & McCoy, S. K. (2018). Does one good turn deserve another? Evidence of

domain-specific licensing in energy behavior. *Environment and Behavior, 50*(8), 839-

863.

Robitaille, N. (2014). An Investigation of Consumers' Moral Licensing Behavior. (Doctoral

dissertation, University of Toronto).

Rotella, A. (2015). The influence of observation on cooperative decision making (Master’s

dissertation, University of Guelph).

Rotella, A., & Barclay, P. (2020). Failure to replicate moral licensing and moral cleansing in an

online experiment. Personality and Individual Differences, 161, 109967.

Sachdeva, S., Iliev, R., & Medin, D. L. (2009). Sinning saints and saintly sinners: The paradox of

moral self-regulation. *Psychological Science, 20*, 523-528. doi:10.1111/j.1467-

9280.2009.02326.x

Schüler, S., Lehnhardt, N., & Huber, M. (2012). Can priming prosocial values eliminate the

moral licensing effect. *Unpublished manuscript*. Technische Universität Dresden, Germany.

Schwabe, M., Dose, D. B., & Walsh, G. (2018). Every saint has a past, and every sinner has a

future: influences of regulatory focus on consumers’ moral self‐regulation. *Journal of Consumer Psychology, 28*(2), 234-252.

Simbrunner, P., & Schlegelmilch, B. B. (2017). Moral licensing: a culture-moderated meta-

analysis. Management Review Quarterly, 67(4), 201-225.

Spektor, M. (2014). Unpublished raw data.

Stellar, J. E., & Willer, R. (2014). The corruption of value: Negative moral associations diminish

the value of money. Social Psychological and Personality Science, 5(1), 60-66.

Susewind, M., & Hoelzl, E. (2014). A matter of perspective: Why past moral behavior can

sometimes encourage and other times discourage future moral striving. *Journal of Applied Social Psychology, 44,* 201-209. doi:10.1111/jasp.12214 Thaler

Szekeres, H. Nouri, R. Saguy, T., & Halperin E. (unpublished) Empathic Credentials: Expressing

Empathy Once Mitigates Subsequent Empathic Concern.

Thomas, J. S., & Showers, C. (2012). Unpublished raw data.

Truelove, H. B., Yeung, K. L., Carrico, A. R., Gillis, A. J., & Raimi, K. T. (2016). From plastic

bottle recycling to policy support: An experimental test of pro-environmental spillover. *Journal of Environmental Psychology, 46*, 55-66.

Urban, J., Bahník, Š., & Kohlová, M. B. (2017). Green consumption does not make people cheat:

Three replications of a moral licensing experiment. Preprint]. Retrieved from https.//doi.org/10/17605/OSF, 10.

Young, Y., Chakroff, A., & Tom, J. (2012). Doing good leads to more good: The reinforcing

power of a moral self-concept. *Review of Philosophy and Psychology, 3,* 325-334.

doi:10.1007/s13164-012-0111-6

Zhong, C. B., Ku, G., Lount, R. B., & Murnighan, J. K. (2009). Compensatory Ethics. *Journal of*

*Business Ethics, 92*, 323-339. doi:10.1007/s10551-009-0161-6

## E.2 – Supplement References

Amir, O., Rand, D. G., & Gal, Y. K. (2012). Economic Games on the Internet: The Effect of $1 Stakes. *PLoS ONE*, *7*(2), e31461. https://doi.org/10.1371/journal.pone.0031461

Aquino, K., Freeman, D., Reed, A., Lim, V. K. G., & Felps, W. (2009). Testing a social-cognitive model of moral behavior: The interactive influence of situations and moral identity centrality. *Journal of Personality and Social Psychology*, *97*(1), 123–141. https://doi.org/10.1037/a0015406

Awad, E., Dsouza, S., Shariff, A., Rahwan, I., & Bonnefon, J.-F. (2020). Universals and variations in moral decisions made in 42 countries by 70,000 participants. *Proceedings of the National Academy of Sciences*, 201911517. https://doi.org/10.1073/pnas.1911517117

Barclay, P. (2014). *Unpublished data*.

Barclay, P. (2016). Biological markets and the effects of partner choice on cooperation and friendship. *Current Opinion in Psychology*, *7*, 33–38. https://doi.org/10.1016/j.copsyc.2015.07.012

Barclay, P. (Unpublished). Licensing toughness. *Unpublsihed Data*.

Barclay, P., & Barker, J. (2020). *Greener Than Thou: People who protect the environment are more cooperative, compete to be environmental, and benefit from reputation*. https://doi.org/10.1016/j.jenvp.2020.101441

Barclay, P., & Willer, R. (2007). Partner choice creates competitive altruism in humans. *Proceedings of the Royal Society B: Biological Sciences*, *274*(1610), 749–753. https://doi.org/10.1098/rspb.2006.0209

Barque-Duran, A., Pothos, E. M., Yearsley, J. M., & Hampton, J. A. (2016). Patterns and evolution of moral behaviour: Moral dynamics in everyday life. *Thinking & Reasoning*, *22*(1), 31–56. https://doi.org/10.1080/13546783.2015.1051585

Blanken, I., vande Ven, N., & Zeelenberg, M. (2015). A Meta-Analytic Review of Moral Licensing. *Personality and Social Psychology Bulletin*, *41*(4), 540–558. https://doi.org/10.1177/0146167215572134

Blanken, I., vande Ven, N., Zeelenberg, M., & Meijers, M. H. C. (2014). Three Attempts to Replicate the Moral Licensing Effect. *Social Psychology*, *45*(3), 232–238. https://doi.org/10.1027/1864-9335/a000189

Borenstein, M. (Ed.). (2009). *Introduction to meta-analysis*. John Wiley & Sons.

Botchway, A. T. (2014). What I achieved versus what I will achieve: The effect of framing on moral behavior licensing. *Unpublished Doctoral Dissertation*.

Boyd, K. E. (2014). The Effects of Moral Licensing on High-Cost and Low-Cost Helping Behaviors. *Honours Thesis*.

Bradley, A., Lawrence, C., & Ferguson, E. (2018). Does observability affect prosociality? *Proceedings of the Royal Society B: Biological Sciences*, *285*(1875), 20180116. https://doi.org/10.1098/rspb.2018.0116

Bradley-Geist, J. C., King, E. B., Skorinko, J., Hebl, M. R., & McKenna, C. (2010a). Moral Credentialing by Association: The Importance of Choice and Relationship Closeness. *Personality and Social Psychology Bulletin*, *36*(11), 1564–1575. https://doi.org/10.1177/0146167210385920

Bradley-Geist, J. C., King, E. B., Skorinko, J., Hebl, M. R., & McKenna, C. (2010b). Moral Credentialing by Association: The Importance of Choice and Relationship Closeness. *Personality and Social Psychology Bulletin*, *36*(11), 1564–1575. https://doi.org/10.1177/0146167210385920

Brañas-Garza, P., Bucheli, M., Paz Espinosa, M., & García-Muñoz, T. (2013). MORAL CLEANSING AND MORAL LICENSES: EXPERIMENTAL EVIDENCE. *Economics and Philosophy*, *29*(2), 199–212. https://doi.org/10.1017/S0266267113000199

Braun, J., & Gollwitzer, M. (2012). Leniency for out-group offenders: Patronizing leniency. *European Journal of Social Psychology*, *42*(7), 883–892. https://doi.org/10.1002/ejsp.1908

Brewer, N. T., Cuite, C. L., Herrington, J. E., & Weinstein, N. D. (2007). Risk compensation and vaccination: Can getting vaccinated cause people to engage in risky behaviors? *Annals of Behavioral Medicine*, *34*(1), 95–99. https://doi.org/10.1007/BF02879925

Brown, R. P., Tamborski, M., Wang, X., Barnes, C. D., Mumford, M. D., Connelly, S., & Devenport, L. D. (2011). Moral Credentialing and the Rationalization of Misconduct. *Ethics & Behavior*, *21*(1), 1–12. https://doi.org/10.1080/10508422.2011.537566

Bühren, C., & Kundt, T. C. (2015). Imagine being a nice guy: A note on hypothetical vs. incentivized social preferences. *Judgment and Decision Making*, *10*(2), 185–190. https://doi.org/10.1017/S1930297500003946

Cain, D. M., Loewenstein, G., & Moore, D. A. (2005). The Dirt on Coming Clean: Perverse Effects of Disclosing Conflicts of Interest. *Journal of Legal Studies*, *34*.

Cain, D. M., Loewenstein, G., & Moore, D. A. (2011). When Sunlight Fails to Disinfect: Understanding the Perverse Effects of Disclosing Conflicts of Interest. *Journal of Consumer Research*, *37*(5), 836–857. https://doi.org/10.1086/656252

Camerer, C. F., Hogarth, R. M., Budescu, D. V., & Eckel, C. (1999). The Effects of Financial Incentives in Experiments: A Review and Capital-Labor-Production Framework. In B. Fischhoff & C. F. Manski (Eds.), *Elicitation of Preferences* (pp. 7–48). Springer Netherlands. https://doi.org/10.1007/978-94-017-1406-8_2

Carattini, S., & Tavoni, A. (2016). How Green Are Green Economists? *Economics Bulletin*, *36*(4), 2311–2323.

Carrico, A. R., Raimi, K. T., Truelove, H. B., & Eby, B. (2018). Putting Your Money Where Your Mouth Is: An Experimental Test of Pro-Environmental Spillover From Reducing Meat Consumption to Monetary Donations. *Environment and Behavior*, *50*(7), 723–748. https://doi.org/10.1177/0013916517713067

Cascio, J., & Plant, E. A. (2015). Prospective moral licensing: Does anticipating doing good later allow you to be bad now? *Journal of Experimental Social Psychology*, *56*, 110–116. https://doi.org/10.1016/j.jesp.2014.09.009

Chiou, W.-B., Yang, C.-C., & Wan, C.-S. (2011). Ironic Effects of Dietary Supplementation: Illusory Invulnerability Created by Taking Dietary Supplements Licenses Health-Risk Behaviors. *Psychological Science*, *22*(8), 1081–1086. https://doi.org/10.1177/0956797611416253

Choi, B., Crandall, C. S., & La, S. (2014). Permission to be prejudiced: Legitimacy credits in the evaluation of advertisements: Legitimacy credits in the evaluation of advertisements. *Journal of Applied Social Psychology*, *44*(3), 190–200. https://doi.org/10.1111/jasp.12217

Clot, S., Grolleau, G., & Ibanez, L. (2013). Self-licensing and financial rewards: Is morality for sale? *Economics Bulletin*, *33*, 2298–2306.

Clot, S., Grolleau, G., & Ibanez, L. (2014). Smug Alert! Exploring self-licensing behavior in a cheating game. *Economics Letters*, *123*(2), 191–194. https://doi.org/10.1016/j.econlet.2014.01.039

Clot, S., Grolleau, G., & Ibanez, L. (2016). Do good deeds make bad people? *European Journal of Law and Economics*, *42*(3), 491–513. https://doi.org/10.1007/s10657-014-9441-4

Clot, S., Grolleau, G., & Ibanez, L. (2018). Moral self-licencing and social dilemmas: An experimental analysis from a taking game in Madagascar. *Applied Economics*, *50*(27), 2980–2991. https://doi.org/10.1080/00036846.2017.1412083

Conway, P., & Peetz, J. (2012). When Does Feeling Moral Actually Make You a Better Person? Conceptual Abstraction Moderates Whether Past Moral Deeds Motivate Consistency or Compensatory Behavior. *Personality and Social Psychology Bulletin*, *38*(7), 907–919. https://doi.org/10.1177/0146167212442394

Cornelissen, G., Bashshur, M. R., Rode, J., & Le Menestrel, M. (2013). Rules or Consequences? The Role of Ethical Mind-Sets in Moral Dynamics. *Psychological Science*, *24*(4), 482–488. https://doi.org/10.1177/0956797612457376

Cornelissen, G., Dewitte, S., Warlop, L., & Yzerbyt, V. (2007). Whatever people say I am, that’s what I am: Social labeling as a social marketing tool. *International Journal of Research in Marketing*, *24*(4), 278–288. https://doi.org/10.1016/j.ijresmar.2007.05.001

Curry, O. S. (2016). Morality as Cooperation: A Problem-Centred Approach. In T. K. Shackelford & R. D. Hansen (Eds.), *The Evolution of Morality* (pp. 27–51). Springer International Publishing. https://doi.org/10.1007/978-3-319-19671-8_2

Curry, O. S., Jones Chesters, M., & Van Lissa, C. J. (2019). Mapping morality with a compass: Testing the theory of ‘morality-as-cooperation’ with a new questionnaire. *Journal of Research in Personality*, *78*, 106–124. https://doi.org/10.1016/j.jrp.2018.10.008

De Witt Huberts, J. C., Evers, C., & De Ridder, D. T. D. (2012). License to sin: Self-licensing as a mechanism underlying hedonic consumption. *European Journal of Social Psychology42*, 490–496.

Effron, D. A. (2014). Making Mountains of Morality From Molehills of Virtue: Threat Causes People to Overestimate Their Moral Credentials. *Personality and Social Psychology Bulletin*, *40*(8), 972–985. https://doi.org/10.1177/0146167214533131

Effron, D. A., & Conway, P. (2015). When virtue leads to villainy: Advances in research on moral self-licensing. *Current Opinion in Psychology*, *6*, 32–35. https://doi.org/10.1016/j.copsyc.2015.03.017

Effron, D. A., & Knowles, E. D. (2015). Entitativity and intergroup bias: How belonging to a cohesive group allows people to express their prejudices. *Journal of Personality and Social Psychology*, *108*(2), 234–253. https://doi.org/10.1037/pspa0000020

Effron, D. A., Miller, D. T., & Monin, B. (2012). Inventing racist roads not taken: The licensing effect of immoral counterfactual behaviors. *Journal of Personality and Social Psychology*, *103*(6), 916–932. https://doi.org/10.1037/a0030008

Effron, D. A., & Monin, B. (2010). Letting People Off the Hook: When Do Good Deeds Excuse Transgressions? *Personality and Social Psychology Bulletin*, *36*(12), 1618–1634. https://doi.org/10.1177/0146167210385922

Effron, D. A., Monin, B., & Miller, D. T. (2013). The unhealthy road not taken: Licensing indulgence by exaggerating counterfactual sins. *Journal of Experimental Social Psychology*, *49*(3), 573–578. https://doi.org/10.1016/j.jesp.2012.08.012

Ek, C. (2015). Some Causes are More Equal than Others? Behavioral Spillovers in Charitable Giving. *Working Paper*.

Engelaar, C. (2017). *Behave like you would or behave like you should: The influence of morality and norms on doing the right thing*. Unpublishd Master’s Thesis. https://openaccess.leidenuniv.nl/bitstream/handle/1887/51162/Engelaar%2C%20Claartje-s1902725-MA%20Thesis%20SOP-2017.pdf?sequence=1

Eskine, K. J., Kacinik, N. A., & Webster, G. D. (2012). The Bitter Truth about Morality: Virtue, Not Vice, Makes a Bland Beverage Taste Nice. *PLoS ONE*, *7*(7), e41159. https://doi.org/10.1371/journal.pone.0041159

Falomir-Pichastor, J. M., Mugny, G., Frederic, N., Berent, J., & Lalot, F. (2018). Motivation to Maintain a Nonprejudiced Identity: The Moderating Role of Normative Context and Justification for Prejudice on Moral Licensing. *Social Psychology*, *49*(3), 168–181. https://doi.org/10.1027/1864-9335/a000339

Ferguson, R. (2019). *How Flexible is Morality? A Test of the Moral Credits Model of Moral Balancing*. Unpublished Doctoral Thesis.

Fishbach, A., & Dhar, R. (2005). Goals as Excuses or Guides: The Liberating Effect of Perceived Goal Progress on Choice. *Journal of Consumer Research*, *32*(3), 370–377. https://doi.org/10.1086/497548

Fiske, S. T., Cuddy, A. J. C., & Glick, P. (2007). Universal dimensions of social cognition: Warmth and competence. *Trends in Cognitive Sciences*, *11*(2), 77–83. https://doi.org/10.1016/j.tics.2006.11.005

Frimer, J., Zhu, L., & Decter-Frain, A. (2015). Do givers leak their social motives through what they talk about? Maybe not. *Manuscript under Review*.

Garvey, A. M., & Bolton, L. E. (2017). Eco-Product Choice Cuts Both Ways: How Proenvironmental Licensing versus Reinforcement is Contingent on Environmental Consciousness. *Journal of Public Policy & Marketing*, *36*(2), 284–298. https://doi.org/10.1509/jppm.16.096

Geng, L., Cheng, X., Tang, Z., Zhou, K., & Ye, L. (2016). Can Previous Pro-Environmental Behaviours Influence Subsequent Environmental Behaviours? The Licensing Effect of Pro-Environmental Behaviours. *Journal of Pacific Rim Psychology*, *10*, e9. https://doi.org/10.1017/prp.2016.6

Gholamzedehmir, M. (2015). The Impact of Moral Action and Moral Values on Moral Judgment and Moral Behaviour. *Unpublished Doctoral Thesis*.

Gneezy, A., Imas, A., Brown, A., Nelson, L. D., & Norton, M. I. (2012). Paying to Be Nice: Consistency and Costly Prosocial Behavior. *Management Science*, *58*(1), 179–187. https://doi.org/10.1287/mnsc.1110.1437

Goodwin, G. P., Piazza, J., & Rozin, P. (2014). Moral character predominates in person perception and evaluation. *Journal of Personality and Social Psychology*, *106*(1), 148–168. https://doi.org/10.1037/a0034726

Graham, J., Haidt, J., Koleva, S., Motyl, M., Iyer, R., Wojcik, S. P., & Ditto, P. H. (2013). Moral Foundations Theory. In *Advances in Experimental Social Psychology* (Vol. 47, pp. 55–130). Elsevier. https://doi.org/10.1016/B978-0-12-407236-7.00002-4

Greene, M., & Low, K. (2014). Public Integrity, Private Hypocrisy, and the Moral Licensing Effect. *Social Behavior and Personality: An International Journal*, *42*(3), 391–400. https://doi.org/10.2224/sbp.2014.42.3.391

Haidt, J., & Joseph, C. (2004). Intuitive ethics: How innately prepared intuitions generate culturally variable virtues. *Daedalus*, *133*(4), 55–66. https://doi.org/10.1162/0011526042365555

Hayley, A., & Zinkiewicz, L. (2013). Does moral cleansing moderate the effect of evolutionary altruism on helping intention? An exploratory study. *Journal of Social, Evolutionary, and Cultural Psychology*, *7*(1), 24–35. https://doi.org/10.1037/h0099176

Ho, B., Taber, J., Poe, G., & Bento, A. (2016). The Effects of Moral Licensing and Moral Cleansing in Contingent Valuation and Laboratory Experiments on the Demand to Reduce Externalities. *Environmental and Resource Economics*, *64*(2), 317–340. https://doi.org/10.1007/s10640-014-9872-y

Hollander, E. P. (1958). Conformity, status, and idiosyncrasy credit. *Psychological Review*, *65*(2), 117–127. https://doi.org/10.1037/h0042501

Ioannidis, J. P. A. (2005). Why Most Published Research Findings Are False. *PLoS Medicine*, *2*(8), e124. https://doi.org/10.1371/journal.pmed.0020124

Irwin, J. R., McClelland, G. H., & Schulze, W. D. (1992). Hypothetical and real consequences in experimental auctions for insurance against low-probability risks. *Journal of Behavioral Decision Making*, *5*(2), 107–116. https://doi.org/10.1002/bdm.3960050203

Jackson, J. C., Halberstadt, J., Takezawa, M., Liew, K., Smith, K. M., Apicella, C. L., & Gray, K. (2023). Generalized Morality Culturally Evolves as an Adaptive Heuristic in Large Social Networks. *Journal of Personality and Social Psychology*, *125*(6), 1207–1238. https://doi.org/10.1037/pspa0000358

Jeong, H.-J., & Koo, D.-M. (2015). Volunteering as a mechanism to reduce guilt over purchasing luxury items. *Journal of Product & Brand Management*, *24*(7), 758–769. https://doi.org/10.1108/JPBM-01-2015-0784

Jones, J. A., & Koenig, A. M. (2018). The effect of “socially moral” purchases on future financial donations. *International Journal of Nonprofit and Voluntary Sector Marketing*, *23*(4), e1614. https://doi.org/10.1002/nvsm.1614

Jordan, A. H., & Monin, B. (2008). From Sucker to Saint: Moralization in Response to Self-Threat. *Psychological Science*, *19*(8), 809–815. https://doi.org/10.1111/j.1467-9280.2008.02161.x

Jordan, J., Mullen, E., & Murnighan, J. K. (2011). Striving for the Moral Self: The Effects of Recalling Past Moral Actions on Future Moral Behavior. *Personality and Social Psychology Bulletin*, *37*(5), 701–713. https://doi.org/10.1177/0146167211400208

Karmarkar, U. R., & Bollinger, B. (2015). BYOB: How Bringing Your Own Shopping Bags Leads to Treating Yourself and the Environment. *Journal of Marketing*, *79*(4), 1–15. https://doi.org/10.1509/jm.13.0228

Khan, U., & Dhar, R. (2006). Licensing Effect in Consumer Choice. *Journal of Marketing Research*, *43*(2), 259–266.

Khan, U., & Dhar, R. (2007). Where there is a way, is there a will? The effect of future choices on self-control. *Journal of Experimental Psychology: General*, *136*(2), 277–288. https://doi.org/10.1037/0096-3445.136.2.277

Klotz, A. C., & Bolino, M. C. (2013). Citizenship and Counterproductive Work Behavior: A Moral Licensing View. *Academy of Management Review*, *38*(2), 292–306. https://doi.org/10.5465/amr.2011.0109

Koritzky, G. (2006). *Inequity aversion: A social utility or a reasonable strategy?* Technion-Israel Institute of Technology, Faculty of Industrial and ….

Kouchaki, M. (2011). Vicarious moral licensing: The influence of others’ past moral actions on moral behavior. *Journal of Personality and Social Psychology*, *101*(4), 702–715. https://doi.org/10.1037/a0024552

Kuper, N., & Bott, A. (2019). Has the evidence for moral licensing been inflated by publication bias? *Meta-Psychology*. https://doi.org/10.15626/MP.2018.878

Lacasse, K. (2019). Can’t Hurt, Might Help: Examining the Spillover Effects From Purposefully Adopting a New Pro-Environmental Behavior. *Environment and Behavior*, *51*(3), 259–287. https://doi.org/10.1177/0013916517748164

Lalot, F., Falomir-Pichastor, J. M., & Quiamzade, A. (2018). Compensation and consistency effects in proenvironmental behaviour: The moderating role of majority and minority support for proenvironmental values. *Group Processes & Intergroup Relations*, *21*(3), 403–421. https://doi.org/10.1177/1368430217733117

Lanzini, P., & Thøgersen, J. (2014). Behavioural spillover in the environmental domain: An intervention study. *Journal of Environmental Psychology*, *40*, 381–390. https://doi.org/10.1016/j.jenvp.2014.09.006

Leonard, B. (2012). *Unpublsihed raw data*.

Li, A., Mai, K. M., & Bagger, J. (2017). Licensed to say no: How and why does engaging in a prior moral action influence family support provision? *Journal of Vocational Behavior*, *102*, 86–98. https://doi.org/10.1016/j.jvb.2017.07.005

Lin, S.-H. (Joanna), Ma, J., & Johnson, R. E. (2016). When ethical leader behavior breaks bad: How ethical leader behavior can turn abusive via ego depletion and moral licensing. *Journal of Applied Psychology*, *101*(6), 815–830. https://doi.org/10.1037/apl0000098

List, J. A., & Gallet, C. A. (2001). What Experimental Protocol Influence Disparities Between Actual and Hypothetical Stated Values? *Environmental and Resource Economics*, *20*, 241–254.

Mann, N. H., & Kawakami, K. (2012). The long, steep path to equality: Progressing on egalitarian goals. *Journal of Experimental Psychology: General*, *141*(1), 187–197. https://doi.org/10.1037/a0025602

May, F., & Irmak, C. (2014). Licensing Indulgence in the Present by Distorting Memories of Past Behavior. *Journal of Consumer Research*, *41*(3), 624–641. https://doi.org/10.1086/676981

Mazar, N., & Zhong, C.-B. (2010). Do Green Products Make Us Better People? *Psychological Science*, *21*(4), 494–498. https://doi.org/10.1177/0956797610363538

Meijers, M. H. C., Verlegh, P. W. J., Noordewier, M. K., & Smit, E. G. (2015). The dark side of donating: How donating may license environmentally unfriendly behavior. *Social Influence*, *10*(4), 250–263. https://doi.org/10.1080/15534510.2015.1092468

Merritt, A. C., Effron, D. A., Fein, S., Savitsky, K. K., Tuller, D. M., & Monin, B. (2012). The strategic pursuit of moral credentials. *Journal of Experimental Social Psychology*, *48*(3), 774–777. https://doi.org/10.1016/j.jesp.2011.12.017

Merritt, A. C., Effron, D. A., & Monin, B. (2010). Moral Self-Licensing: When Being Good Frees Us to Be Bad: Moral Self-Licensing. *Social and Personality Psychology Compass*, *4*(5), 344–357. https://doi.org/10.1111/j.1751-9004.2010.00263.x

Miller, D. T., & Effron, D. A. (2010). Psychological License. In *Advances in Experimental Social Psychology* (Vol. 43, pp. 115–155). Elsevier. https://doi.org/10.1016/S0065-2601(10)43003-8

Monin, B., & Miller, D. T. (2001). Moral Credentials and the Expression of Prejudice. *Journal of Personality and Social Psychology*, *81*(1), 33–43. https://doi.org/10.1037/0022-3514.81.1.33

Mukhopadhyay, A., & Johar, G. V. (2009). Indulgence as self-reward for prior shopping restraint: A justification-based mechanism☆. *Journal of Consumer Psychology*, *19*(3), 334–345. https://doi.org/10.1016/j.jcps.2009.02.016

Mukhopadhyay, A., Sengupta, J., & Ramanathan, S. (2008). Recalling Past Temptations: An Information-Processing Perspective on the Dynamics of Self-Control. *Journal of Consumer Research*, *35*(4), 586–599. https://doi.org/10.1086/591105

Nagel, V. (2014). Three Essays on Moral Self-Regulation of Honesty and Impression Management. *Unpublished Dissertation*.

Noblet, C. L., & McCoy, S. K. (2018). Does One Good Turn Deserve Another? Evidence of Domain-Specific Licensing in Energy Behavior. *Environment and Behavior*, *50*(8), 839–863. https://doi.org/10.1177/0013916517718022

Nowak, M. A., & Sigmund, K. (1998a). Evolution of indirect reciprocity by image scoring. *Nature*, *393*(6685), 573–577.

Nowak, M. A., & Sigmund, K. (1998b). The Dynamics of Indirect Reciprocity. *Journal of Theoretical Biology*, *194*(4), 561–574. https://doi.org/10.1006/jtbi.1998.0775

Ong, M., Mayer, D., & Tost, L. (2014). Does working for a socially responsible organization make employees more or less prosocial? The role of work meaning in reconciling the moral licensing versus consistency debate. *Proceedings of the Annual Meeting of the Academy of Management, Philadelphia*.

Panzone, L. A., Wossink, A., & Southerton, D. (2013). The design of an environmental index of sustainable food consumption: A pilot study using supermarket data. *Ecological Economics*, *94*, 44–55. https://doi.org/10.1016/j.ecolecon.2013.07.003

Polman, E., Pettit, N. C., & Wiesenfeld, B. M. (2013). Effects of wrongdoer status on moral licensing. *Journal of Experimental Social Psychology*, *49*(4), 614–623. https://doi.org/10.1016/j.jesp.2013.03.012

Raska, D. (2010). *Licensing and fluency of sacrosact experience recall*. Unpublished Doctoral Dissertation.

Robitaille, N. (2014). *An Investigation of Consumers’ Moral Licensing Behavior*. Unpublished Doctoral Dissertation. https://tspace.library.utoronto.ca/handle/1807/68220

Rosnow, R. (1991). *Essentials of behavioral research: Methods and data analysis*. New York. NY: MeGraw—Hill, 1nc.

Rotella, A., Fogg, C., Mishra, S., & Barclay, P. (2019). Measuring delay discounting in a crowdsourced sample: An exploratory study. *Scandinavian Journal of Psychology*, *60*(6), 520–527. https://doi.org/10.1111/sjop.12583

Rotella, A. M. (2015). The Influence of Observation on Cooperative Decision Making. *Unpublished Master’s Dissertation*. http://atrium.lib.uoguelph.ca/xmlui/handle/10214/9329

Sachdeva, S., Iliev, R., & Medin, D. L. (2009). Sinning Saints and Saintly Sinners: The Paradox of Moral Self-Regulation. *Psychological Science*, *20*(4), 523–528. https://doi.org/10.1111/j.1467-9280.2009.02326.x

Schwabe, M., Dose, D. B., & Walsh, G. (2018). Every Saint has a Past, and Every Sinner has a Future: Influences of Regulatory Focus on Consumers’ Moral Self-Regulation. *Journal of Consumer Psychology*, *28*(2), 234–252. https://doi.org/10.1002/jcpy.1025

Seçilmiş, E. (2018). An experimental analysis of moral self-regulation. *Applied Economics Letters*, *25*(12), 857–861. https://doi.org/10.1080/13504851.2017.1374530

Shaw, M., Quezada, S. A., & Zárate, M. A. (2011). Violence with a conscience: Religiosity and moral certainty as predictors of support for violent warfare. *Psychology of Violence*, *1*(4), 275–286. https://doi.org/10.1037/a0025346

SimanTov-Nachlieli, I., Shnabel, N., Aydin, A. L., & Ullrich, J. (2018). Agents of Prosociality: Agency Affirmation Promotes Mutual Prosocial Tendencies and Behavior Among Conflicting Groups: Agency Affirmation and Prosociality. *Political Psychology*, *39*(2), 445–463. https://doi.org/10.1111/pops.12418

Simbrunner, P., & Schlegelmilch, B. B. (2017). Moral licensing: A culture-moderated meta-analysis. *Management Review Quarterly*, *67*(4), 201–225. https://doi.org/10.1007/s11301-017-0128-0

Simon, S., & O’Brien, L. T. (2015). Confronting Sexism: Exploring the Effect of Nonsexist Credentials on the Costs of Target Confrontations. *Sex Roles*, *73*(5–6), 245–257. https://doi.org/10.1007/s11199-015-0513-x

Steinhorst, J., & Klöckner, C. A. (2018). Effects of Monetary Versus Environmental Information Framing: Implications for Long-Term Pro-Environmental Behavior and Intrinsic Motivation. *Environment and Behavior*, *50*(9), 997–1031. https://doi.org/10.1177/0013916517725371

Susewind, M., & Hoelzl, E. (2014). A matter of perspective: Why past moral behavior can sometimes encourage and other times discourage future moral striving: A matter of perspective. *Journal of Applied Social Psychology*, *44*(3), 201–209. https://doi.org/10.1111/jasp.12214

Szekeres, H., Nouri, R., Saguy, T., & Halperin, E. (unpublished). *Empathic Credentials: Expressing Empathy Once Mitigates Subsequent Empathic Concern*. Unpublished Manuscript.

Thaler, R. (1985). Mental accounting and consumer choice. *Marketing Science*, *4*(3), 199–214.

Tiefenbeck, V., Staake, T., Roth, K., & Sachs, O. (2013). For better or for worse? Empirical evidence of moral licensing in a behavioral energy conservation campaign. *Energy Policy*, *57*, 160–171. https://doi.org/10.1016/j.enpol.2013.01.021

Truelove, H. B., Yeung, K. L., Carrico, A. R., Gillis, A. J., & Raimi, K. T. (2016). From plastic bottle recycling to policy support: An experimental test of pro-environmental spillover. *Journal of Environmental Psychology*, *46*, 55–66. https://doi.org/10.1016/j.jenvp.2016.03.004

Uhlmann, E. L., Pizarro, D. A., & Diermeier, D. (2015). A Person-Centered Approach to Moral Judgment. *Perspectives on Psychological Science*, *10*(1), 72–81. https://doi.org/10.1177/1745691614556679

Ule, A., Schram, A., Riedl, A., & Cason, T. N. (2009). Indirect Punishment and Generosity Toward Strangers. *Science*, *326*(5960), 1701–1704. https://doi.org/10.1126/science.1178883

Urban, J., Bahník, Š., & Kohlová, M. B. (2019). Green consumption does not make people cheat: Three attempts to replicate moral licensing effect due to pro-environmental behavior. *Journal of Environmental Psychology*, *63*, 139–147. https://doi.org/10.1016/j.jenvp.2019.01.011

West, J., Guelfi, K. J., Dimmock, J. A., & Jackson, B. (2017). “I deserve a treat”: Exercise motivation as a predictor of post-exercise dietary licensing beliefs and implicit associations toward unhealthy snacks. *Psychology of Sport and Exercise*, *32*, 93–101. https://doi.org/10.1016/j.psychsport.2017.06.007

Young, L., Chakroff, A., & Tom, J. (2012). Doing Good Leads to More Good: The Reinforcing Power of a Moral Self-Concept. *Review of Philosophy and Psychology*, *3*(3), 325–334. https://doi.org/10.1007/s13164-012-0111-6

Zhang, S., & Hunt, J. S. (2008). The stereotype rebound effect: Universal or culturally bounded process? *Journal of Experimental Social Psychology*, *44*(3), 489–500. https://doi.org/10.1016/j.jesp.2007.07.010

Zhong, C.-B., Ku, G., Lount, R. B., & Murnighan, J. K. (2010). Compensatory Ethics. *Journal of Business Ethics*, *92*(3), 323–339. https://doi.org/10.1007/s10551-009-0161-6

1. We define a variable as ambiguous if it could be open to more than one interpretation, such that it would be difficult to assess the motives of participants. [↑](#footnote-ref-2)
2. Effect size differences among the manipulation types are compared to the ‘*writing primes’* manipulation subgroup. [↑](#footnote-ref-3)
3. These variables were chosen based on (1) significant moderators, and (2) reviewer comments. We did not include all moderators because that would reduce the power of the analysis. [↑](#footnote-ref-4)
